# Generation of Retinal Pigment Epithelial Cells Derived from Human Embryonic Stem Cells Lacking Human Leukocyte Antigen Class I and II

**DOI:** 10.1016/j.stemcr.2020.02.006

**Published:** 2020-03-19

**Authors:** Sandra Petrus-Reurer, Nerges Winblad, Pankaj Kumar, Laia Gorchs, Michael Chrobok, Arnika Kathleen Wagner, Hammurabi Bartuma, Emma Lardner, Monica Aronsson, Álvaro Plaza Reyes, Helder André, Evren Alici, Helen Kaipe, Anders Kvanta, Fredrik Lanner

**Affiliations:** 1 Clinical Neuroscience, Section for Ophtalmology and Vision, Karolinska Institutet, St. Erik Eye Hospital, 11282 Stockholm, Sweden; 2 Department of Clinical Sciences, Intervention and Technology, Karolinska Insitutet, 17177 Stockholm, Sweden; 3 Gynecology and Reproductive Medicine, Karolinska Universitetssjukhuset, 14186 Stockholm, Sweden; 4 Ming Wai Lau Center for Reparative Medicine, Stockholm Node, Karolinska Institutet, 17177 Stockholm, Sweden; 5 Department of Laboratory Medicine, Karolinska Institutet, 14152 Stockholm, Sweden; 6 Department of Medicine Huddinge, Center for Hematology and Regenerative Medicine, Karolinska Institutet, 17177 Stockholm, Sweden; 7 Clinical Immunology and Transfusion Medicine, Karolinska University Hospital, Huddinge, 14186 Stockholm, Sweden

**Keywords:** human embryonic stem cells, retinal pigment epithelium, cellular therapy, HLA-I knockout, HLA-II knockout, xenograft model, subretinal injection, xenogeneic transplant, immune evasion, transplantation rejection

## Abstract

Human embryonic stem cell-derived retinal pigment epithelial (hESC-RPE) cells could serve as a replacement therapy in advanced stages of age-related macular degeneration. However, allogenic hESC-RPE transplants trigger immune rejection, supporting a strategy to evade their immune recognition. We established single-knockout beta-2 microglobulin (SKO-B2M), class II major histocompatibility complex transactivator (SKO-CIITA) and double-knockout (DKO) hESC lines that were further differentiated into corresponding hESC-RPE lines lacking either surface human leukocyte antigen class I (HLA-I) or HLA-II, or both. Activation of CD4+ and CD8+ T-cells was markedly lower by hESC-RPE DKO cells, while natural killer cell cytotoxic response was not increased. After transplantation of SKO-B2M, SKO-CIITA, or DKO hESC-RPEs in a preclinical rabbit model, donor cell rejection was reduced and delayed. In conclusion, we have developed cell lines that lack both HLA-I and -II antigens, which evoke reduced T-cell responses *in vitro* together with reduced rejection in a large-eyed animal model.

## Highlights

• hESC-RPEs are immunosuppressive but can elicit T-cell and NK cell responses *in vitro*
• hESC-RPEs lacking HLA-I and -II evade T-cell response
• hESC-RPEs lacking HLA-I and -II do not increase NK cell cytotoxic activity
• When xeno-transplanted, these immune-modified hESC-RPEs show reduced rejection

## Teaser

In this article, Lanner and colleagues show that retinal pigment epithelial cells derived from human embryonic stem cells are immunosuppressive, but still activate T and NK cells *in vitro*, and cause rapid rejection in a xenogeneic preclinical model. hESC-RPEs lacking HLA-I and -II neither activate T-cells nor increase NK cell cytotoxicity, and show reduced immune rejection after xeno-transplantation.

## Introduction

Age-related macular degeneration (AMD) affects more than 180 million people globally and is the most common cause of blindness in industrialized countries among people over 60 years (Gehrs et al., 2006, Jonas et al., 2017). A potential regenerative treatment of AMD involves generation of autologous or allogeneic retinal pigment epithelial (RPE) cells from human pluripotent stem cells (hPSCs). Several groups have developed efficient protocols to produce RPE from hPSC morphologically and functionally comparable with native RPE (Choudhary et al., 2017, Hongisto et al., 2017, Klimanskaya et al., 2004, Lane et al., 2014, Maruotti et al., 2015, Osakada et al., 2009, Pennington et al., 2015, Plaza Reyes et al., 2016, Vaajasaari et al., 2011). In addition, several preclinical studies have shown that subretinal suspension or sheet transplants of hPSC-RPE can halt progression and even rescue photoreceptor loss or visual function (Diniz et al., 2013, Idelson et al., 2009, Kamao et al., 2014, Lu et al., 2009, Lund et al., 2006, Petrus-Reurer et al., 2017, Plaza Reyes et al., 2016, Sharma et al., 2019, Vugler et al., 2008), which has encouraged subsequent clinical studies in patients with AMD (da Cruz et al., 2018, Kashani et al., 2018, Mandai et al., 2017, Schwartz et al., 2016, Song et al., 2015).

Although the eye is considered an immune privileged site, immunorejection is frequently observed when transplanting allo- or xenogeneic PSC-RPE to animal models (Ilmarinen et al., 2015, Ilmarinen et al., 2018, McGill et al., 2018, Sohn et al., 2015, Stanzel et al., 2014, Zhang and Bok, 1998). We have previously reported that xenogeneic hESC-RPEs may survive in the subretinal space of albino rabbits for extensive periods under intravitreal steroid immunosuppression, and similar findings were reported for allogeneic RPE derived from induced pluripotent stem cells (iPSCs) transplanted to non-human primates (Petrus-Reurer et al., 2017, Plaza Reyes et al., 2016, Sugita et al., 2017). When rejection occurs it typically involves both the innate immune system, which may activate recipient natural killer (NK) cells by lack of a cognate HLA-I (missing self) (Karre et al., 1986, Ljunggren and Karre, 1990), and the adaptive immune system where an antigen-presenting cell (APC) engulfs the donor cell, processes the mismatched HLA-I and HLA-II molecules into peptides, which in turn are presented and recognized by CD8+ cytotoxic and CD4+ helper T-cells via HLA-I and HLA-II molecules, respectively (indirect allorecognition). In addition, donor cell antigens coupled to HLA-II molecules on the APC activate CD4+ T-cells that also can trigger CD8+ T-cells, NK cells and antibody-producing B cells (Bradley et al., 2002, de Rham and Villard, 2011).

A solution to avoid immune rejection is to use autologous RPEs derived from iPSCs (Mandai et al., 2017). However, such personalized technology is costly and time consuming. An approach suggested to overcome these hurdles has been to establish an iPSC bank of “superdonors,” by donor collection or genome-editing techniques, with a specific homozygous or edited HLA haplotype that could match a high proportion of the population (Deuse et al., 2019, Sugita et al., 2016a, Sugita et al., 2016b, Taylor et al., 2012, Xu et al., 2019).

In this study, we generated and characterized hESCs and hESC-RPEs lacking surface presentation of HLA-I and -II through CRISPR/Cas9 targeting of *B2M* and *CIITA*. Our results support this strategy to overcome host-donor mismatch in non-autologous cell-based treatment of AMD and other hESC-derived cell replacement therapies.

## Results

### B2M and CIITA Loci Edited by CRISPR/Cas9 to Obtain Single- and Double-Knockout Lines

**Figure 1:**
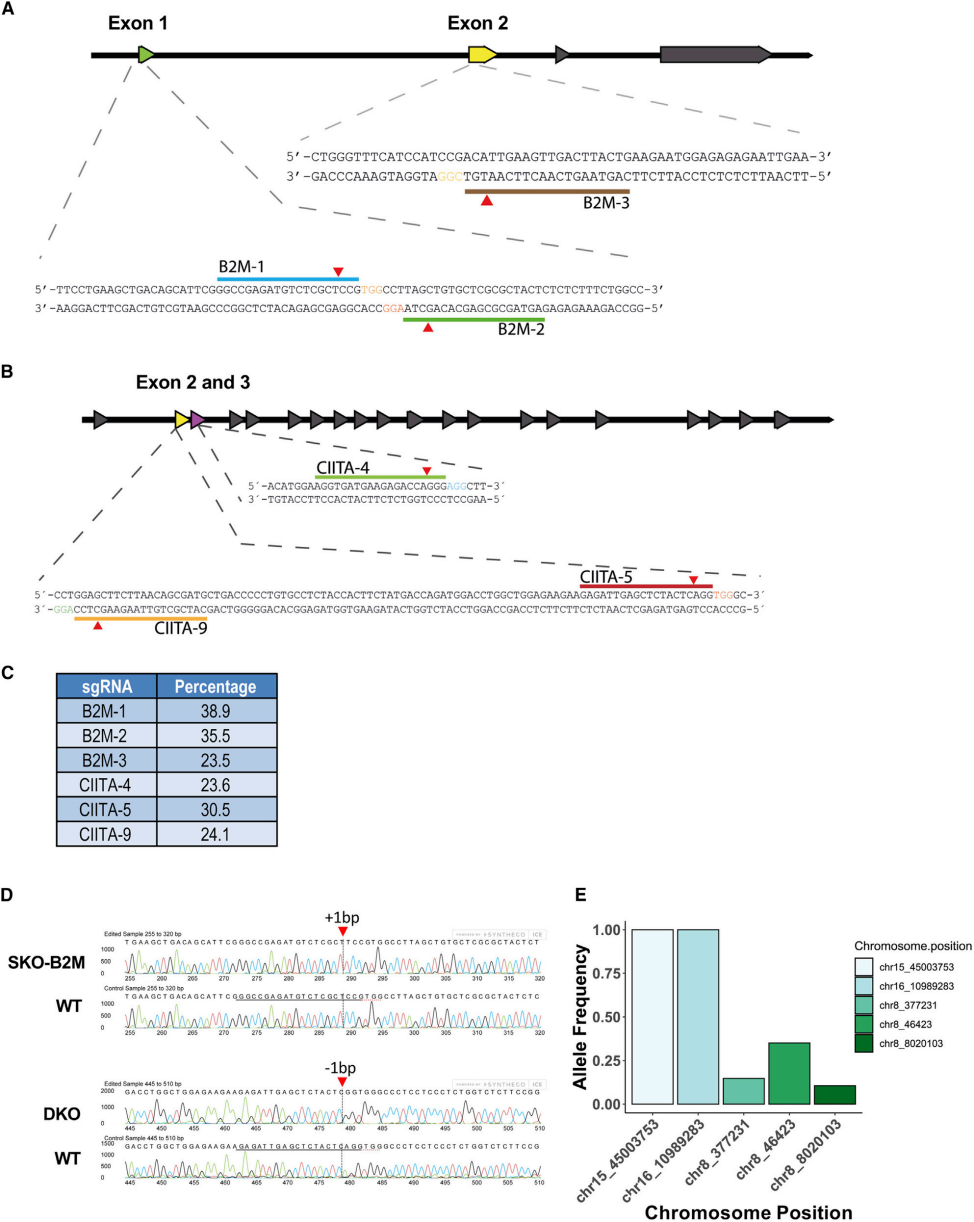
B2M and CIITA sgRNA Evaluation (A) Schematic illustration of the human *B2M* locus, including sgRNA target sites. (B) Schematic illustration of the human *CIITA* locus, including sgRNA target sites. (C) Frequency of indel occurrence generated by each sgRNA in CRISPR/Cas9-edited HEK293T cells. (D) Indel analysis obtained by Sanger sequencing in hESC SKO-B2M (top chromatogram) and hESC DKO (bottom chromatogram). (E) Bar graph representing allele frequency in specific chromosomal positions from off-target analysis of whole-genome sequencing data. See also Figure S1, Tables S3, and S4.

Anchoring of HLA-I molecules to the cell membrane is dependent on the β_2_ microglobulin protein and is encoded by the *B2M* gene (Nathenson et al., 1981). Consequently, loss of B2M leads to failure of HLA-I presentation on the cell surface. CIITA is a well-known HLA-II transactivator that activates HLA-II genes (Masternak et al., 2000). To disrupt their function, we used CRISPR/Cas9 with three sgRNAs targeting exon 1 or 2 of *B2M* (Figures 1A and S1A), or exon 2 or 3 of *CIITA* (Figure 1B), respectively, transfected into HEK293T cells. Insertion/deletions (indels) were detected in all samples, and sgRNAs B2M-1 and CIITA-5 had the highest percentage of cleaved DNA with 38.9% (B2M-1) and 30.5% (CIITA-5) efficiency (Figure 1C). HS980 hESC line was electroporated with pX459-(EF-1α)-B2M-1 (Figure S1B) and all single-cell clones were sequenced to determine the specific on-target mutation. Of note, Cas9 protein presence was not detected at day 9 when cells were plated for clonal expansion (Figure S1C). The hESC single-knockout B2M (hESC SKO-B2M) single-cell clone had a 1-bp insertion predicted to cause a frameshift mutation (Figure 1D, top chromatogram). After *B2M* knockout validation, the hESC SKO-B2M clone was electroporated with pX459-(EF-1α)-CIITA-5. An hESC double-knockout (hESC DKO) *B2M* and *CIITA* single-cell clone that had a 1-bp deletion predicted to cause a knockout of *CIITA* was chosen for further validation (Figure 1D, bottom chromatogram).

We performed paired-end whole-genome sequencing of wild-type hESCs (hESC WT), hESC SKO-B2M, and hESC DKO samples to evaluate putative off-target short nucleotide variants (SNV) and copy-number deletions (Table S3). First, we looked for specific changes at sites predicted by both Cas-OFFinder (Bae et al., 2014) and E-CRISP (Heigwer et al., 2014). The *B2M* gRNA generated 19,277 and *CIITA* gRNA generated 22,618 *in silico* predicted off-targets, respectively. CRISPR/Cas9-induced changes followed by clonal expansion would be expected to result in allele frequencies in line with heterozygote or homozygote changes, such as 0.5 or 1.0, which we also detected at the on-target sites at the *B2M* locus (chr15:45003753; C/CT; AF 1.0) and the *CIITA* locus (chr16:10989283; CA/C; AF 1.0) (Figure 1E). The only additional three changes were detected at lower allelic frequencies, indicating that these instead were acquired changes during culture and unrelated to the CRISPR/Cas9 targeting. Importantly, neither of these were in any known genes. We also searched predicted off-target loci within copy-number deletions and none of the predicted loci were found within homozygous copy-number deletions (Table S4). Moreover, we identified four and three heterozygous copy-number deletions overlapping with the predicted off-target loci for hESC SKO-B2M and hESC DKO samples, respectively, neither of which were in annotated exonic regions. Using an unsupervised approach, we explored if any SNVs had been introduced into known coding genes. This analysis identified 13 (11 SNPs and 2 indels) and 16 (13 SNPs and 3 indels) somatic SNVs within non-redundant exonic boundaries for hESC WT versus SKO-B2M, and SKO-B2M versus DKO samples, respectively; which, after filtering, resulted in three heterozygote SNVs, which were either silent, within the 3ʹ UTR, or a heterozygote nonsense mutation (Table 1). Functionally, neither of these mutations have been linked to disease or tumorigenicity.

**Table 1:** Somatic SNVs Identified Using MuTect2 with Allele Frequency ≥ 0.25 and Read Depth ≥ 10

Chromosome	Position	Reference	Altered	AD	AF	Gene	Type	SNVs	Change
**Sample 1: hESC SKO-B2M**									

chr3	194077829	G	A	16; 14	0.467	LRRC15	3ʹ UTR	SNP	
chr11	64820793	A	G	15; 8	0.348	NAALADL1	Silent	SNP	
chr13	45969047	CTT	C	10; 18	0.63	SLC25A30	3ʹ UTR	DEL	
chr15	22945136	C	T	18; 9	0.346	CYFIP1	Nonsense_Mutation	SNP	ENSP00000324549:p.Gln403^∗^
chr15	45003753	C	CT	0; 24	1	B2M	Frame_Shift_Ins	INS	ENSP00000452780:p.Ser4fs

**Sample 2: hESC DKO**									

chr16	10989283	CA	C	0; 12	1	CIITA	Frame_Shift_Del	DEL	ENSP00000316328:p.Ser66fs

Total non-redundant exonic regions considered in the analysis: 229,235 (26,201 unique genes).

AD, allelic depths for the reference and altered alleles in the order listed; AF, allele fraction of the event in the hESC WT and hESC SKO-B2M (sample 1); or hESC SKO-B2M and hESC DKO (sample 2).

See also Figure S1, Tables S3 and S4.

After targeting the *B2M* locus, we evaluated the HLA-I protein knockout in both hESCs and hESC-RPEs. For that purpose, we decided to increase HLA-I expression by stimulating the cells with interferon gamma (IFN-γ). Titration experiments showed that treatment with 100 ng/mL IFN-γ for 2 days induced high expression of HLA-I in both hESCs and hESC-RPEs, and 5 days induced HLA-II upregulation in hESC-RPEs (Figures S2A and S2B).

**Figure 2:**
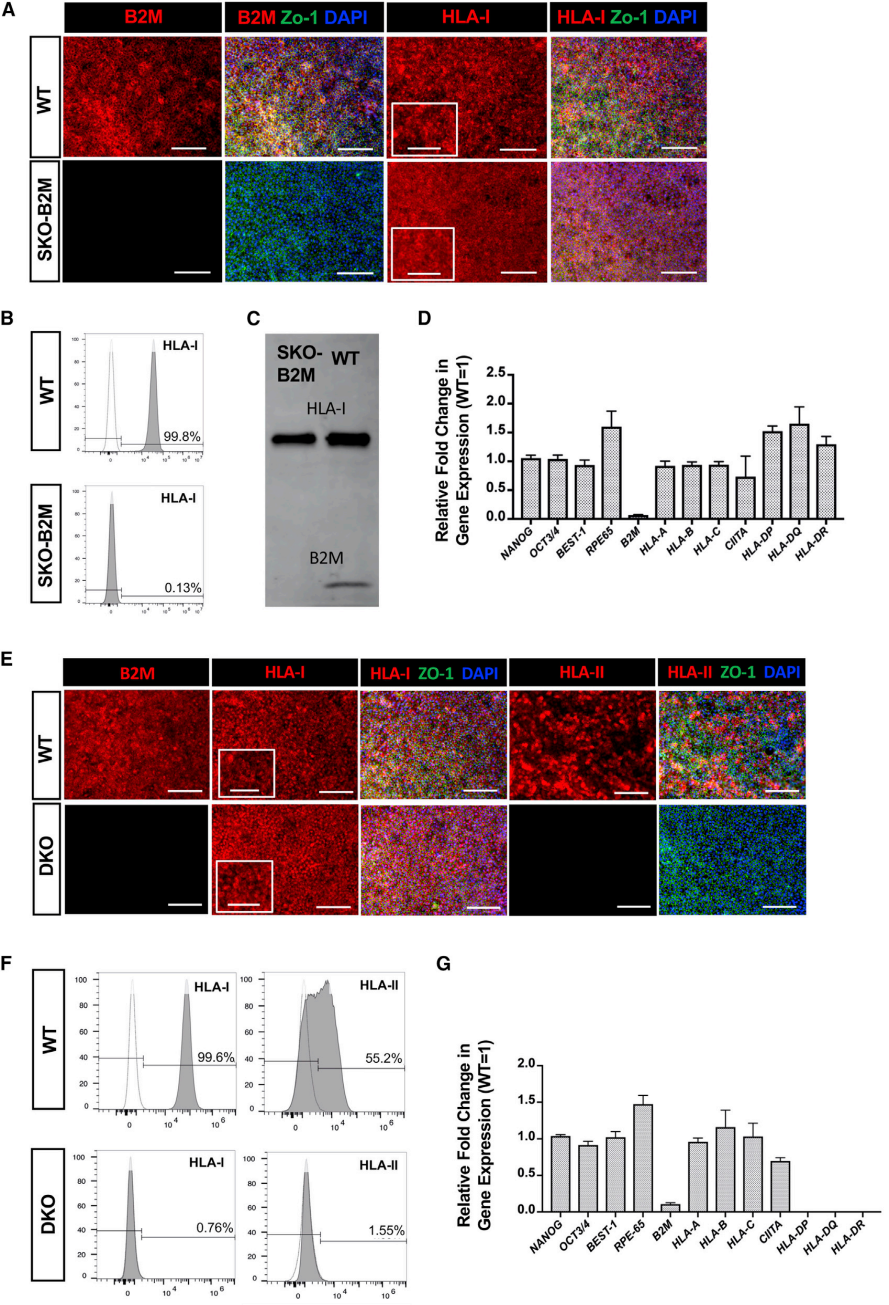
Characterization of the SKO-B2M and DKO hESC-RPEs (A) Immunofluorescence images of WT and SKO-B2M showing B2M, HLA-I, and ZO-1 expression. Magnified box for HLA-I shows dotted extracellular pattern in WT cells. (B) Representative flow cytometry histogram showing the percentage of WT and SKO-B2M expressing extracellular HLA-I. Dotted line histogram shows HLA-I FMO (negative control used for gating). (C) Western blot showing the HLA-I and B2M protein expression of WT and SKO-B2M cells. (D) Gene expression analysis of HLA- and RPE-related genes in the targeted hESC-RPEs. Values are normalized to *GAPDH* and displayed as relative to WT cells. (E) Immunofluorescence images of WT and DKO cells showing B2M, HLA-I, and ZO-1 expression. Magnified box for HLA-I shows dotted extracellular pattern in WT cells. (F) Representative flow cytometry histogram showing the percentage of WT and DKO cells expressing extracellular HLA-I. Dotted line histogram shows HLA-I FMO (negative control used for gating). (G) Gene expression analysis of pluripotent and HLA-related genes in the targeted hESC-RPEs. Values are normalized to *GAPDH* and displayed as relative to WT. Bars represent mean ± SEM from three independent experiments. Scale bars, 100 μm (A and E) and 50 μm zoom-in (A and E). Molecular weight of HLA-I = 43 kDa; B2M = 12 kDa. See also Figure S2.

Differentiated SKO-B2M hESC-RPEs showed characteristic pigmentation, cobblestone morphology, and expression of the epithelial marker ZO-1 (Figure 2A). Moreover, B2M was lost in SKO-B2M cells and HLA-I was not present on the cell surface, as shown by immunofluorescence and flow cytometry (Figures 2A–2D); although the intracellular molecule was detected in the cytoplasm (Figures 2A and 2C). Gene expression of pluripotency-, RPE-, and HLA-I and -II-related genes in SKO-B2M cells did not differ from hESC-RPE WT, indicating that the targeting did not affect any of the downstream phenotypical properties of hESC-RPE cells (Figure 2D). DKO hESC-RPEs stimulated with IFN-γ for 5 days also showed cobblestone morphology, expression of ZO-1 and intracellular HLA-I but lack of B2M (Figure 2E). Moreover, neither intra- nor extracellular HLA-II were present in DKO cells as indicated by immunofluorescence and flow cytometry (Figures 2E and 2F). At a transcriptomic level, pluripotency genes (*NANOG* and *OCT3/4*), RPE genes (*BEST-1* and *RPE-65*), and HLA-I-related genes (*HLA-A*, *-B*, *-C*, except for *B2M* as for SKO-B2M cells) did not differ from WT. HLA-II-related genes (*HLA-DR*, *-DP*, *-DQ*) showed no expression, whereas *CIITA* was unaltered in DKO cells (Figure 2G).

### hESC-RPEs Show Immunomodulatory Properties

**Figure 3:**
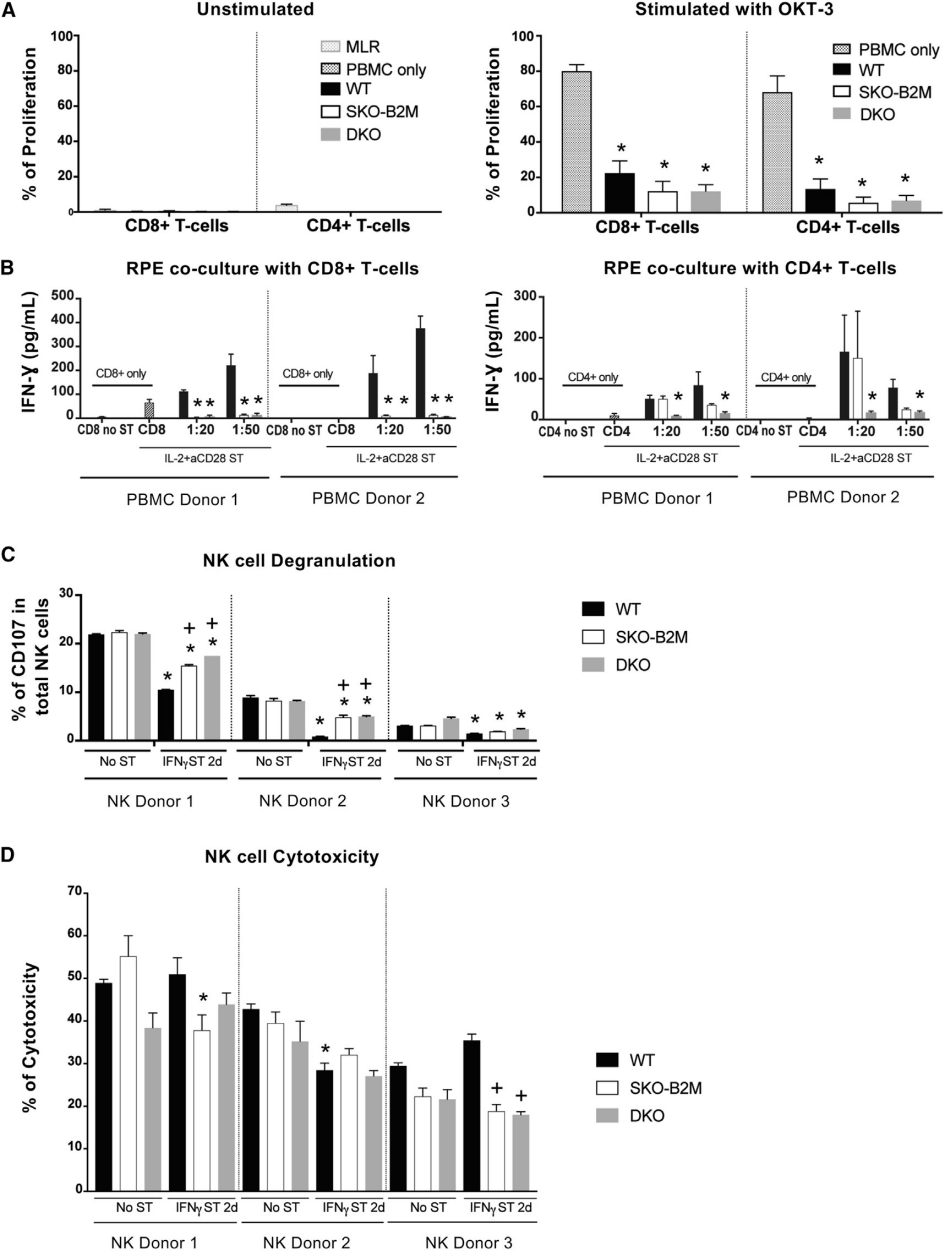
*In Vitro* Immunogenicity Assessment of WT, SKO-B2M, and DKO hESC-RPEs (A) Graphs representing the percentage of proliferative CD8+ or CD4+ cells upon 5 days co-culture of PBMCs from three different donors with 2 days IFN-γ 100 ng/mL pre-stimulated WT, SKO-B2M, and DKO cells at 1:1 hESC-RPE:PBMC ratio with (right panel) or without (left panel) OKT-3 stimulation. Unstimulated PBMCs only and mixed allogenic PBMC donors (mixed lymphocyte reactions [MLR]) were used as negative and positive controls, respectively, to assess T-cell induction upon hESC-RPE co-culture; and OKT-3 stimulated PBMCs were used as positive control to evaluate suppression of T-cell proliferation upon hESC-RPE co-culture. (B) Bar graphs showing the secretion of IFN-γ produced by either CD8+ or CD4+ isolated T-cells from two different donors after 5 days co-culture with WT, SKO-B2M, or DKO cells at 1:20 and 1:50 ratios (hESC-RPE:PBMC) with the presence of IL-2+aCD28 stimulation (and 2 days IFN-γ 100 ng/mL hESC-RPE pre-stimulation). CD8+ or CD4+ only were used as negative controls unstimulated (No ST) or IL2+aCD28 stimulated (ST). (C) Bar graphs showing the percentage of NK degranulation by CD107-positive expression in the total NK cells when co-cultured with WT, SKO-B2M, and DKO cells unstimulated or 2 days IFN-γ 100 ng/mL pre-stimulation from three different donors. (D) Bar graph showing the percentage of cytotoxicity of the unstimulated or 2 days IFN-γ 100 ng/mL pre-stimulated WT, SKO-B2M, or DKO cells (target) measured by chromium release of the killed cells by the freshly isolated and overnight IL-2-stimulated NK cells (effector) from three different donors at 10:1 effector:target ratio. NK cells were freshly isolated from human blood PBMCs, further separated (CD56 MACS isolation kit) and activated with IL-2 overnight before co-culture with hESC-RPEs. Bars represent mean ± SEM from three independent experiments. (A) ^∗^p < 0.0001 compared with PBMC only with OKT-3; (B) ^∗^p < 0.0001 (CD8+), ^+^p < 0.05 (CD4+) compared with respective WT; (C) ^∗^p < 0.0001 compared with respective No ST cell line per donor, ^+^p < 0.0001 compared with respective WT per donor; and (D) ^∗^p < 0.01 compared with respective No ST cell line per donor, ^+^p < 0.001 compared with respective WT per donor. See also Figures S3 and S4.

We then evaluated the immunosuppressive capacity of the cells by co-culturing WT, SKO-B2M, or DKO cells with isolated human peripheral blood mononuclear cells (PBMCs) at a 1:1 ratio, with or without OKT-3 (anti-CD3 monoclonal antibody) stimulation. Notably, addition of hESC-RPEs to unstimulated PBMCs did not induce T-cell proliferation in any of the lines, whereas allogeneic PBMC control induced measurable proliferative responses (Figure 3A). Upon PBMC stimulation with OKT-3, WT and both SKO-B2M and DKO cells significantly decreased the proliferative response of CD8+ and CD4+ T-cells by 5- to 10-fold compared with stimulated PBMCs alone, demonstrating the general immunosuppressive ability of the derived RPE cells (Figure 3A).

### SKO-B2M and DKO hESC-RPEs Exhibit Abolished T-Cell Reactivity *In Vitro*

Next, we assessed whether the hESC-RPEs could promote T-cell response under inflammatory conditions. hESC-RPEs were pre-stimulated with IFN-γ 100 ng/mL for 2 days before co-culturing with PBMCs. This treatment alone did not induce T-cell proliferation (data not shown). However, when the T-cell stimulatory signals interleukin-2 (IL-2) (1 ng/mL) and anti-CD28 antibody (aCD28, 1.25 μg/mL) were added to the co-culture, we observed up to a 4-fold increase in CD8+ T-cell proliferation compared with PBMCs only, at ratios of 1:20 and 1:50 of hESC-RPEs to PBMCs (Figure S3A). Purified CD8+ T-cells but not CD4+ T-cells, showed significant reduction of IFN-γ secretion in the presence of SKO-B2M cells compared with WT; whereas IFN-γ amounts were reduced by both CD8+ and CD4+ T-cells when cultured with DKO cells, thus confirming abolished activation of cytotoxic and helper T-cells (Figure 3B). Further analysis of T-cell ligands expressed on the surface of both hESCs and hESC-RPEs demonstrated that PD-L1, but not PD-L2, was robustly detected in WT and DKO cells both under non-stimulated and stimulated conditions (Figure S3B). In addition, after IFN-γ stimulation, a small population in all analyzed hESC-RPE lines expressed the co-stimulatory molecule CD80 but not CD86, which was instead present to a greater degree in hESCs. The expression of co-stimulatory molecules could potentially give hESC-RPE APC properties and, consequently, the ability to mount a T-cell reaction by themselves (Figure S3B).

### SKO-B2M and DKO hESC-RPEs Increase NK Cell Activation but Not Cytotoxicity under Inflammatory Conditions *In Vitro*

In co-cultures with freshly isolated and overnight IL-2-activated human NK cells, we first found that HLA-I levels were induced with hESC-RPE differentiation (data not shown) coinciding with older hESC-RPEs (days d55–d63) being less sensitive to NK cell cytotoxicity than younger cells (days d44–d50) and hESCs at a 10:1 effector:target ratio (Figure S3C). Degranulation analysis measured by CD107 expression upon co-culture of hESC-RPEs and overnight IL-2-activated NK cells showed a donor-dependent response, stronger in NK donor 1 compared with NK donors 2 and 3 (Figure 3C). Of note, NK cells from all three donors showed no degranulation in the absence of target cells, and showed robust stimulation with positive controls K562 target cells (erythroid leukemia) or phorbol-myristate-acetate/ionomycin stimulation (chemical stimulation) (Figure S3D). The edited SKO-B2M and DKO cell lines did not differ in NK cell activation compared with WT in non-inflammatory conditions (Figure 3C). Interestingly, IFN-γ pre-stimulation of hESC-RPEs showed a significantly decreased response in all analyzed donors, especially for WT cells relative to their unstimulated samples; and the edited lines showed a significantly increased response compared with their respective stimulated WT hESC-RPE co-cultures (Figure 3C). In addition, when NK cells were mixed with unstimulated SKO-B2M or DKO cells at a 10:1 effector:target ratio, cytotoxicity levels also showed to be donor dependent, and strikingly similar between WT and edited lines lacking HLA-I or HLA-I and -II molecules (Figures 3D and S3D). IFN-γ (100 ng/mL) pre-stimulation of hESC-RPE for 2 days did not show any reproducible significant effect among the three donors, although a trend in reduced cytotoxicity could be appreciated in donor 2, in addition to a general maintenance or decrease in cytotoxicity in the edited lines compared with their respective WT hESC-RPE co-cultures (Figure 3D). Further evaluation of several NK cell ligands by flow cytometry showed that HLA-I (including HLA-C) was expressed in hESCs and WT cells, and this expression increased after 2 or 5 days of IFN-γ stimulation. Other ligands tested included MIC-A/B (recognized by the activating NK cell receptor NKG2D) that showed a slightly higher expression in hESCs than in hESC-RPEs; proliferating cell nuclear antigen (recognized by the activating NK cell receptor NKp30) that was not upregulated in any of the cell types; CD112 (a ligand for the activating receptor DNAM-1 and the inhibitory receptor TIGIT) that was highly expressed in all cell types tested; and CD155 (another ligand for DNAM-1, TIGIT, and CD96, which has been ascribed with both activating and inhibitory functions) that was expressed at lower levels in both SKO-B2M and DKO compared with WT cells and IFN-γ stimulatory conditions (Figure S3E). In addition, WT cells exhibit significant expression levels of CD47, CD55, and CD59, which have been suggested previously to function as inhibitors of NK cell activity (Deuse et al., 2019, Finberg et al., 1992, Marcenaro et al., 2003) (Figure S4A). Furthermore, analysis of NKG2A expression and several killer-cell immunoglobulin-like receptors (KIR)—KIR2DL1, KIR3DL1 KIR2DL5, KIR3DL2, and KIR2DL2/L3/S2 (clone [CH-L]) that recognize different HLA molecules on the donor NK cells showed a donor distinct pattern (Figure S4B), which could contribute to the different susceptibility of activation and killing potential of the donor NK cells. As expected, expression of NKG2A (binding to the HLA-E molecule) was consistently elevated in the three donors compared with the remaining KIRs that were analyzed. Because the hESCs we used express ligands for KIR2DL1 (HLA-C^∗^04), KIR2DL2 (HLA-C^∗^07), KIR3DL1 (HLA-A^∗^32, HLA-B^∗^38), KIR3DL2 (HLA-A^∗^68), and NKG2A (HLA-E), we studied the degranulation response of NK cells expressing the specific KIRs by the hESC-RPEs. With our panel of antibodies, we were able to categorize NK cells expressing KIR2DL1, KIR2DL2 (detected by KIR2DL2/L3/S2), KIR3DL1, and NKG2A. As in the analysis of total NK cells, KIR+ NK cells showed variation in CD107a expression between the donors (Figures S4B and S4C). However, a reduction in response toward WT cells stimulated with IFN-γ could be observed in all KIR+ NK cells from all donors (Figure S4C). This inhibition was less potent in SKO-B2M and DKO cells, probably due to a failure to upregulate HLA-I in response to IFN-γ stimulation.

### Immunogenicity of WT hESC-RPEs upon Subretinal Injection in a Xenograft Model

**Figure 4:**
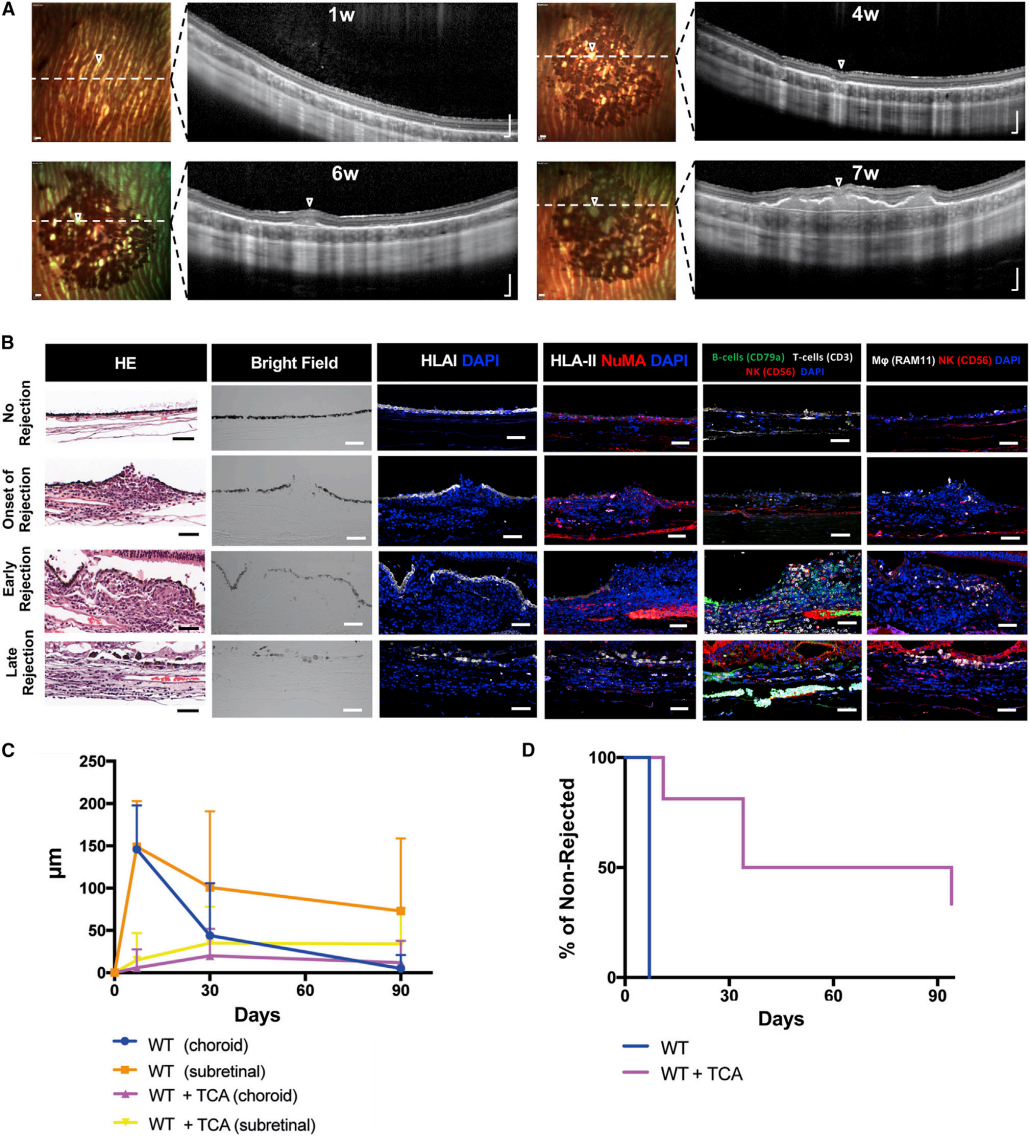
Transplantation of WT hESC-RPEs in the Xenograft Model (A) Time course multicolor-confocal scanning laser ophthalmoscopy and SD-OCT images of the injected areas with WT cells in the subretinal space under TCA treatment. Dashed white lines indicate SD-OCT scan plane. Open arrowheads indicate focal areas of rejection. (B) Bright-field, H&E, and immunofluorescence images representing different rejection patterns after subretinal injection of WT cells. Images show the expression of human HLA-I, HLA-II, and NuMA, in addition to rabbit immune cells: CD3 for T-cells, CD56 for NK cells, CD79a for B cells, and RAM11 for macrophages. (C) Graph showing the thickness of either the choroidal or the subretinal space of eyes transplanted with WT cells with or without TCA immunosuppression through time (days d0, d30, d60, and d90). The rejection thickness was obtained by subtracting the values of a non-rejected area as described in the Experimental Procedures section. (D) Kaplan-Meyer graph representing the number of non-rejected eyes up to 90 days after transplantation of WT cells or WT cells + TCA. Bars represent mean ± SEM from WT = 18; WT + TCA = 15 eyes for all time points in (C) (the value of four eyes in the d90 time point was carried forward from the last observation due to their planned enucleation at d30); and WT = 18; WT + TCA = 15 eyes in (D). Scale bars, 200 μm (A) and 50 μm (B).

We have previously shown that WT cells can integrate in the subretinal space of albino rabbits under intravitreal immunosuppression with triamcinolone (TCA) (Plaza Reyes et al., 2016). However, we also noted that integration efficiency varied considerably, and pigmented areas of integrated cells were frequently lost over time, suggestive of immunorejection. We analyzed this in more detail by observing evidence of cell infiltration in the transplanted area on consecutive spectral-domain optical coherence tomography (SD-OCT) scans. As expected, subretinally transplanted WT cells under TCA immunosuppression displayed homogeneous monolayer integration as demonstrated by a distinct pigmented area on multicolor-confocal scanning laser ophthalmoscopy and a hyperreflective subretinal layer on SD-OCT (Figure 4A). However, at later time points, a pronounced mass was progressively formed in the subretinal space that displaced the pigmented donor cell layer and was accompanied by a thickening of the choroid, suggestive of immunorejection (Figure 4A; Video S1). Histological analysis confirmed that infiltrates consisted of densely packed, mainly mononuclear, inflammatory cells (Figure 4B). As the reaction progressed, the pigmented donor cells were gradually lost, and the subretinal infiltrate and choroidal thickening regressed, eventually leaving an atrophic retina and choroid (Figures 4B and 4C). Immune cells from both the innate (NK cells and macrophages) and the adaptive (CD3+ T-cells) immune system were present at different rejection stages compared with non-rejected eyes, whereas B cells (CD79a^+^) emerged in early and late stages but not at the onset of rejection (Figure 4B). Human HLA-I was expressed in the transplanted WT cells even in later stages when cell shape and integrity had deteriorated, whereas HLA-II only appeared positive in cases of more prominent rejection (early and late) (Figure 4B). We next compared the rejection rates over time of WT transplanted animals using SD-OCT. In non-immunosuppressed animals, rejection was detected in all eyes within 7 days after transplantation, whereas in TCA-treated animals, subretinal infiltration was rarely seen after 1 week, and even after 30 days only half of the transplanted eyes showed signs of rejection (Figures 4C and 4D). By 90 days, rejection was observed in most of the transplanted eyes, also with TCA immunosuppression.

Video S1. Movie Showing SD-OCT Scans 7 Weeks after Injection of WT hESC-RPEs in the Subretinal Space of the Rabbit Eye, Related to Figure 4

### Immunogenicity of SKO-B2M, SKO-CIITA, and DKO hESC-RPEs upon Subretinal Injection in a Xenograft Model

**Figure 5:**
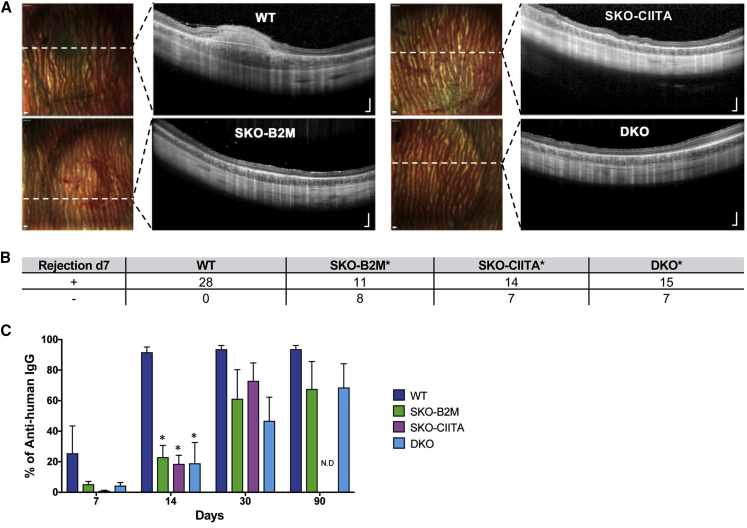
Transplantation of SKO-B2M, SKO-CIITA, and DKO hESC-RPEs in the Xenograft Model (A) Day 7 multicolor-confocal scanning laser ophthalmoscopy and SD-OCT images of representative rabbits that received WT, SKO-B2M, SKO-CIITA, or DKO cells without TCA treatment. Dashed white lines indicate the SD-OCT scan plane. (B) Table summarizing the number of rejected or non-rejected eyes at day 7 upon transplantation of WT, SKO-B2M, SKO-CIITA, or DKO cells. (C) Bar graph showing the percentage of anti-human immunoglobulin G antibodies measured by flow cytometry present in rabbit serums after transplantation of WT, SKO-B2M, SKO-CIITA, or DKO without TCA that bound to WT cells at 7, 14, 30, and 90 days. Bars represent mean ± SEM from five rabbits per condition. In (B), ^∗^p < 0.001 compared with WT; and (C), ^∗^p < 0.001 compared with respective WT per time point. Scale bars, 200 μm (A). N.D., no data available. See also Figure S5.

We next assessed the immune response after subretinal transplantation of SKO-B2M and DKO without TCA immunosuppression in the xenogeneic model. Both types of gene-edited cells showed significantly lower rates of early (1 week) rejection compared with WT cells (Figures 5A and 5B). Interestingly, DKO cells did not survive better than SKO-B2M in the *in vivo* setting (Figure 5B). To examine the role specifically of HLA-II we also established single-knockout *CIITA* hESC-RPEs (SKO-CIITA) that lacked HLA-II expression while still being able to present HLA-I (Figures S5A–S5D). SKO-CIITA cells also showed a significantly reduced rejection, similar to the cells lacking HLA-I or HLA-I and -II (Figures 5A and 5B). Immune cells from both the innate (NK cells and macrophages) and the adaptive (CD3+ T-cells and CD79a+ B cells) immune system were present 7 days after subretinal transplantation of WT cells, unlike DKO where CD79a+ cells were not detected (Figures S5E and S5G). Regardless, all immune cell types were present 30 days after transplantation of either SKO-B2M or DKO cells (Figures S5F and S5H). Furthermore, serum from transplanted rabbits was collected at different time points and evaluated by flow cytometry for presence of anti-human (graft) antibodies (Figure 5C). Without immunosuppressive treatment, anti-human antibodies were detected 7 days after WT transplantation, but were only detectable after 14 days (in 4-fold significantly lower levels compared with WT) in SKO-B2M-, SKO-CIITA-, and DKO-transplanted animals. After 30 and 90 days, anti-human antibodies were widely detected in all transplantation conditions, albeit at lower amounts in the gene-edited compared with WT cells (Figure 5C). Thus, both early and late xenorejection of either SKO-B2M, SKO-CIITA, or DKO were delayed compared with WT cells.

## Discussion

We have shown that targeting *B2M* and *CIITA* genes in hESCs using CRISPR/Cas9 technology eliminates cell surface presentation of HLA-I and HLA-II proteins, respectively. In addition, these cells can be further differentiated into hESC-RPEs that also lack HLA-I, HLA-II, or both HLA-I and -II presentation but retain native RPE cell properties. We also show that SKO-B2M cells reduce CD8+ but not CD4+ T-cell activation, that DKO reduce both CD8+ and CD4+ T-cell activation, and that both lines increase NK cell activation but not cytotoxic activity compared with WT cells under inflammatory conditions. When transplanted into the rabbit xenograft model without immunosuppression, all gene-edited lines reduce early rejection rates and delay anti-human antibody production associated with late rejection.

Engineering cells introduces the risk of off-target changes. Nonetheless, it is encouraging that we did not detect any signs of deleterious mutations or deletions in the predicted off-target sites after *B2M* or *CIITA* targeting neither defective properties in morphology nor characteristic marker expression in either SKO-B2M or DKO cells, suggesting that the mutational load is modest during our genome editing procedure. Absence of predicted off-target changes may be linked to the fact that we have performed clonal selection, which may eliminate less frequent off-target editing that may be detected if the initial bulk culture would have been expanded without clonal selection. However, changes in the genome can also occur during clonal selection and subsequent culture. Indeed, we did detect four heterozygote changes in known genes that may be spontaneous or CRISPR/Cas9 induced. Irrespectively, it is important to note that neither of these heterozygous changes have been linked to disease or tumorigenicity.

Despite the immunosuppressive phenotype of RPE cells, our study also shows that they can still induce a T-cell response under certain conditions *in vitro*: (1) that a specific RPE:immune cell ratio is achieved and (2) that inflammatory/stimulatory molecules, such as IFN-γ or IL-2 are present. In our setting, we show that HLA-I and HLA-II mismatched cells can trigger cytotoxic CD8+ and helper CD4+ T-cells, which would eventually lead to both acute and chronic graft rejection. However, if neither HLA molecule is present, T-cell response is clearly avoided. Interestingly, we found a population of approximately 5% among the IFN-γ-stimulated hESC-RPEs that showed upregulation of the co-stimulatory ligand CD80, which would confer the ability to induce an immune response without APC.

Our data suggest that the “missing self” phenotype (lack of HLA-I or both HLA-I and -II) of the engineered hESC-RPEs does increase the innate NK cell activation of the mismatched HLA-I and/or HLA-II WT cells. Such phenotype was observed only after inflammatory stimulation of the hESC-RPEs, but further studies are needed to explore if this is specific for hESC-RPEs despite their immunosuppressive properties. This finding suggests that the incorporation of an NK cell inhibitory ligand, such as HLA-E or the recently suggested cell surface molecules CD47, CD55, and CD59, may be beneficial to avoid rejection of grafted cells by the innate immune system (Deuse et al., 2019, Finberg et al., 1992, Gornalusse et al., 2017, Marcenaro et al., 2003, Sugita et al., 2018). Interestingly, our derived hESC-RPEs endogenously express high levels of CD47, CD55, and CD59, which could in part explain the lack of difference in NK cell cytotoxicity regardless of the HLA edits. Our degranulation assay showed a distinct difference in responsiveness dependent on donor, which correlated with the outcome of the cytotoxic assay. The level of activation might be subjected to NK cell education, i.e., expression of specific KIRs that recognize the presence of certain HLA molecules, such as HLA-C2, for the 2DL1 KIR present in the donor NK cells (Pegram et al., 2011). However, the degree and differences seen in degranulation and cytotoxicity when HLA-I was absent (SKO-B2M or DKO) or increased (IFN-γ stimulation), could be explained by the changes in expression of certain ligands (e.g., CD155), and may be specific to hESC-RPEs. Overall, NK cell activation toward RPE cells was limited to a few donors, and did not result in measurable release of cytokines (IFN-γ) (data not shown). In addition, these NK studies show no difference in NK cell cytotoxicity after removal of HLA-I or both HLA-I and -II in normal or inflammatory conditions, thus suggesting that HLA-II has no effect on NK cells and reinforcing the possibility to use these double gene-edited cells to maximize the escape from both T and NK cells. Nonetheless, another possible strategy to counteract HLA-I- and -II-independent NK cell effect could reside on the selection of a donor hESC-RPE line from a cell bank based on reactivity with the recipient NK cells, aiming to avoid activation of NK cells that could elicit strong responses when the specific HLA epitopes are missing in the hESC-RPEs.

Without immunosuppression, donor cells triggered rapid rejection in our xenogeneic transplantation model, whereas TCA-mediated immunosuppression delayed and sometimes prohibited rejection. The degree and time of rejection varied greatly, implying that local factors were involved. For instance, mechanical damage to Bruch's membrane during transplantation could compromise the immune-privileged subretinal space, thus allowing invasion by immune cells as suggested previously (Petrus-Reurer et al., 2017). The cells infiltrating into the graft belonged to both the innate and adaptive immune systems in all stages of rejection. Evaluation of the T-cell subtypes (CD8+ or CD4+) in the infiltrates could not be performed due to lack of reliable immunoreaction with anti-rabbit antibodies but deserves further exploration since a reduction in CD8+ cells after SKO-B2M transplantation, and a reduction of both CD8+ and CD4+ T-cells after DKO transplantation, would be expected, which could explain the milder rejection phenotype compared with WT cells. Contrary to what we expected from the *in vitro* results, transplantation of DKO cells was not superior to either SKO-B2M or SKO-CIITA, possibly suggesting that either CD4+ T-cells are pre-activated (i.e., rendering HLA-II loss ineffective) or that, despite delayed T-cell-mediated rejection, NK cells may eventually counter react it. The fact that all hESC-RPE lines showed rejection over time may be due to antibodies produced against human cells via indirect allorecognition (macrophages, dendritic cells, or B lymphocytes recognizing grafted cells) that activate CD4+ T-cells (de Rham and Villard, 2011, Fox et al., 2001, McGill et al., 2018, Sohn et al., 2015, Sugita et al., 2017).

Our *in vitro* and *in vivo* observations show that DKO cells have the potential to partially evade the adaptive immune system, making these cells attractive candidates as universal donors. Nevertheless, the innate immune system, including NK cells, may still be activated as discussed above. It is important to note that the immune system is an essential first-line defense against tumor formation and infection, and generation of immune evasive cells could also escape this defense mechanism. Therefore, a fail-safe/suicidal cassette system enabling the clearance of engineered cells may have to be integrated before clinical consideration (Liang et al., 2018). Also, minor antigens and sugars that do not require HLA molecule presentation or fragments of dead grafted cells could be also be a source of APC recognition and immune activation.

In this study, donor cells were transplanted in a cross-species model, whereas in a comparable clinical setting donor cells would be allogenic where rejection is expected to be less pronounced (Fox et al., 2001). It is well known from other models that xenogeneic rejection involves not only allogeneic pathways but also innate neutrophil and macrophage responses toward non-self oligosaccharides that precede T-cell activation. The stronger activation of the innate immune system may in turn explain why conventional immunosuppressants are less effective in preventing xenogeneic rejection (Hawthorne et al., 2016). The rabbit immune system is in this respect poorly studied but it is reasonable to infer the involvement of xeno-specific rejection pathways, as suggested by our data. Therefore, our finding that removal of HLA-I, HLA-II, or both HLA-I and -II delayed rejection is encouraging since a reduced rejection, as observed with either gene-edited hESC-RPEs, is also expected to translate into an allogeneic human setting. However, and ideally, the immune reactions demonstrated in this study should also be confirmed in a preclinical allogeneic model, in line with the previously demonstrated observations in non-human primates transplanted with allogeneic WT iPSC-RPEs (Sugita et al., 2017).

In conclusion, we show that WT hESC-RPEs can induce both adaptive and innate immunoresponses *in vitro* and *in vivo*, but the loss of surface HLA-I and HLA-II molecules leads to a decreased immunogenicity, thus comprising a first step toward universal-donor cells for cell replacement therapy in AMD and related conditions.

## Experimental Procedures

### Cell Culture and Differentiation

hESC line HS980 was established and cultured under xeno-free and defined conditions on rhLN-521, and passaged as described previously (Rodin et al., 2014a, Rodin et al., 2014b). For differentiation, cells were plated at a density of 2.4 × 10^4^ cells/cm^2^ on 20 μg/mL hrLN-111-coated dishes using NutriStem hESC XF medium and Rho-kinase inhibitor during the first 24 h. NutriStem hESC XF without basic fibroblast growth factor and transforming growth factor β was then replaced and from day 6 after plating, 100 ng/mL of activin A was added to the medium for a total of 5 weeks.

### Genome Editing of hESCs

hESC line HS980 was modified at *B2M* and *CIITA* loci using CRISPR/Cas9 plasmid constructs. First, high cutting efficiency sgRNAs were identified through screening in HEK293T. Second, gene editing in hESCs was first done at the B2M locus, followed by puromycin selection and clonal expansion. An SKO-B2M clone was then edited at the CIITA locus, followed by puromycin selection and clonal expansion. For both of the generated cell lines, on-target edits were determined by Sanger sequencing, and the characterization was based on detection of HLA-I and -II proteins under inflammatory conditions (IFN-γ, 100 ng/mL). Finally, potential off-target edits were analyzed by whole-genome sequencing.

### T-Cell and NK Cell Co-cultures with hESC-RPE Lines

PBMCs were isolated from buffy coats by density gradient centrifugation. PBMCs were either stained using the CellTrace CFSE Cell Proliferation Kit; or CD4+, CD8+, and CD56+ negatively isolated with MACS beads and co-cultured with day 30 unstimulated or 2 day IFN-γ (100 ng/mL) pre-stimulated irradiated or chromium-labeled hESC-RPEs plated at a cell density range of 1 × 10^3^ (1:500) to 5.5 × 10^5^ (1:1) cells/cm^2^ depending on the assay. Overnight IL-2-activated hNK cells were used for ^51^Cr release and degranulation assays and assay-dependent stimulatory molecules were added. Co-cultures were maintained for 4 h (degranulation, cytotoxicity) or 5 days (T-cell proliferation) at 37°C.

### Subretinal Transplantation and *In Vivo* Imaging

Dissociated hESC-RPEs were injected (50 μL; 50,000 cells) subretinally using a transvitreal pars plana technique. SD-OCT and confocal scanning laser ophthalmoscopy was performed to obtain horizontal cross-sectional B-scans and en face fundus *in vivo* images, respectively.

## Author Contributions

S.P.-R., N.W., L.G., M.C., A.K.W., E.L., A.P.R., H.B., and A.K. performed the experiments. S.P.-R. performed *in vitro* and *in vivo* experiments. N.W. derived the gene-edited lines with CRISPR/Cas9. P.K. performed off-target analysis. H.B., M.A., and H.A. contributed to the animal work. H.K., H.A., and E.A. planned the experiments and contributed to discussions. S.P.R., N.W., A.K., and F.L. planned the experiments, analyzed the data, and wrote the article.

## Supplemental Information

Document S1. Supplemental Experimental Procedures, Figures S1–S5, and Tables S1–S3

Table S4. Total Homozygous Deletions Identified for hESC WT versus hESC SKO-B2M, and hESC SKO-B2M versus hESC DKO Samples, Related to Figure 1

Document S2. Article plus Supplemental Information
