# Supplementary material for: Generation of Retinal Pigment Epithelial Cells Derived from Human Embryonic Stem Cells Lacking Human Leukocyte Antigen Class I and II
Source: Stem Cell Reports. 2020 Mar 19;14(4):648–62. doi: 10.1016/j.stemcr.2020.02.006 (PMC7160308; doi:10.1016/j.stemcr.2020.02.006)
Supplement: Document S1. Supplemental Experimental Procedures, Figures S1–S5, and Tables S1–S3 [file mmc1.pdf]

**Stem Cell Reports, Volume *14***

## Supplemental Information

# Generation of Retinal Pigment Epithelial Cells Derived from Human Embryonic Stem Cells Lacking Human Leukocyte Antigen Class I and II

**Sandra Petrus-Reurer, Nerges Winblad, Pankaj Kumar, Laia Gorchs, Michael Chrobok, Arnika Kathleen Wagner, Hammurabi Bartuma, Emma Lardner, Monica Aronsson, Álvaro Plaza Reyes, Helder André, Evren Alici, Helen Kaipe, Anders Kvanta, and Fredrik Lanner**

## SUPPLEMENTAL ITEMS

# Generation of retinal pigment epithelial cells derived from human embryonic stem cells lacking human leukocyte antigen class I and II

**Petrus-Reurer et al.**

**Supplemental Figures 1 – 5**

**Supplemental Video 1**

**Supplemental Tables 1 – 4**

**Experimental Procedures**

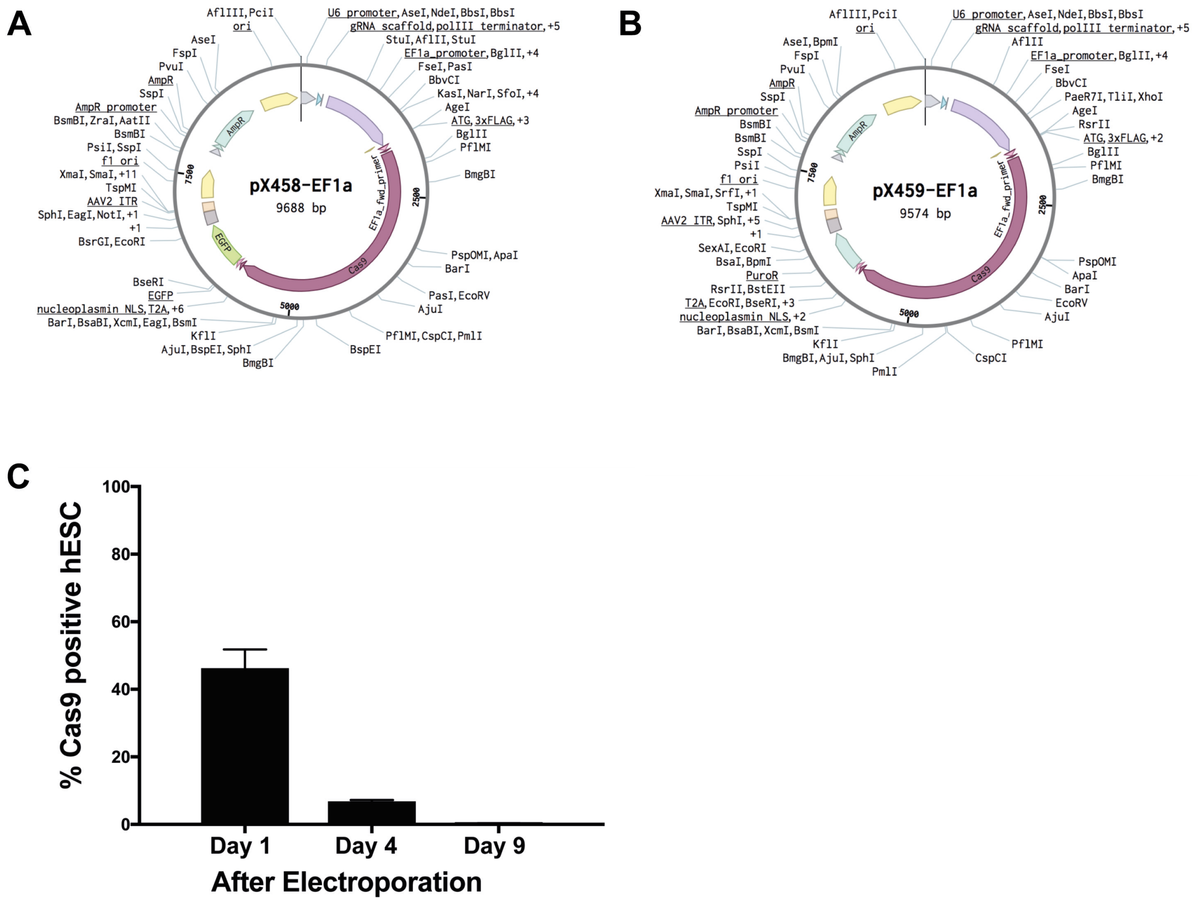


**FIGURE S1. Modified CRISPR/Cas9 plasmids and evaluation of persistence of Cas9 in cells after electroporation. Related to Figure 1.**

(A) Plasmid map of the final construct of pX458 containing the EF1$\alpha$ promoter. This plasmid was used during sgRNA screening. (B) Plasmid map of the final construct of pX459 containing the EF1$\alpha$ promoter. This plasmid was used in hESCs. Both plasmid maps (A, B) were generated using Benchling [Biology Software] (2016) and retrieved from https://benchling.com. (C) Bar graph showing percentage of cells still expressing Cas9 after one, four or nine days after electroporation shown by flow cytometry.

Bars represent mean±SEM from 3 independent experiments.

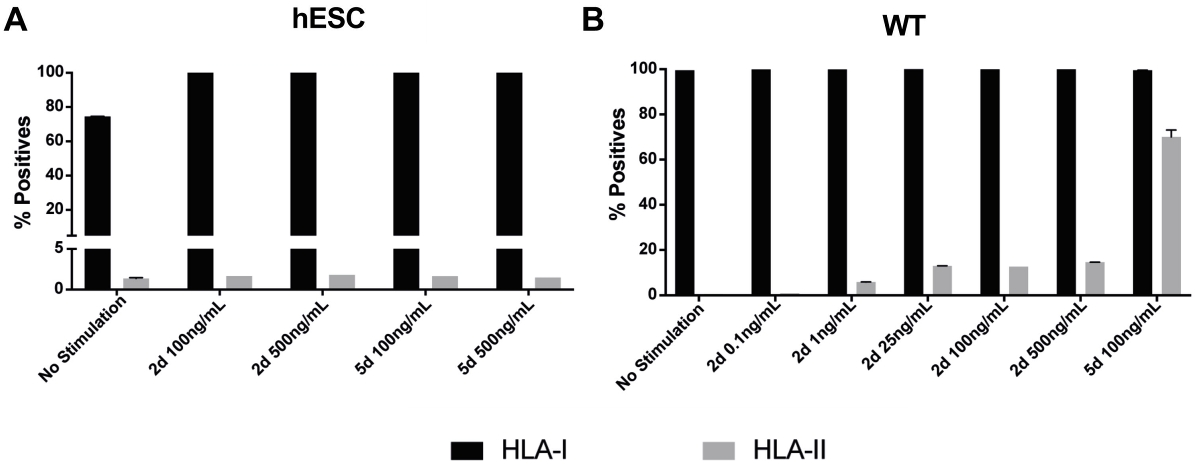


**FIGURE S2. IFN-γ stimulation titration in hESC and hESC-RPE. Related to Figure 2.**

(A) Bar graphs showing the percentage of HLA-I and HLA-II positive hESC cells measured by flow cytometry in different stimulatory conditions. (B) Bar graphs showing the percentage of HLA-I and HLA-II positive hESC-RPE cells measured by flow cytometry in different IFN-γ stimulatory conditions.

Bars represent mean±SEM from 3 independent experiments.

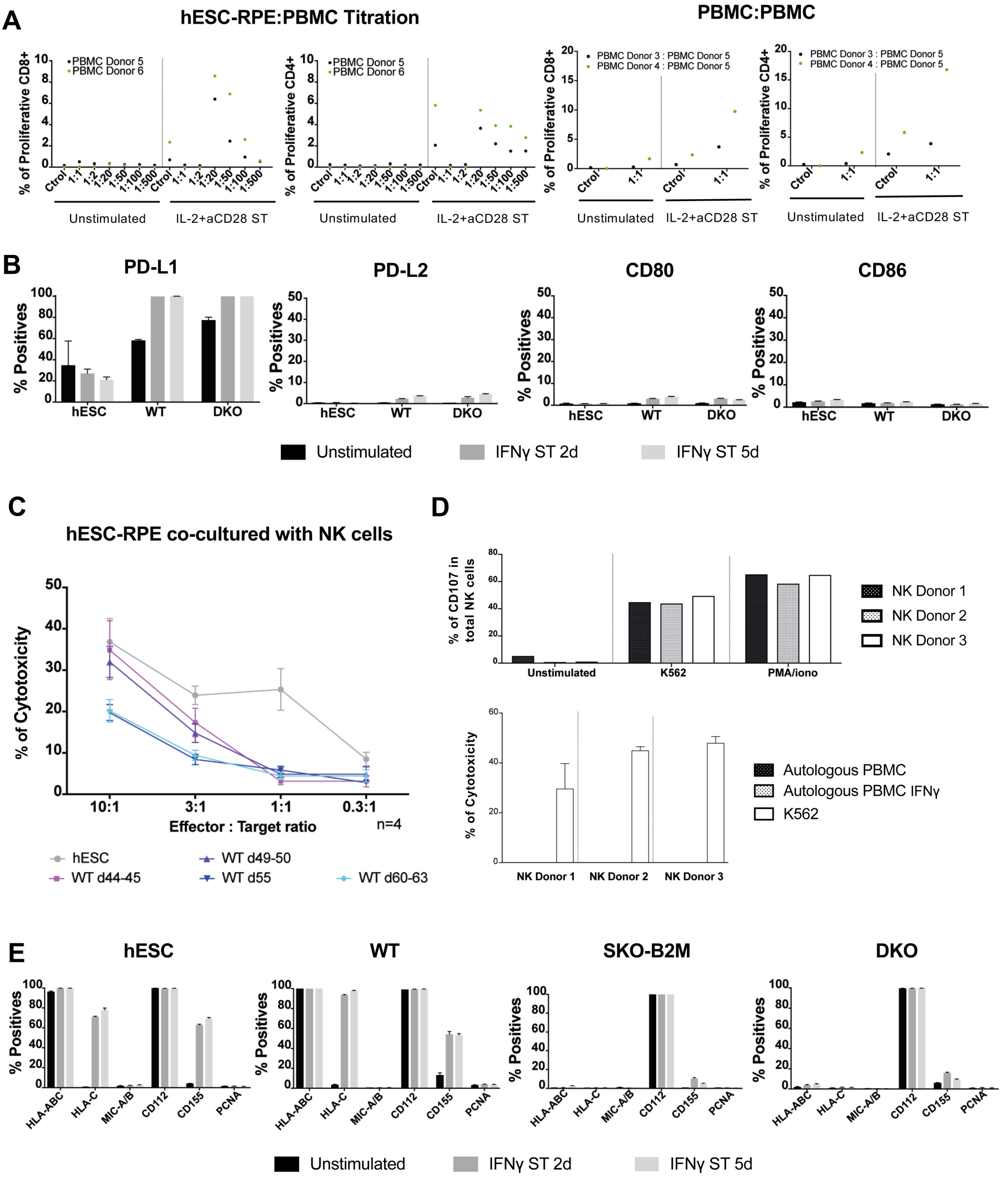
A
hESC-RPE:PBMC Titration
PBMC:PBMC
% of Proliferative CD8+
% of Proliferative CD4+
PBMC Donor 5
PBMC Donor 6
PBMC Donor 3 : PBMC Donor 5
PBMC Donor 4 : PBMC Donor 5
Ctrol
1:1
1:2
1:20
1:50
1:100
1:500
Unstimulated
IL-2+aCD28 ST
B
PD-L1
PD-L2
CD80
CD86
% Positives
hESC
WT
DKO
Unstimulated
IFNγ ST 2d
IFNγ ST 5d
C
hESC-RPE co-cultured with NK cells
% of Cytotoxicity
10:1
3:1
1:1
0.3:1
Effector : Target ratio
n=4
hESC
WT d44-45
WT d49-50
WT d55
WT d60-63
D
% of CD107 in total NK cells
Unstimulated
K562
PMA/iono
NK Donor 1
NK Donor 2
NK Donor 3
% of Cytotoxicity
Autologous PBMC
Autologous PBMC IFNγ
K562
E
hESC
WT
SKO-B2M
DKO
% Positives
HLA-ABC
HLA-C
MIC-A/B
CD112
CD155
PCNA
Unstimulated
IFNγ ST 2d
IFNγ ST 5d

**FIGURE S3.** ***In vitro*** **immunogenicity assessment of WT, SKO-B2M and DKO hESC-RPE. Related to Figure 3.**

(A) Bar graph showing the percentage of proliferative $CD8^+$ or $CD4^+$ cells upon 5 days co-culture of PBMCs from 2 different donors with WT cells at several hESC-RPE:PBMC ratios (1:1; 1:2; 1:20; 1:50; 1:100; 1:500) without (Unstimulated) or with the presence of IL-2+aCD28 stimulation (and 2 days IFN-γ 100 ng/mL hESC-RPE pre-stimulation) (hESC-RPE:PBMC Titration, left panel). PBMC:PBMC (mixed lymphocyte reaction, as T-cell proliferation control, right panel) shows the percentage of proliferative $CD8^+$ or $CD4^+$ cells upon 5 days co-culture of PBMCs from different donors at 1:1 ratio without (Unstimulated) or with the presence of IL-2+aCD28 stimulation. PBMCs only were used as negative controls (Ctrol). (B) Bar graphs showing the percentage of positive cells expressing co-inhibitory T-cell ligands PD-L1 and PD-L2, and co-stimulatory molecules CD80 and CD86 measured by flow cytometry in hESC, WT and SKO-B2M cells unstimulated or stimulated 2 or 5 days with IFN-γ 100 ng/mL. (C) Graph showing the percentage of cytotoxicity of hESC and WT cells at different maturity stages/days (target) measured by chromium release of the killed cells by the freshly isolated and overnight IL-2 stimulated NK cells (effector) of 4 different donors. Several target:effector cell ratios were tested (10:1; 3:1; 1:1; 0.3:1). (D) Bar graphs showing the percentage of NK degranulation shown by CD107 positive expression in total NK cells when cultured without target cells (unstimulated), with K562 cells or with phorbol-myristate-acetate/Ionomycin (PMA/Iono) from 3 different donors (upper panel); and the percentage of cytotoxicity of autologous PBMCs, 100 ng/mL IFN-γ over-night stimulated autologous PBMCs or K562 cells measured by chromium release of the killed cells by the freshly isolated and overnight IL-2 stimulated NK cells of 3 different donors (lower panel). (E) Bar graphs showing the percentage of positive cells expressing of NK cell ligands HLA-ABC, HLA-C, MIC-A/B, CD112, CD155 and PCNA measured by flow cytometry in hESC, WT, SKO-B2M and DKO cells unstimulated or stimulated 2 or 5 days with IFN-γ 100ng/mL.

Bars represent mean±SEM from 3 (B, D, E) or 4 (C) independent experiments.

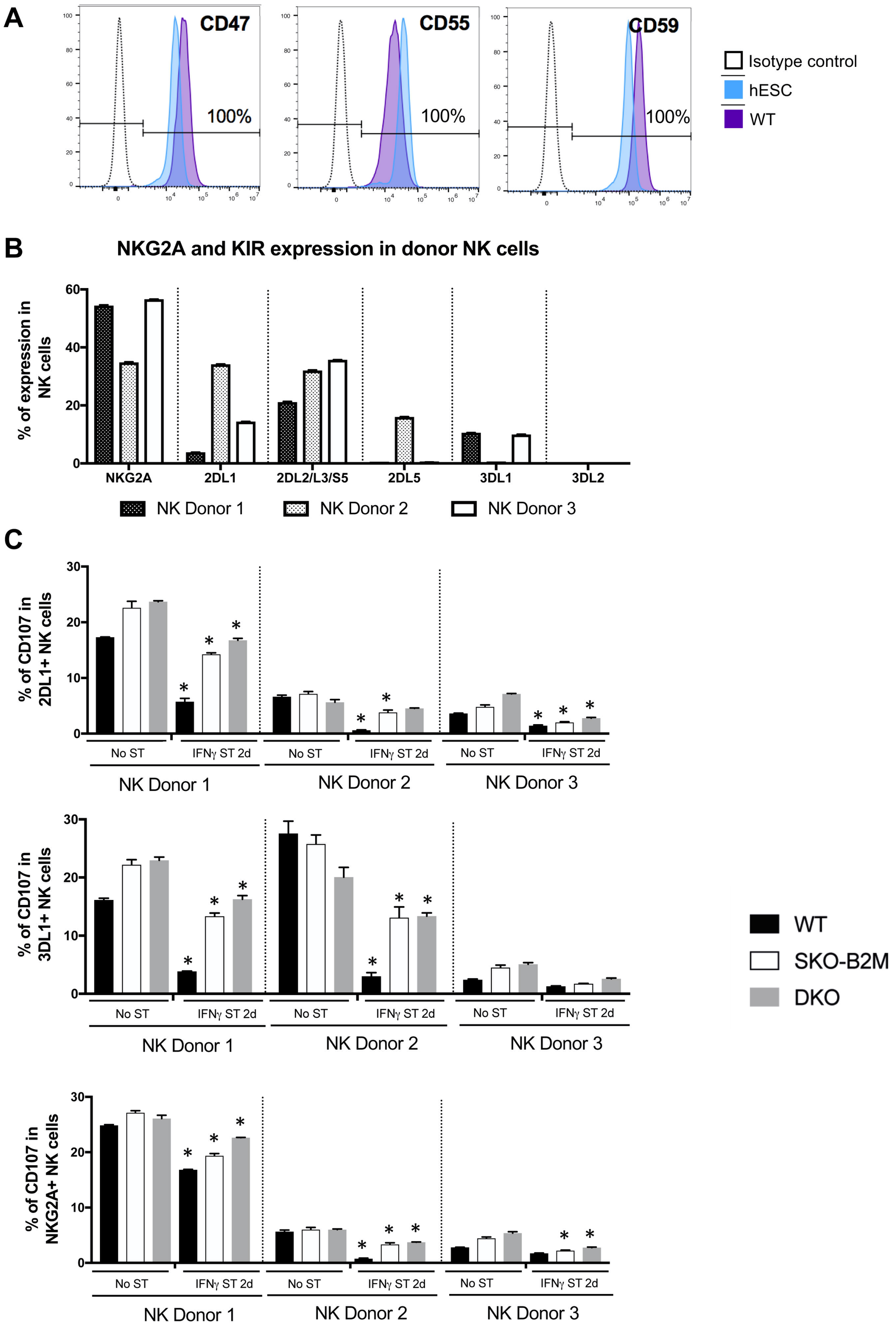

A
CD47
CD55
CD59
100%
Isotype control
hESC
WT
B
NKG2A and KIR expression in donor NK cells
% of expression in NK cells
NKG2A
2DL1
2DL2/L3/S5
2DL5
3DL1
3DL2
NK Donor 1
NK Donor 2
NK Donor 3
C
% of CD107 in 2DL1+ NK cells
% of CD107 in 3DL1+ NK cells
% of CD107 in NKG2A+ NK cells
No ST
IFNγ ST 2d
NK Donor 1
NK Donor 2
NK Donor 3
WT
SKO-B2M
DKO

**FIGURE S4.** ***In vitro*** **immunogenicity assessment of WT, SKO-B2M and DKO hESC-RPE. Related to Figure 3.**

(A) Representative flow cytometry histogram showing the percentage of hESC and WT cells expressing extracellular CD47, CD55 and CD59. Dotted line histogram shows respective mIgG isotype control (negative control used for gating). (B) Bar graphs showing the percentage of expression of NKG2A and several different KIR receptors (2DL1, 2DL2/L3/S5, 2DL5, 3DL1 and 3DL2) from 3 different donors. hESC-RPE haplotype: HLA-A*32/68, HLA-B*35/38, HLA-C*04/07, HLA-DQA1*03/04, HLA-DQB1*03/03, HLA-DRB1*04/12 corresponding to KIRs 2DL1 (HLA-C*04 included in C1 epitope), 2DL2 (HLA-C*07 included in C1 epitope), 3DL1 (HLA-A*32, HLA-B*38 included in Bw4 epitope) and 3DL2 (HLA-A*68 included in HLA-A3 epitope) (HLA ligand for 2DL5 remains undefined). (C) Bar graphs showing the percentage of NK degranulation shown by CD107 positive expression in 2DL1+, 3DL1+ or NKG2A+ NK-cells when co-cultured with WT, SKO-B2M and DKO cells unstimulated or 2 days IFN-$\gamma$ 100 ng/mL pre-stimulation from 3 different donors.

Bars represent mean±SEM from 3 independent experiments.

*P-values: C (compared to respective No ST cell line per donor) <0.0001.

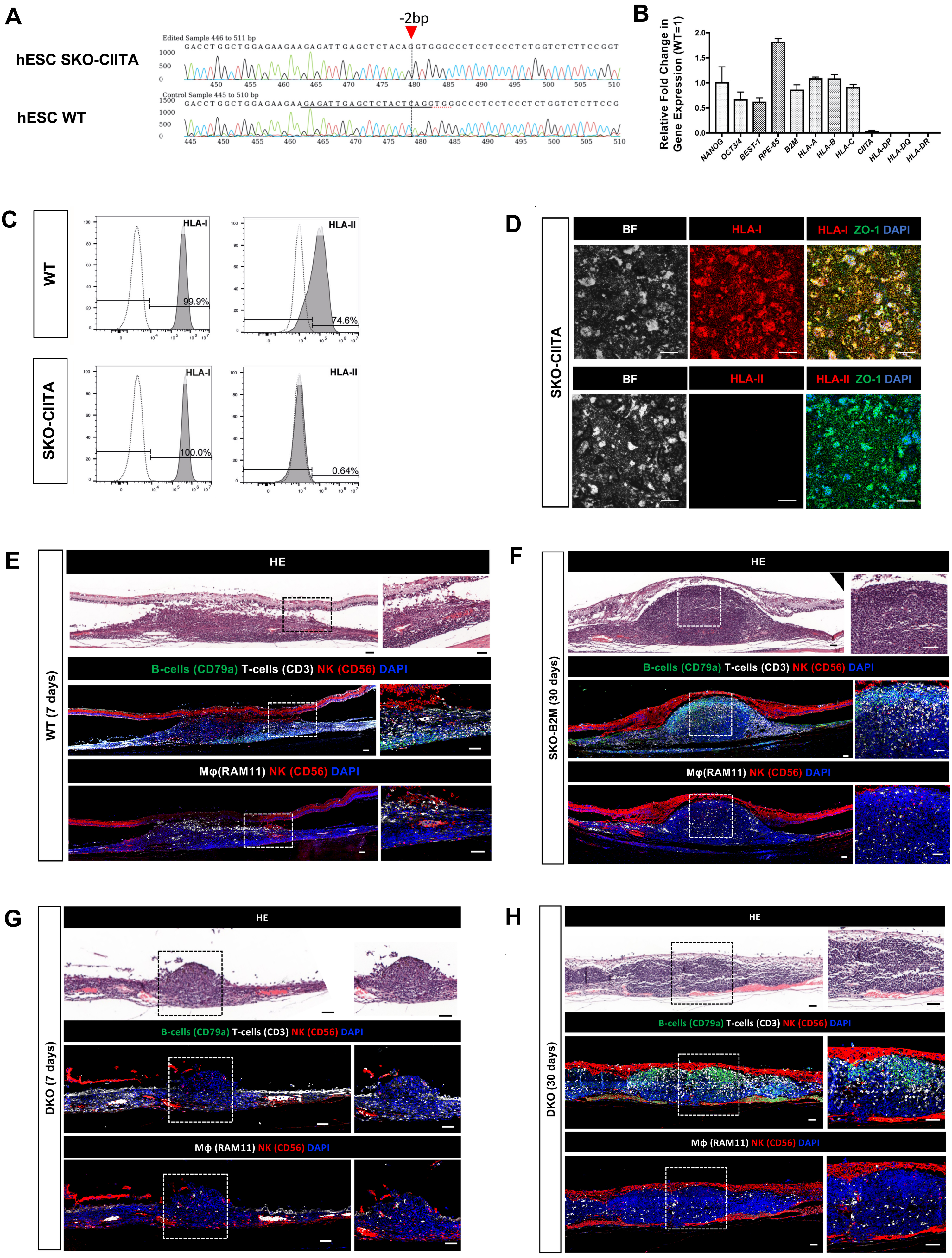
A
-2bp
hESC SKO-CIITA
hESC WT
Edited Sample 446 to 511 bp
Control Sample 445 to 510 bp
B
Relative Fold Change in Gene Expression (WT=1)
NANOG
OCT3/4
BEST-1
RPE-65
B2M
HLA-A
HLA-B
HLA-C
CIITA
HLA-DP
HLA-DQ
HLA-DR
C
WT
SKO-CIITA
HLA-I
HLA-II
99.9%
74.6%
100.0%
0.64%
D
SKO-CIITA
BF
HLA-I
HLA-I ZO-1 DAPI
HLA-II
HLA-II ZO-1 DAPI
E
WT (7 days)
HE
B-cells (CD79a) T-cells (CD3) NK (CD56) DAPI
Mφ(RAM11) NK (CD56) DAPI
F
SKO-B2M (30 days)
G
DKO (7 days)
H
DKO (30 days)

**FIGURE S5. Characterization of SKO-CIITA hESC-RPE cells and assessment of WT, SKO-B2M and DKO hESC-RPE upon subretinal injections in the xenograft model. Related to Figure 5.**

(A) Indel analysis obtained by Sanger sequencing of hESC SKO-CIITA. (B) Representative flow cytometry histogram showing the percentage of WT and SKO-CIITA cells expressing extracellular HLA-I and HLA-II. Dotted line histogram shows HLA-I FMO (negative control used for gating). (C) Immunofluorescence images of WT and SKO-CIITA cells showing HLA-I, HLA-II and ZO-1 expression. (D) Gene expression analysis of pluripotent, RPE and HLA related genes in the targeted hESC-RPE. Values are normalized to *GAPDH* and displayed as relative to WT. (E) HE and immunofluorescence images 7 days after subretinal injection of WT cells showing the expression of rabbit $CD3^{+}$ T-cells, $CD56^{+}$ NK cells, $CD79a^{+}$ B-cells and $RAM11^{+}$ macrophages. Dashed square indicates the zoom-in shown on the right side. (F) HE and immunofluorescence images 30 days after subretinal injection of SKO-B2M cells showing the expression of rabbit $CD3^{+}$ T-cells, $CD56^{+}$ NK cells, $CD79a^{+}$ B-cells and $RAM11^{+}$ macrophages. Dashed square indicates the zoom-in shown on the right side. (G) HE and immunofluorescence images 7 days after subretinal injection of DKO cells showing the expression of rabbit $CD3^{+}$ T-cells, $CD56^{+}$ NK cells, $CD79a^{+}$ B-cells and $RAM11^{+}$ macrophages. Dashed square indicates the zoom-in shown on the right side. (H) HE and immunofluorescence images 30 days after subretinal injection of DKO cells showing the expression of rabbit $CD3^{+}$ T-cells, $CD56^{+}$ NK cells, $CD79a^{+}$ B-cells and $RAM11^{+}$ macrophages. Dashed squares indicate the zoom-ins images shown on the right side.

Bars represent mean±SEM from 3 independent experiments.

Scale bars: (D) = 100 μm; (E-H) = 50 μm.

**VIDEO S1. Related to Figure 4.**

Movie showing 72 consecutive SD-OCT scans 7 weeks after injection of WT hESC-RPE cells in the subretinal space of the rabbit eye. Green arrow indicates the SD-OCT scan plane.

**TABLE S1. sgRNA sequences for gene targets B2M and CIITA. Related to Experimental Procedures.**

| | |
|---|---|
| B2M-1 | 5'-GGCCGAGATGTCTCGCTCCG-3' |
| B2M-6 | 5'-GAGTAGCGCGAGCACAGCTA-3' |
| B2M-7 | 5'-CAGTAAGTCAACTTCAATGT-3' |
| CIITA-4 | 5'-AGGTGATGAAGAGACCAGGG-3' |
| CIITA-5 | 5'-GAGATTGAGCTCTACTCAGG-3' |
| CIITA-9 | 5'-CATCGCTGTTAAGAAGCTCC-3' |

**TABLE S2. Primers utilized for amplification of gDNA through PCR and for SURVEYOR Nuclease Assay. Related to Experimental Procedures.**

| | |
|---|---|
| B2M Forward primer 1 | 5'-GCACGCGTTTAATATAAGTGGAGGCG-3' |
| B2M Forward primer 2 | 5'-CTCCAGCCTGAAGTCCTAGAATGAGC-3' |
| B2M Forward primer 3 | 5'-GTGGCTTGTTGGGAAGGTGGAAG-3' |
| B2M Reverse primer 1 | 5'-TTTGGGACGAGCCTACCCGT-3' |
| B2M Reverse primer 3 | 5'-GCTGTGCATCAGTATCTCAGCAGGT-3' |
| CIITA Forward primer 2 | 5'-AATGTGCCAGCTCATGTGCCTG-3´ |
| CIITA Forward primer 3 | 5'- CTCTCTGCAGATGGGGATGATCTCC-3' |
| CIITA Reverse primer 2 | 5'- TTGATGGTGTCTGTGTCGGGTTCT-3´ |
| CIITA Reverse primer 4 | 5'- GCAGAAATGCGTATAGAAATGGAGGTGG-3' |

**TABLE S3. Sequence read number, mean coverage (sequencing depth) and Germline SNVs for hESC WT (HS980), hESC SKO-B2M and hESC DKO samples. Related to Figure 1.**

| Sample Name | Reads (paired-end) | Aligned reds (%) (both in pairs) | Coverage (mean) | Germline SNVs (SNPs & Indels) |
|---|---|---|---|---|
| hESC (HS980) | 839393930 | 832313573 (99.16%) | 39.27 | 4604767 (3794906 & 813807) |
| hESC SKO-B2M | 687569526 | 672844498 (97.86%) | 30.36 | 4754497 (3925443 & 833210) |
| hESC DKO | 737021865 | 701549978 (95.19%) | 31.04 | 4748188 (3887630 & 864627) |

**TABLE S4. Total homozygous deletions identified for hESC WT vs hESC SKO-B2M, and hESC SKO-B2M vs hESC DKO samples. Related to Figure 1.**

See attached Table S4.xlsx file.

**EXPERIMENTAL PROCEDURES, Related to Experimental Procedures.**

**Cell Culture**

Human embryonic stem cell line HS980 was derived from supernumerary *in vitro* fertilized human embryos and cultured under xeno-free and defined conditions according to the previously described method (Rodin et al., 2014a; Rodin et al., 2014b) and under the approval of DNR 2011/745-31/3. The cells were maintained by clonal propagation on hrLN-521 10 µg/mL (Biolamina) in NutriStem hESC XF medium (Biological Industries, 05-100-1A), in a 5% $CO_2$/5% $O_2$ incubator and passaged enzymatically at a 1:10 ratio every 5-6 days.

For passaging, confluent cultures were washed twice with PBS without $Ca^{2+}$ and $Mg^{2+}$ (DPBS, ThermoFisher Scientific, 14190-094) and incubated for 5 min at 37°C, 5% $CO_2$/5% $O_2$ with TrypLE Select (ThermoFisher Scientific, 12563-011). The enzyme was then carefully removed and the cells were collected in fresh pre-warmed NutriStem hESC XF medium (Biological Industries, 05-100-1A) by gentle pipetting to obtain a single cell suspension. The cells were centrifuged at 300g for 4 min, the pellet resuspended in fresh prewarmed NutriStem hESC XF medium (Biological Industries, 05-100-1A) and cells plated on a freshly hrLN-521 (10 µg/mL, Biolamina) coated dish. Two days after passage the medium was replaced with fresh prewarmed NutriStem hESC XF medium (Biological Industries, 05-100-1A) and changed daily.

**hESC-RPE 2D *In Vitro* Differentiation**

Pluripotent stem cells were plated at a cell density of $2.4 \times 10^4$ cells/$cm^2$ on 20 µg/mL hrLN-111 coated dishes (Biolamina) using NutriStem hESC XF medium (Biological Industries, 05-100-1A). Rho-kinase inhibitor (Millipore, Y-27632) at a concentration of 10 µM was added during the first 24h, while cells were kept at 37°C, 5% $CO_2$/5% $O_2$. After 24h, hESC medium was replaced with differentiation medium NutriStem hESC XF without bFGF and TGFβ (Biologicial Industries, 06-5100-01-1A) and cells were placed at 37°C, 5% $CO_2$. From day 6 after plating, 100 ng/mL of Activin A (R&D Systems, 338-AC-050) was added to the media. Cells were fed three times a week and kept for 5 weeks. Monolayers were then trypsinized using TrypLE Select (ThermoFisher Scientific, 12563-011) for 10 min at 37°C, 5% $CO_2$. The enzyme was carefully removed and the cells were collected in fresh pre-warmed NutriStem hESC XF medium without bFGF and TGFβ (Biologicial Industries, 06-5100-01-1A) by gentle pipetting to obtain a single cell suspension. The cells were centrifuged at 300g for 4 min, the pellet was resuspended, passed through a cell strainer (ø 40 µm, VWR, 732-2757) and cells were seeded on LN-coated dishes (hrLN-521 20 µg/mL, Biolamina) at a cell density of $7 \times 10^4$ cells/$cm^2$. Re-plated cells were fed three times a week during the subsequent four weeks with the same differentiation medium referred above.

**Promoter Exchange of pX459**

The EF-1α promoter has been shown to be more stable in embryonic stem cells, which would ensure optimal expression of Cas9 and GFP or puromycin-resistance, respectively, from the plasmid (Liu et al., 2009) Firstly, the CMV promoter of pX458 (addgene no. 48138) and pX459 (addgene #62988) was exchanged for EF-1α (Fig S1A and S1B) by amplifying the latter off of pEF-GFP (addgene #11154) using forward and reverse primers 5'-AATTCTGCAGACAAATGGCTCTAGAGGTACGGTACCCGTGAGGCTCCGGTGCCC-3' and 5'-CGTGGTCCTTATAGTCCATGGTGGCACCGGACCGGTTCACGACACCTGAAATGGAAGAAAA-3' (Thermo Fisher Sicentific). The amplified fragment was ligated into BshTI (Thermo Fisher Sicentific, FD1464) and KpnI (Thermo Fisher Sicentific, FD0524) digested and FastAP (Thermo Fisher Sicentific, EF0652) treated pX459 using Gibson Assembly Master Mix (NEB, E2611S). The new plasmid constructs, henceforth referred to as pX459-EF-1α, were transformed into TOP10 bacteria (Thermo Fisher Sicentific, C404003) and plated onto ampicillin-containing LB agar plates (ThermoFisher Scientific, Q60120). Single colonies were picked and expanded in LB broth (ThermoFisher Scientific, 10855021) supplemented with 100 µg/mL ampicillin (ThermoFisher Scientific, 11593027) overnight, and plasmid DNA was extracted using QIAprep Spin Miniprep Kit (QIAGEN, 27104) according to the manufacturer's protocol.

**gRNA Cloning into pX459-EF-1α**

Digesting pX459-EF-1α using BpiI (ThermoFisher Scientific, FD1014) allowed for directional insertion of each gRNA. Prior to ligation, gRNA oligonucleotides (Table S1) were annealed in 1X Rapid ligation buffer (ThermoFisher Scientific, K1423) using a thermocycler set at 95°C for 5 min followed by 5°C/min ramp down to 25°C. The oligonucleotides were PNK (ThermoFisher Scientific, IVGN2304) treated according to the manufacturer's protocol. Annealed and PNK treated gRNA oligonucleotides were ligated into pX459- EF-1α using T4 DNA ligase (ThermoFisher Scientific, K1423) and the mixture was transformed into STBL3 bacteria (ThermoFisher Scientific, C737303) and plated on agar plates containing 100 µg/mL ampicillin. Single colonies were picked and expanded in LB broth with ampicillin (100 µg/mL) overnight and plasmid DNA was extracted using QIAprep Spin Miniprep Kit (QIAGEN, 27104) according to the manufacturer's protocol.

**Guide Electroporation, Selection and Clonal Expansion**

HEK293T cells (ThermoFisher Scientific, 293T Cells) were cultured in DMEM (ThermoFisher Scientific, 41965-039) supplemented with 10% FBS (ThermoFisher Scientific, 10082147), 0.1

mM NEAA (ThermoFisher Scientific; 11140-035), 6 mM GlutaMAXX (ThermoFisher Scientific, 35050-038) and 1 mM Sodium Pyruvate (ThermoFisher Scientific; 11360039) and dissociated using TrypLE Select (Gibco, 12563011). One hundred thousand cells were electroporated with 1 µg pX458_EF-1α-Cas9_U6-sgRNA using the NEON™ Transfection System (Thermo Fisher Sicentific, MPK5000) according to the manufacturer's protocol. Transfection success was estimated through GFP intensity from the pX458_EF-1α-Cas9_U6-sgRNA. The transfected cells were plated on 6-well plates and were allowed to recover until confluent before harvesting the cells for gDNA extraction and mutation detection.

Human embryonic stem cells (HS980) were cultured in Nutristem hESC XF medium (Biological Industries, 05-100-1A) as described previously until they reached 70–80% confluency, at which time they were dissociated using TrypLE Select (Gibco, 12563011) and prepared for NEON™ electroporation (ThermoFisher Scientific, MPK5000) according to the manufacturer's protocol. One hundred thousand cells were electroporated with pX459-EF-1α-Cas9-U6–B2M and then plated into a well on a six-well plate previously pre-coated overnight with 10 µg/mL hrLN-521 (Biolamina) in Dulbecco's Phosphate Buffered Saline containing calcium and magnesium (Gibco, 14040091). Twenty-four hours post-electroporation cells were selected using 0.5 µg/mL Puromycin (ThermoFisher Scientific, A1113803) for 24 hours after which fresh media was changed and the cells were left to recover. Bulk targeting efficiencies were determined using ICE analysis provided by Synthego (https://ice.synthego.com/#/) and were 80% and 84% for B2M and CIITA gRNAs, respectively. Once the cells were 70–80% confluent they were dissociated, diluted and plated at a concentration of two cells per well in a 96-well plate previously coated with 15 µg/mL hrLN-521 (Biolamina, LN521) and 1.7 µg/mL E-cadherin (R&D Systems, 8505-EC-050) overnight. Rho-kinase inhibitor (Millipore, Y-27632) at a concentration of 10µM was added during the first 24 hours. Single-cell clones were cultured in this format until confluent and were then dissociated and plated into two 48-wells per clones for further expansion. Cells in one out of the two wells were then used for genomic DNA extraction and Sanger sequencing of the region surrounding the edit using *B2M* primers F2 and R1 (Table S2) for PCR amplification. For hESC SKO-B2M, 20 single-cell clones were sequenced at the target site and all contained various indels. This procedure was repeated using the hESC SKO-B2M line starting from the editing step by electroporating one hundred thousand hESC SKO-B2M α-Cas9-U6–CIITA to generate hESC DKO. Amplification of the region surrounding the sgRNA-CIITA-5 edit site was done using *CIITA* primers F2 and R2 (Table S2). For hESC

DKO$^{-}$, 42 single-cell clones were sequenced at the *CIITA* target site out of which 37 contained indels and five remained unedited.

**Mutation Detection using SURVEYOR Nuclease Assay**

Genomic DNA extraction was performed using QuickExtractTM DNA extraction solution (Lucigen, QE09050) and 1 µL of the extracted DNA was utilized for PCR amplification using Herculase II Fusion Enzyme (Agilent, 600677) and primers F1 and R1 for sgRNAs 1 and 2 located in exon 1 and primers F3 and R3 for sgRNA-3 located in exon 2 in the B2M locus, (Table S2). Primers F2 and R2 amplified a region around sgRNAs 5 and 9 and primers F3 and R4 amplified a region surrounding sgRNA 4 in the CIITA locus (Table S2). Mutation detection was performed using SURVEYOR Nuclease Assay (IDT, 706020) according to the manufacturer's protocol. The mismatch analysis was carried out according to the published protocol by Ran et al. (2013). Indel percentage was calculated according to $100*1-\surd((1-((b+c))/((a+b+c))))$, where $a$ symbolizes the integrated intensity of the unedited DNA fragment, and $b$ and $c$ represent the intensities for each cleaved fragment (Ran et al., 2013).

**Detection of Cas9 in Cells using Fluorescence Activated Cell Sorting (FACS)**

HS980 hESCs were cultured and electroporated at nine, four and one days before analysis through FACS. Cells were selected for 24h with 0.5µg/ml puromycin (ThermoFisher Scientific, A1113803), except for day 1 samples that were not selected but collected for analysis. Cells were dissociated using TrypLE Select (Gibco, 12563011), centrifuged at 300g for 4 min and resuspended in DPBS (ThermoFisher Scientific, 14190-094). Samples were incubated with LIVE/DEAD fixable Violet Dead Cell Stain (Invitrogen, L34964) according to the manufacturer's protocol. Following LIVE/DEAD stain, fixation and permeabilization was carried out according to Cytofix/Cytoperm$^{TM}$ Fixation/Permeabilization Kit (BD Biosciences, 554714) for intracellular staining. Briefly, LIVE/DEAD stained samples were incubated with Fixation/Permeabilization solution for 20 minutes at 4°C and washed twice with 1 x BD Perm/Wash buffer. Samples were stained for intracellular Cas9 with an Alexa Fluor 647 conjugated Cas9 antibody (1:50, Cell Signaling Technologies, 48796) for 30 minutes at 4°C.Pellets were washed twice with 1 x BD Perm/Wash buffer and finally resuspended in 2% FBS (ThermoFisher Scientific, 10082147) and 1mM EDTA (Sigma, E7889) diluted in DPBS (ThermoFisher Scientific, 14190-094). Non-electroporated cells per time-point were used to set the respective negative gates. Stained cells were analyzed using a Cytoflex flow cytometer (Beckman Coulter). Analysis of the data was carried out using FlowJo v.10 software (Tree Star). Biological triplicates were performed for every condition. Results are presented as mean±SEM (standard error of the mean).

**Pre-processing of Whole-Genome Sequencing Reads**

Whole genome DNA sequencing reads (paired-end; 150bp) of human embryonic stem cell line HS980, hESC SKO-B2M and hESC DKO cells were aligned to the human reference genome (NCBI reference genome GRCh37 based "human_g1k_v37") using the Burrows-Wheeler Aligner (BWA-0.7.12) (Li and Durbin, 2010). Sequencing data is available upon request. Duplicate reads in reference genome aligned BAM files were marked using Picard 2.9.0 "MarkDuplicates" utility. Next, we followed the "GATK Best Practice" guidelines (DePristo et al., 2011; Van der Auwera et al., 2013) using GATK 3.7 toolkit and performed local realignments and base quality recalibration with default parameters. GATK resource bundle "b37" that include data sets from HapMap, Omni, Mills Indels, 1000 Genome Indels and dbSNP v138 databases were used in this analysis. Further, germline SNVs and indels were identified using HaplotypeCaller with default parameters.

**Identification of Somatic SNVs and Copy Number Deletions**

We considered non-redundant whole exonic intervals (GRCh37 gene annotation) and predicted off-target sites for SNV analysis. Separate list of potential off-targets sites for hESC SKO-B2M and hESC DKO experiments were created using Cas-OFFinder (Bae et al., 2014) (up to nine mismatches allowed and up to 2bp bulge formation of either RNA or DNA) and E-CRISP (Heigwer et al., 2014) (not allowed for any 5' mismatches to be ignored and tolerated edit distance to the target sequence set to three) online prediction tools. We used 'NGG' as PAM motif sequence and 5'-GGCCGAGATGTCTCGCTCCG-3' and 5'-GAGATTGAGCTCTACTCAGG-3', as B2M and CIITA sgRNA sequences, respectively, for off-target sites prediction. All exonic intervals and predicted off-target sites (75 bases flanking both ends) were searched for SNVs using GATK 3.7 MuTect2 utility (Cibulskis et al., 2013) with default parameters. SNVs with allele frequency (AF) ≥ 0.25 and read depth (DP) > 10 were considered for further analysis. Somatic copy number deletions were then considered since in principle a copy number deletion refers to loss of large genomic segment and a predicted off-target site could be completely missed if it is within copy number deleted regions. To ensure that such deletions have not eliminated a potential off-target site we performed copy number deletion and loss of heterozygosity (LOH) analysis using VarScan 2.3.9 (Koboldt et al., 2012) with default parameters. Briefly, we performed pair-wise somatic SNVs, copy number deletion and LOH analysis between hESC WT (HS980) vs hESC SKO-B2M, and hESC SKO-B2M vs hESC DKO samples. Heterozygous copy number deletions (fold change ≥ 1.5) overlapping with LOH regions were considered for further downstream analysis. Overlapping study of predicted off-targets with copy number deletions was performed using UCSC Galaxy bed-tools "intersect intervals" utility.

**Quantitative Real-time PCR**

Total RNA was isolated using the RNeasy Plus Mini Kit (Qiagen, 74106) and treated with RNase-free DNase (Qiagen, 79254) and RNaseH (ThermoFisher Scientific,18021071) according to manufacturer’s protocol. cDNA was synthesized using 1 µg of total RNA in a 20 µL reaction mixture containing random hexamers (ThermoFisher Scientific, N8080127) and Superscript III reverse transcriptase (ThermoFisher Scientific, 18080085), according to the manufacturer’s instructions.

Taq-polymerase (ThermoFisher Scientific, 4304437) together with Taqman probes (ThermoFisher Scientific) for *GAPDH* (4333764F), *NANOG* (Hs02387400_g1), *POU5F1/OCT4* (Hs03005111_g1), *BEST1* (Hs00188249_m1), *RPE65* (Hs01071462_m1), *HLA-A* (HS01058806_g1), *HLA-B* (HS00818803_g1), *HLA-C* (HS00740298_g1), *HLA-E* (HS03045171_m1), *HLA-DP* (HS00410276_m1), *HLA-DQ* (HS03007426_mH), *HLA-DR* (HS00219575_m1), *B2M* (HS00187842_m1) and *CIITA* (HS00172106_m1) were used. Samples were subjected to real-time PCR amplification protocol on StepOneTM real-time PCR System (Applied Biosystems). Biological triplicates were performed for every condition and technical triplicates were carried out for each sample. Results are presented as mean±SEM (standard error of the mean).

**Western Blot**

Total protein was extracted from cells by dissolving the collected cell pellet in cold RIPA buffer (Sigma, R0278) and incubating on ice for 5 min before centrifugation at 2000g for 5 min. The supernatant was transferred to a clean tube and protein concentration was measured using the Pierce™ BCA protein assay (Thermo Fisher Sicentific, 23227) according to the manufacturer’s protocol. A total of 223 µg protein was loaded on a 4-20% TGX gel (Bio-Rad, 4568094) after being incubated in 1X Laemmli sample buffer (Bio-Rad, 161-0737), supplemented with 2-mercaptoethanol (ThermoFisher Scientific, 31350-010), at 95°C for 5 min. Following electrophoresis (120V for 70-80 min at room temperature), blotting (100V for 60 min at room temperature) was carried out using an 0.2 µM PVDF membrane (Thermo Fisher Sicentific; LC2002). Primary antibodies for B2M (1:200, Santa Cruz Biotech, B2M-02), HLA-I (1:500, Abcam, ab70328) and GAPDH (1:10000, Abcam, ab8245) were diluted in 3% BSA (Sigma, A9418) in 0.05% Tween-20 (Sigma, T8787) in DPBS (ThermoFisher Scientific, 14190-094). Primary antibody incubation was done on a rotator at 4°C overnight. The membrane was washed 3 x 10 min using 0.05% Tween-20 (Sigma, T8787) in DPBS (ThermoFisher Scientific, 14190-094), which was followed by secondary antibody incubation at room temperature for 1 hour using Alexa-680 (1:10000, ThermoFisher Scientific, A10038). The membrane was then washed 3 x 10 min with 0.05% Tween-20 (Sigma, T8787) in DPBS (ThermoFisher Scientific, 14190-094) before the membrane was imaged using LI-COR

Odyssey infrared imager (LI-COR).

**Flow Cytometry**

After one month in culture, hESC-RPE were dissociated into single cells using TrypLE Select (ThermoFisher Scientific, 12563-011) as described above. Samples were stained using the following conjugated antibodies: mouse anti-human HLA-ABC-FITC (1:20, BD Biosciences, 555552), mouse anti-human HLA-DR-V500 (1:66, BD Biosciences, 561224), mouse anti-human PD-L1-PE (1:30, Biolegend 329706, clone [29E.2A3]), mouse anti-human PD-L2-APC (1:20, BD Biosciences, 557925, clone [MIH18]), mouse anti-human CD80-PE (1:20, BD Biosciences, 557227, clone [L307.4]), mouse anti-human CD86-FITC (1:20, BD Biosciences, 555657, clone [2331, FUN-1]), mouse anti-human HLA-ABC-BV421 (1:100, BD Biosciences, 565332, clone [G46-2.6]), mouse anti-human HLA-A2-BV510 (1:100, BioLegend 343319, clone [BB7.2]), mouse anti-human HLA-C-PE (1:20, BD Biosciences, 566372, clone [DT-9]), mouse anti-human MICAB-AF488 (1:100, BioLegend 320912, clone [6D4]), mouse anti-human CD112-PeCy7 (1:100, BioLegend 337413, clone [TX31]), mouse anti-human CD155-BV605 (1:100, BD Biosciences, 745215, clone [TX24]), mouse anti-human PCNA-AF647 (1:100, BioLegend 307912, clone [PC10]), mouse anti-human CD158b/2DL2/L3/S5-BV711 (1:50, BD Biosciences, 743454, clone [CH-L]), mouse anti-human CD158F/2DL5-APC (1:100, MACS, 130-098-567), mouse anti-human CD158E1/3DL1-APC-Fire750 (1:50, Biolegend, 312722, clone [DX9]), mouse anti-human 3DL2-PE (1:100, R&D, FAB2878P), mouse anti-human CD158a,h/2DL1-PC7 (Beckman Coulter), human anti-human CD159a/NKG2A-FITC (1:400, MACS, 130-105-646), mouse anti-human CD107a-BV421 (1:100, Biolegend, 328626, clone [H4A3]), mouse anti-human CD47 (Human Cell Surface Marker Screening Panel, BD Biosciences, 560747, clone [B6H12]), mouse anti-human CD55 (Human Cell Surface Marker Screening Panel, BD Biosciences, 560747, clone [IA10]), mouse anti-human CD59 (Human Cell Surface Marker Screening Panel, BD Biosciences, 560747, clone [p282 (H19)]) diluted in 2% FBS (ThermoFisher Scientific, 10082147 and 1mM EDTA (Sigma, E7889) DPBS (ThermoFisher Scientific, 14190-094) or in Brilliant Buffer (BD Biosciences, 563794). Cells were incubated with the conjugated antibodies at 4°C for 30 min and washed twice with 2% FBS (ThermoFisher Scientific, 10082147) and 1mM EDTA (Sigma, E7889) diluted in DPBS (ThermoFisher Scientific, 14190-094). For CD47, CD55 and CD59 antibodies, an extra 30 min incubation step with secondary antibody Alexa Fluor 647 donkey anti-mouse IgG (Human Cell Surface Marker Screening Panel, BD Biosciences, 560747) was performed. Fluorescence minus one (FMO) or isotype mIgG controls were included for each condition to gate negative and positive cells. 7-AAD-PeCy5 (1:200, BD Biosciences, 51-68981E) or Zombie-APC-H7 NIR (1:500, Biolegend, 423105) was added to the cells for Live/Dead stain according manufacturer instructions. Respective Geometric

Mean Fluorescence Intensity (GMFI) values of the FMO controls were subtracted to obtain the final GMFI of each fluorophore. Stained cells were analyzed using a Cytoflex flow cytometer (Beckman Coulter). Analysis of the data was carried out using FlowJo v.10 software (Tree Star). Biological triplicates were performed for every condition. Results are presented as mean±SEM (standard error of the mean).

**Immunocytofluorescence**

Protein expression of mature hESC-RPE monolayers was assessed with immunofluorescence. Cells were fixed with 4% methanol-free formaldehyde (VWR, FFCHFF22023000) at room temperature for 10 min, followed by permeabilization with 0.3% Triton X-100 (Sigma, T9284) in DPBS (ThermoFisher Scientific, 14190094) for 10 min and blocking with 4% fetal bovine serum (FBS, ThermoFisher Scientific, 10082147) and 0.1% Tween-20 (Sigma, P9416) in DPBS for 1 hour both at room temperature. Primary antibodies were diluted to the specified concentrations in blocking solution: mouse anti-human B2M (1:100, Santa Cruz, sc-41410 [B2M-02]), mouse anti-human HLA-ABC (1:100, Abcam, ab70328 [EMR8-5]), mouse anti-human HLA-DP,DQ,DR (1:100, DAKO, M0775 [CR3/43]), rabbit anti-human Nanog (1:100, Reprocell, RCAB0003P) and rabbit anti-human ZO-1 (1:100, ThermoFisher Scientific, 40-2200). The primary antibodies were incubated overnight at 4°C followed by 2 hours incubation at room temperature with secondary antibodies: Alexa Fluor 555 donkey anti-mouse IgG (1:1000, ThermoFisher Scientific, A31570) and Alexa Fluor 488 donkey anti-rabbit IgG (1:1000, ThermoFisher Scientific, A21206) diluted 1:1000 in blocking solution. Nuclei were stained with Hoechst 33342 (1:1000, ThermoFisher Scientific, Invitrogen H3570). Images were acquired with Olympus IX81 fluorescence microscope (Carl Zeiss Meditec, 10x objective). Post-acquisition analysis of the pictures was performed using ImageJ/Fiji software.

**Histology and Tissue Immunostaining**

Immediately after sacrifice by intravenous injection of 100 mg/kg pentobarbital (Allfatal vet. 100 mg/ml, Omnidea), the eyes were enucleated and the bleb injection area marked with green Tissue Marking Dye (TMD; Histolab Products AB, 02199). An intravitreal injection of 100 µL fixing solution (FS) consisting of 4% buffered-formaldehyde (Histolab Products AB, 02175) was performed. FS remained for 24-48 hours followed by embedding in paraffin. 4 µm serial sections were made through the TMD-labeled area and every 4 sections were stained with hematoxylin-eosin (VWR, 1051740500).

For immunostaining, a Bond III system from Leica Biosystems/Leica Triolab AB was used. Following the manufacturer’s instructions, slides were deparaffinized in xylene for 5 min, washed with Bond Wash Solution (Leica Biosystems, AR9590), pretreated with EDTA-buffer

pH9 (Leica Biosystems, AR9640) at 100°C for 20 min, washed with Bond Wash Solution (Leica Biosystems, AR9590) and blocked with perxideblock 3% (Leica Biosystems, DS9263) for 15 min at room temperature. Slides were washed with Bond Wash Solution (Leica Biosystems, AR9590) and primary antibodies diluted in Bond Antibody Diluent Solution (Leica Biosystems, AR9352) were incubated for 60 min at room temperature: rabbit anti-human nuclear mitotic apparatus protein (NuMA) (1:200, Abcam ab84680), mouse anti-human HLA-ABC (1:100, Abcam, ab70328 [EMR8-5]), mouse anti-human HLA-DP,DQ,DR (1:100, Dako, M0775 [CR3/43]), rat anti-rabbit CD3 (1:100, Abcam, ab11089, [CD3-12]), rabbit anti-human CD56 (1:200, Cellmarque, 156R-95) mouse anti-rabbit RAM11 (1:50, DAKO, M0633), mouse anti-rabbit CD79a (1:100, ThermoFisher Scientific, MA5-13212 [HM47/A9]). After a washing step with Bond Wash Solution (Leica Biosystems, AR9590), secondary antibodies (1:200, Alexa Fluor 546 goat anti-rabbit IgG (A11035), Alexa Fluor 647 donkey anti-mouse IgG (A31571), Alexa Fluor 546 goat anti-rat IgG (A11081), Alexa Fluor 647 goat anti-rabbit IgG (A21245); all from ThermoFisher Scientific) diluted 1:2000 in Bond Antibody Diluent Solution (Leica Biosystems, AR9352) were incubated for 15 min at room temperature, washed with Bond Wash Solution (Leica Biosystems, AR9590) and incubated with Hoechst 33342 solution in PBS (5g/L, Sigma, 33258) for 5 min at room temperature. After washing steps with Bond Wash Solution (Leica Biosystems, AR9590) and deionized water, sections were mounted with DAKO Fluorescence Mounting Media (DAKO, S3023) on a 24x50 mm coverslip. Images were taken with Zeiss LSM710 point scanning confocal microscope (Carl Zeiss Meditec, 20x objective). Post-acquisition analysis of the pictures was performed using ImageJ/Fiji software.

**hESC-RPE and T-cell Co-culture**

Firstly, day 30 (after replating) unstimulated or 2 days IFN-$\gamma$ pre-stimulated (100 ng/mL, Peprotech, 300-02) hESC-RPE cells were trypsinized as described above, irradiated (30 Gy) and plated at a cell density range of $1x10^3$ (1:500) - $5.5x10^5$ (1:1) cells/$cm^2$ (depending on the respective experiment) on hrLN-521 coated dishes (20 $\mu$g/mL, Biolamina) using complete RPMI medium (Hyclone, SH3025501) with 10% AB serum (Sigma, H3667) and 100U/mL penicillin and 100 $\mu$g/mL streptomycin (Hyclone, SV30010). hESC-RPE cells were left for at least 3 hours to let attach to the plate. Secondly, human PBMCs were isolated from buffy coats of healthy donors by Lymphoprep (Axis-Shield PoC AS, 1114547) density gradient centrifugation. After washing with PBS, cell numbers were assessed by counting with Sysmex (Sysmex Sverige, KX-21N). PBMCs were then either stained with CellTrace CFSE Cell Proliferation Kit (2.5 $\mu$g/mL, ThermoFisher Scientific, C34554) or divided into two tubes for CD4+ and CD8+ isolation with commercially available CD4 and CD8-negative selection beads from MACS, Miltenyi Biotec (130-096-533 and 130-096-495, respectively) in

accordance with the instructions of the manufacturer. Purities were of above 90% for either CD4+ or CD8+ selected cells in the two donors used for the experiments (data not shown). Finally, 1 million of the labelled or unlabelled PBMCs, or isolated CD4+ or CD8+ T-cells were plated per well in a 24-well plate on top of the attached unstimulated or pre-stimulated hESC-RPE. IL-2 (1 ng or 100U, BD Biosciences, 554603), CD28 (1.25 μg/mL, Biolegend, 302902 [CD28.2]) or OKT-3 (25 ng/mL, Biolegend, 317315) molecules were added the wells if required. For MLR, PBMCs were isolated from different donors as described above. Irradiated (30Gy) and non-irradiated CFSE-labelled PBMCs were subsequently co-cultured with the same numbers (1 million each) and the corresponding stimulatory molecules were added if required. Co-cultures were maintained for 5 days at 37°C for further analysis.

**Enzyme-Linked Immunosorbent Assay (ELISA)**

Supernatants from hESC-RPE and T-cell co-cultures (either with CD8+ or CD4+ positive isolated human PBMCs) co-cultures were collected 5 days after the cells were plated. IFN-γ secretion levels were measured in triplicates for each condition with commercially available human IFN-γ ELISA Kit (Mabtech, 3420-1HP-2) in accordance with the instructions of the manufacturers. The optical density readings were measured using SpectraMax i3x Reader (MolecularDevices). Results are presented as mean±SEM (standard error of the mean).

**Isolation of Human NK Cells from Healthy Donors**

Human PBMCs were isolated as described above and consecutively NK cells were negatively separated with a CD56 isolation kit (Miltenyi Biotec, 130-050-401) using autoMACs Pro Separator (Miltenyi Biotec) with the "depletes" program. Purities were of above 90% for CD56+ CD3- selected cells in the three donors used for the experiments (data not shown). Final cell numbers were assessed by Türks solution (Histolab Products AB, HL22200.0100) and cells were seeded out at a concentration of $1x10^6$ cells/mL in stem cell growth medium (CellGro, CellGenix, 20802-0500) with 20% heat inactivated FBS (ThermoFisher Scientific, 10500064) and activated over night with 500 U/mL IL-2 (R&D Systems, 202-GMP-01M).

**Cytotoxicity Assay**

NK mediated cytotoxicity was measured in a $^{51}Cr$-release assay with overnight IL-2 activated hNK cells (effector cells) against unstimulated or 2 days IFN-γ pre-stimulated (100 ng/mL, Peprotech, 300-02) hESC-RPE. hESC-RPE (target cells) were labeled with 70 μCi $^{51}Cr$ (PerkinElmer, Waltham, MA) for 1 hour at 37°C, and NK cells were then mixed with the labeled target cells at different effector:target ratios (10:1; 3:1; 1:1; 0.3:1) in a 96-well plate

and incubated for 4 hours at 37°C. After, supernatants (25 μL) were transferred into LumaPlate-96 Deep-Well and analyzed using a MicroBeta$^2$ LumiJET Microplate counter (PerkinElmer, Waltham, MA). *Percentage of specific lysis per sample type = [(experimental - spontaneous release) / (maximum load - spontaneous release) x 100].* Results are presented as mean±SEM (standard error of the mean).

**Degranulation Assay**

NK in vitro response was measured in a 4h degranulation assay with overnight IL-2 activated hNK cells (effector cells) against unstimulated or 2 days IFN-γ pre-stimulated (100 ng/mL, Peprotech, 300-02) hESC-RPE (target cells). NK cells were mixed with target cells at an effector:target ratio of 3:1 in a 96-well plate in the presence of anti-CD107 mAb and incubated at 37°C. After 1 hour, monensin (GolgiStop, BD Biosciences, 554724) was added to block protein transport, and cells were incubated for additional 3h at 37°C. Cells were stained for the indicated surface molecules and subsequently for intracellular cytokines (IFN-γ) using BD Cytofix/Cytoperm kit (BD Biosciences, 554714), according to manufacturer recommendations. Flow Cytometry was performed as described above and cells were acquired on a BD Symphony (Becton Dickinson). Analysis of the data was carried out using FlowJo v.10 software (Tree Star). Biological triplicates were performed for every condition. Results are presented as mean±SEM (standard error of the mean).

**Rabbit Serum Collection**

5-10 mL of blood was extracted from the rabbits prior and after subretinal injections at different time points (d7, d14, d30 and d90) in serum collection tubes (Vacuette Z Serum Sep Clot Activator, Greiner Bio-One, 455010). The tubes were rested in a standing position for about 15-20 min until blood was clotted, and then centrifuged at 20°C, 1500g for 10 min. Serum (supernatant) was quickly removed and stored at -80°C in 1mL aliquots for further analysis.

**Antibody-mediated Assay**

100,000 cultured WT hESC-RPE cells were pelleted and mixed with 100 μL of serum for 30 min at room temperature. After two washes with 2% FBS (ThermoFisher Scientific, 10082147) and 1mM EDTA (Sigma, E7889) in DPBS (ThermoFisher Scientific, 14190-094) at 300g for 5 min, secondary antibody donkey anti-rabbit A488 (ThermoFisher Scientific, A21206/R37118) diluted 1:1000 was added for 20 min at 4°C and washed two extra times (300g, 5 min). Finally, 7-AAD-PeCy5 (1:200, BD Biosciences, 51-68981E) Live/Dead stain diluted in 2% FBS (ThermoFisher Scientific, 10082147) and 1mM EDTA (Sigma, E7889) in DPBS (ThermoFisher Scientific, 14190-094) was added to the cells. Stained cells were

analyzed using a Cytoflex flow cytometer (Beckman Coulter). Analysis of the data was carried out using FlowJo v.10 software (Tree Star). Results are presented as mean±SEM (standard error of the mean).

**Animals**

After approval by the Northern Stockholm Animal Experimental Ethics Committee (DNR N25/14), female New Zealand white albino rabbits (provided by Lidköpings rabbit farm, Lidköping, Sweden) aged 5 months and weighing 3.5 to 4.0 kg were used in this study. All experiments were conducted in accordance with the Statement for the Use of Animals in Ophthalmic and Vision Research.

**Subretinal Transplantation**

hESC-RPE monolayers were washed with DPBS (ThermoFisher Scientific, 14190-094), incubated with TrypLE (ThermoFisher Scientific, 12563-011) and dissociated to single cell suspension as described above. Cells were counted in a Neubauer hemocytometer (VWR, 631-0925) chamber using 0.4% trypan blue (ThermoFisher Scientific, 15250061), centrifuged at 300g for 4 min, and the cell pellet was resuspended in freshly filter-sterilized DPBS (ThermoFisher Scientific, 14190-094) to a final concentration of 1000 cells/µL. The cell suspension was then aseptically aliquoted into 600 µL units and kept on ice until surgery.

Animals were put under general anesthesia by intramuscular administration of 35 mg/kg ketamine (Ketaminol 100 mg/mL, Intervet, 511519) and 5 mg/kg xylazine (Rompun vet. 20 mg/mL, Bayer Animal Health, 22545), and the pupils were dilated with a mix of 0.75% cyclopentolate / 2.5% phenylephrine (APL, 321968). Microsurgeries were performed on both eyes using a 2-port 25G transvitreal pars plana technique (Alcon Nordic A/S, 8065751448). 25G trocars were inserted 1 mm from the limbus and an infusion cannula was connected to the lower temporal trocar. The cell suspension was drawn into a 1 mL syringe connected to an extension tube and a 38G polytip cannula (MedOne Surgical Inc, 3219 and 3223). Without infusion or prior vitrectomy the cannula was inserted through the upper temporal trocar. After proper tip positioning, ascertained by a focal whitening of the retina, 50 µL of cell suspension (equivalent to 50,000 cells) was injected slowly subretinally approximately 6 mm below the inferior margin of the optic nerve head, forming a uniform bleb that was clearly visible under the operating microscope. To minimize reflux, the tip was maintained within the bleb during the injection. After instrument removal light pressure was applied to the self-sealing suture-less sclerotomies. 2 mg (100 µL) of intravitreal triamcinolone (Triescence 40 mg/mL, Alcon Nordic A/S, 412915) was administered a day prior to the surgery, and no post-

surgical antibiotics were given. For the TCA cohort, intravitreal triamcinolone was re-administered every 3 months, if required.

**Multimodal Real-time Imaging and Choroidal and Subretinal Thickness Measurements**

Multimodal real-time imaging was performed as described before (Bartuma et al., 2015). Briefly, anesthetized animals were placed in an adjustable mount (Spectralis HRA + OCT device, Heidelberg Engineering with the Heidelberg Eye Explorer software) to obtain horizontal cross-sectional b-scans of treated animals. *En-face* fundus images were obtained by multicolor cSLO. For thickness measurements, SD-OCT scans were randomly acquired through the upper, middle and lower segments of the subretinal transplantation area, and the scan with the largest subretinal infiltrate and simultaneous choroidal thickening was chosen for analysis. The height of the subretinal infiltrate (from the Bruch's membrane to the outer border of the neurosensory retina) was measured at the thickest point using ImageJ/Fiji. To obtain the value for choroidal thickening, the total thickness of the choroid was measured at the same position and subtracted from the choroidal thickness outside the transplantation area (naive choroid). Similarly, for evaluation of rejection rates, SD-OCT scans were randomly acquired as for choroidal measurement, and if any subretinal infiltrate and choroidal thickening was observed in any of the scans the transplant was scored as rejected.

**Statistical Analysis**

For statistical analyses, Chi-squared test was used to compare differences in rejection rates in the three subretinally transplanted groups with WT, SKO-B2M, SKO-CIITA and DKO cells. ANOVA (one-way or two-way) and posthoc multiple comparisons using Tukey test were performed to assess the *in vitro* differences of the different knock out lines upon co-culture with immune cells, and to assess anti-human antibody presence upon subretinal injection in the xenograft model.

**REFERENCES**


Bae, S., Park, J., and Kim, J.S. (2014). Cas-OFFinder: a fast and versatile algorithm that searches for potential off-target sites of Cas9 RNA-guided endonucleases. Bioinformatics *30*, 1473-1475.

Bartuma, H., Petrus-Reurer, S., Aronsson, M., Westman, S., Andre, H., and Kvanta, A. (2015). In Vivo Imaging of Subretinal Bleb-Induced Outer Retinal Degeneration in the Rabbit. Invest Ophthalmol Vis Sci *56*, 2423-2430.

Cibulskis, K., Lawrence, M.S., Carter, S.L., Sivachenko, A., Jaffe, D., Sougnez, C., Gabriel, S., Meyerson, M., Lander, E.S., and Getz, G. (2013). Sensitive detection of somatic point mutations in impure and heterogeneous cancer samples. Nat Biotechnol *31*, 213-219.

DePristo, M.A., Banks, E., Poplin, R., Garimella, K.V., Maguire, J.R., Hartl, C., Philippakis, A.A., del Angel, G., Rivas, M.A., Hanna, M.*, et al.* (2011). A framework for variation discovery and genotyping using next-generation DNA sequencing data. Nat Genet *43*, 491-498.

Heigwer, F., Kerr, G., and Boutros, M. (2014). E-CRISP: fast CRISPR target site identification. Nat Methods *11*, 122-123.

Koboldt, D.C., Zhang, Q., Larson, D.E., Shen, D., McLellan, M.D., Lin, L., Miller, C.A., Mardis, E.R., Ding, L., and Wilson, R.K. (2012). VarScan 2: somatic mutation and copy number alteration discovery in cancer by exome sequencing. Genome Res *22*, 568-576.

Li, H., and Durbin, R. (2010). Fast and accurate long-read alignment with Burrows-Wheeler transform. Bioinformatics *26*, 589-595.

Liu, J., Jones, K.L., Sumer, H., and Verma, P.J. (2009). Stable transgene expression in human embryonic stem cells after simple chemical transfection. Mol Reprod Dev *76*, 580-586.

Ran, F.A., Hsu, P.D., Wright, J., Agarwala, V., Scott, D.A., and Zhang, F. (2013). Genome engineering using the CRISPR-Cas9 system. Nat Protoc *8*, 2281-2308.

Rodin, S., Antonsson, L., Hovatta, O., and Tryggvason, K. (2014a). Monolayer culturing and cloning of human pluripotent stem cells on laminin-521-based matrices under xeno-free and chemically defined conditions. Nat Protoc *9*, 2354-2368.

Rodin, S., Antonsson, L., Niaudet, C., Simonson, O.E., Salmela, E., Hansson, E.M., Domogatskaya, A., Xiao, Z., Damdimopoulou, P., Sheikhi, M.*, et al.* (2014b). Clonal culturing of human embryonic stem cells on laminin-521/E-cadherin matrix in defined and xeno-free environment. Nat Commun *5*, 3195.

Van der Auwera, G.A., Carneiro, M.O., Hartl, C., Poplin, R., Del Angel, G., Levy-Moonshine, A., Jordan, T., Shakir, K., Roazen, D., Thibault, J., *et al.* (2013). From FastQ data to high confidence variant calls: the Genome Analysis Toolkit best practices pipeline. Curr Protoc Bioinformatics *43*, 11 10 11-33.
