# Supplementary material for: Generation of Retinal Pigment Epithelial Cells Derived from Human Embryonic Stem Cells Lacking Human Leukocyte Antigen Class I and II
Source: Stem Cell Reports. 2020 Mar 19;14(4):648–62. doi: 10.1016/j.stemcr.2020.02.006 (PMC7160308; doi:10.1016/j.stemcr.2020.02.006)
Supplement: Document S2. Article plus Supplemental Information [file mmc4.pdf]

Stem Cell Reports
Article
OPEN ACCESS

Generation of Retinal Pigment Epithelial Cells Derived from Human Embryonic Stem Cells Lacking Human Leukocyte Antigen Class I and II

Sandra Petrus-Reurer,[1,2,3,8] Nerges Winblad,[2,3,8] Pankaj Kumar,[2,3] Laia Gorchs,[5] Michael Chrobok,[6] Arnika Kathleen Wagner,[6] Hammurabi Bartuma,[1] Emma Lardner,[1] Monica Aronsson,[1] Álvaro Plaza Reyes,[2,3] Helder André,[1] Evren Alici,[6] Helen Kaipe,[5,7] Anders Kvanta,[1,9] and Fredrik Lanner[2,3,4,9,*]

[1]Clinical Neuroscience, Section for Ophtalmology and Vision, Karolinska Institutet, St. Erik Eye Hospital, 11282 Stockholm, Sweden
[2]Department of Clinical Sciences, Intervention and Technology, Karolinska Insitutet, 17177 Stockholm, Sweden
[3]Gynecology and Reproductive Medicine, Karolinska Universitetssjukhuset, 14186 Stockholm, Sweden
[4]Ming Wai Lau Center for Reparative Medicine, Stockholm Node, Karolinska Institutet, 17177 Stockholm, Sweden
[5]Department of Laboratory Medicine, Karolinska Institutet, 14152 Stockholm, Sweden
[6]Department of Medicine Huddinge, Center for Hematology and Regenerative Medicine, Karolinska Institutet, 17177 Stockholm, Sweden
[7]Clinical Immunology and Transfusion Medicine, Karolinska University Hospital, Huddinge, 14186 Stockholm, Sweden
[8]Co-first author
[9]Co-senior author
*Correspondence: fredrik.lanner@ki.se
https://doi.org/10.1016/j.stemcr.2020.02.006

SUMMARY

Human embryonic stem cell-derived retinal pigment epithelial (hESC-RPE) cells could serve as a replacement therapy in advanced stages of age-related macular degeneration. However, allogenic hESC-RPE transplants trigger immune rejection, supporting a strategy to evade their immune recognition. We established single-knockout beta-2 microglobulin (SKO-B2M), class II major histocompatibility complex transactivator (SKO-CIITA) and double-knockout (DKO) hESC lines that were further differentiated into corresponding hESC-RPE lines lacking either surface human leukocyte antigen class I (HLA-I) or HLA-II, or both. Activation of CD4+ and CD8+ T-cells was markedly lower by hESC-RPE DKO cells, while natural killer cell cytotoxic response was not increased. After transplantation of SKO-B2M, SKO-CIITA, or DKO hESC-RPEs in a preclinical rabbit model, donor cell rejection was reduced and delayed. In conclusion, we have developed cell lines that lack both HLA-I and -II antigens, which evoke reduced T-cell responses *in vitro* together with reduced rejection in a large-eyed animal model.

INTRODUCTION

Age-related macular degeneration (AMD) affects more than 180 million people globally and is the most common cause of blindness in industrialized countries among people over 60 years (Gehrs et al., 2006; Jonas et al., 2017). A potential regenerative treatment of AMD involves generation of autologous or allogeneic retinal pigment epithelial (RPE) cells from human pluripotent stem cells (hPSCs). Several groups have developed efficient protocols to produce RPE from hPSC morphologically and functionally comparable with native RPE (Choudhary et al., 2017; Hongisto et al., 2017; Klimanskaya et al., 2004; Lane et al., 2014; Maruotti et al., 2015; Osakada et al., 2009; Pennington et al., 2015; Plaza Reyes et al., 2016; Vaajasaari et al., 2011). In addition, several preclinical studies have shown that subretinal suspension or sheet transplants of hPSC-RPE can halt progression and even rescue photoreceptor loss or visual function (Diniz et al., 2013; Idelson et al., 2009; Kamao et al., 2014; Lu et al., 2009; Lund et al., 2006; Petrus-Reurer et al., 2017; Plaza Reyes et al., 2016; Sharma et al., 2019; Vugler et al., 2008), which has encouraged subsequent clinical studies in patients with AMD (da Cruz et al., 2018; Kashani et al., 2018; Mandai et al., 2017; Schwartz et al., 2016; Song et al., 2015).

Although the eye is considered an immune privileged site, immunorejection is frequently observed when transplanting allo- or xenogeneic PSC-RPE to animal models (Ilmarinen et al., 2015, 2018; McGill et al., 2018; Sohn et al., 2015; Stanzel et al., 2014; Zhang and Bok, 1998). We have previously reported that xenogeneic hESC-RPEs may survive in the subretinal space of albino rabbits for extensive periods under intravitreal steroid immunosuppression, and similar findings were reported for allogeneic RPE derived from induced pluripotent stem cells (iPSCs) transplanted to non-human primates (Petrus-Reurer et al., 2017; Plaza Reyes et al., 2016; Sugita et al., 2017). When rejection occurs it typically involves both the innate immune system, which may activate recipient natural killer (NK) cells by lack of a cognate HLA-I (missing self) (Karre et al., 1986; Ljunggren and Karre, 1990), and the adaptive immune system where an antigen-presenting cell (APC) engulfs the donor cell, processes the mismatched HLA-I and HLA-II molecules into peptides, which in turn are presented and recognized by CD8+ cytotoxic and CD4+ helper T-cells via HLA-I and HLA-II molecules, respectively (indirect allorecognition). In addition, donor cell antigens coupled to HLA-II molecules on the APC activate CD4+ T-cells that also can trigger CD8+ T-cells, NK cells and antibody-producing B cells (Bradley et al., 2002; de Rham and Villard, 2011).

 © 2020 The Authors.
This is an open access article under the CC BY license (http://creativecommons.org/licenses/by/4.0/).

A solution to avoid immune rejection is to use autologous RPEs derived from iPSCs (Mandai et al., 2017). However, such personalized technology is costly and time consuming. An approach suggested to overcome these hurdles has been to establish an iPSC bank of "superdonors," by donor collection or genome-editing techniques, with a specific homozygous or edited HLA haplotype that could match a high proportion of the population (Deuse et al., 2019; Sugita et al., 2016a, 2016b; Taylor et al., 2012; Xu et al., 2019).

In this study, we generated and characterized hESCs and hESC-RPEs lacking surface presentation of HLA-I and -II through CRISPR/Cas9 targeting of *B2M* and *CIITA*. Our results support this strategy to overcome host-donor mismatch in non-autologous cell-based treatment of AMD and other hESC-derived cell replacement therapies.

RESULTS

B2M and CIITA Loci Edited by CRISPR/Cas9 to Obtain Single- and Double-Knockout Lines

Anchoring of HLA-I molecules to the cell membrane is dependent on the β_2 microglobulin protein and is encoded by the *B2M* gene (Nathenson et al., 1981). Consequently, loss of B2M leads to failure of HLA-I presentation on the cell surface. CIITA is a well-known HLA-II transactivator that activates HLA-II genes (Masternak et al., 2000). To disrupt their function, we used CRISPR/Cas9 with three sgRNAs targeting exon 1 or 2 of *B2M* (Figures 1A and S1A), or exon 2 or 3 of *CIITA* (Figure 1B), respectively, transfected into HEK293T cells. Insertion/deletions (indels) were detected in all samples, and sgRNAs B2M-1 and CIITA-5 had the highest percentage of cleaved DNA with 38.9% (B2M-1) and 30.5% (CIITA-5) efficiency (Figure 1C). HS980 hESC line was electroporated with pX459-(EF-1α)-B2M-1 (Figure S1B) and all single-cell clones were sequenced to determine the specific on-target mutation. Of note, Cas9 protein presence was not detected at day 9 when cells were plated for clonal expansion (Figure S1C). The hESC single-knockout B2M (hESC SKO-B2M) single-cell clone had a 1-bp insertion predicted to cause a frameshift mutation (Figure 1D, top chromatogram). After *B2M* knockout validation, the hESC SKO-B2M clone was electroporated with pX459-(EF-1α)-CIITA-5. An hESC double-knockout (hESC DKO) *B2M* and *CIITA* single-cell clone that had a 1-bp deletion predicted to cause a knockout of *CIITA* was chosen for further validation (Figure 1D, bottom chromatogram).

We performed paired-end whole-genome sequencing of wild-type hESCs (hESC WT), hESC SKO-B2M, and hESC DKO samples to evaluate putative off-target short nucleotide variants (SNV) and copy-number deletions (Table S3). First, we looked for specific changes at sites predicted by both Cas-OFFinder (Bae et al., 2014) and E-CRISP (Heigwer et al., 2014). The *B2M* gRNA generated 19,277 and *CIITA* gRNA generated 22,618 *in silico* predicted off-targets, respectively. CRISPR/Cas9-induced changes followed by clonal expansion would be expected to result in allele frequencies in line with heterozygote or homozygote changes, such as 0.5 or 1.0, which we also detected at the on-target sites at the *B2M* locus (chr15:45003753; C/CT; AF 1.0) and the *CIITA* locus (chr16:10989283; CA/C; AF 1.0) (Figure 1E). The only additional three changes were detected at lower allelic frequencies, indicating that these instead were acquired changes during culture and unrelated to the CRISPR/Cas9 targeting. Importantly, neither of these were in any known genes. We also searched predicted off-target loci within copy-number deletions and none of the predicted loci were found within homozygous copy-number deletions (Table S4). Moreover, we identified four and three heterozygous copy-number deletions overlapping with the predicted off-target loci for hESC SKO-B2M and hESC DKO samples, respectively, neither of which were in annotated exonic regions. Using an unsupervised approach, we explored if any SNVs had been introduced into known coding genes. This analysis identified 13 (11 SNPs and 2 indels) and 16 (13 SNPs and 3 indels) somatic SNVs within non-redundant exonic boundaries for hESC WT versus SKO-B2M, and SKO-B2M versus DKO samples, respectively; which, after filtering, resulted in three heterozygote SNVs, which were either silent, within the 3′ UTR, or a heterozygote nonsense mutation (Table 1). Functionally, neither of these mutations have been linked to disease or tumorigenicity.

After targeting the *B2M* locus, we evaluated the HLA-I protein knockout in both hESCs and hESC-RPEs. For that purpose, we decided to increase HLA-I expression by stimulating the cells with interferon gamma (IFN-γ). Titration experiments showed that treatment with 100 ng/mL IFN-γ for 2 days induced high expression of HLA-I in both hESCs and hESC-RPEs, and 5 days induced HLA-II upregulation in hESC-RPEs (Figures S2A and S2B).

Differentiated SKO-B2M hESC-RPEs showed characteristic pigmentation, cobblestone morphology, and expression of the epithelial marker ZO-1 (Figure 2A). Moreover, B2M was lost in SKO-B2M cells and HLA-I was not present on the cell surface, as shown by immunofluorescence and flow cytometry (Figures 2A–2D); although the intracellular molecule was detected in the cytoplasm (Figures 2A and 2C). Gene expression of pluripotency-, RPE-, and HLA-I and -II-related genes in SKO-B2M cells did not differ from hESC-RPE WT, indicating that the targeting did not affect any of the downstream phenotypical properties of hESC-RPE cells (Figure 2D). DKO hESC-RPEs stimulated with IFN-γ for 5 days also showed cobblestone morphology,

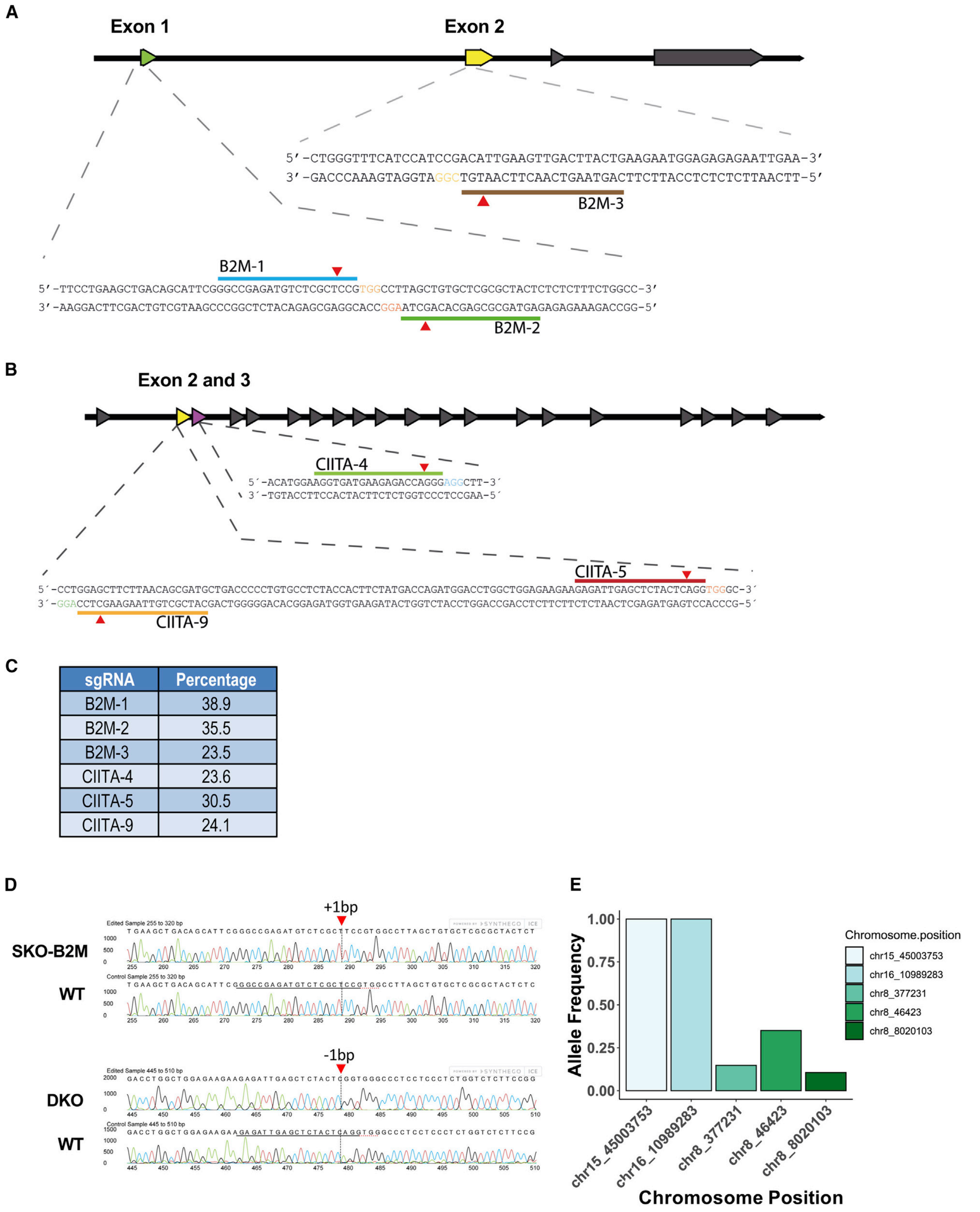

(legend on next page)

expression of ZO-1 and intracellular HLA-I but lack of B2M (Figure 2E). Moreover, neither intra- nor extracellular HLA-II were present in DKO cells as indicated by immunofluorescence and flow cytometry (Figures 2E and 2F). At a transcriptomic level, pluripotency genes (*NANOG* and *OCT3/4*), RPE genes (*BEST-1* and *RPE-65*), and HLA-I-related genes (*HLA-A*, *-B*, *-C*, except for *B2M* as for SKO-B2M cells) did not differ from WT. HLA-II-related genes (*HLA-DR*, *-DP*, *-DQ*) showed no expression, whereas *CIITA* was unaltered in DKO cells (Figure 2G).

hESC-RPEs Show Immunomodulatory Properties

We then evaluated the immunosuppressive capacity of the cells by co-culturing WT, SKO-B2M, or DKO cells with isolated human peripheral blood mononuclear cells (PBMCs) at a 1:1 ratio, with or without OKT-3 (anti-CD3 monoclonal antibody) stimulation. Notably, addition of hESC-RPEs to unstimulated PBMCs did not induce T-cell proliferation in any of the lines, whereas allogeneic PBMC control induced measurable proliferative responses (Figure 3A). Upon PBMC stimulation with OKT-3, WT and both SKO-B2M and DKO cells significantly decreased the proliferative response of CD8+ and CD4+ T-cells by 5- to 10-fold compared with stimulated PBMCs alone, demonstrating the general immunosuppressive ability of the derived RPE cells (Figure 3A).

SKO-B2M and DKO hESC-RPEs Exhibit Abolished T-Cell Reactivity *In Vitro*

Next, we assessed whether the hESC-RPEs could promote T-cell response under inflammatory conditions. hESC-RPEs were pre-stimulated with IFN-γ 100 ng/mL for 2 days before co-culturing with PBMCs. This treatment alone did not induce T-cell proliferation (data not shown). However, when the T-cell stimulatory signals interleukin-2 (IL-2) (1 ng/mL) and anti-CD28 antibody (aCD28, 1.25 μg/mL) were added to the co-culture, we observed up to a 4-fold increase in CD8+ T-cell proliferation compared with PBMCs only, at ratios of 1:20 and 1:50 of hESC-RPEs to PBMCs (Figure S3A). Purified CD8+ T-cells but not CD4+ T-cells, showed significant reduction of IFN-γ secretion in the presence of SKO-B2M cells compared with WT; whereas IFN-γ amounts were reduced by both CD8+ and CD4+ T-cells when cultured with DKO cells, thus confirming abolished activation of cytotoxic and helper T-cells (Figure 3B). Further analysis of T-cell ligands expressed on the surface of both hESCs and hESC-RPEs demonstrated that PD-L1, but not PD-L2, was robustly detected in WT and DKO cells both under non-stimulated and stimulated conditions (Figure S3B). In addition, after IFN-γ stimulation, a small population in all analyzed hESC-RPE lines expressed the co-stimulatory molecule CD80 but not CD86, which was instead present to a greater degree in hESCs. The expression of co-stimulatory molecules could potentially give hESC-RPE APC properties and, consequently, the ability to mount a T-cell reaction by themselves (Figure S3B).

SKO-B2M and DKO hESC-RPEs Increase NK Cell Activation but Not Cytotoxicity under Inflammatory Conditions *In Vitro*

In co-cultures with freshly isolated and overnight IL-2-activated human NK cells, we first found that HLA-I levels were induced with hESC-RPE differentiation (data not shown) coinciding with older hESC-RPEs (days d55–d63) being less sensitive to NK cell cytotoxicity than younger cells (days d44–d50) and hESCs at a 10:1 effector:target ratio (Figure S3C). Degranulation analysis measured by CD107 expression upon co-culture of hESC-RPEs and overnight IL-2-activated NK cells showed a donor-dependent response, stronger in NK donor 1 compared with NK donors 2 and 3 (Figure 3C). Of note, NK cells from all three donors showed no degranulation in the absence of target cells, and showed robust stimulation with positive controls K562 target cells (erythroid leukemia) or phorbol-myristate-acetate/ionomycin stimulation (chemical stimulation) (Figure S3D). The edited SKO-B2M and DKO cell lines did not differ in NK cell activation compared with WT in non-inflammatory conditions (Figure 3C). Interestingly, IFN-γ pre-stimulation of hESC-RPEs showed a significantly decreased response in all analyzed donors, especially for WT cells relative to their unstimulated samples; and the edited lines showed a significantly increased response compared with their respective stimulated WT hESC-RPE co-cultures (Figure 3C). In addition, when NK cells were mixed with unstimulated SKO-B2M or DKO cells at a 10:1 effector:target ratio, cytotoxicity levels also showed to be donor dependent, and strikingly similar between WT and edited lines lacking HLA-I or HLA-I and -II molecules (Figures 3D and S3D). IFN-γ (100 ng/mL) pre-stimulation of hESC-RPE for 2 days did

Figure 1. B2M and CIITA sgRNA Evaluation

(A) Schematic illustration of the human *B2M* locus, including sgRNA target sites.
(B) Schematic illustration of the human *CIITA* locus, including sgRNA target sites.
(C) Frequency of indel occurrence generated by each sgRNA in CRISPR/Cas9-edited HEK293T cells.
(D) Indel analysis obtained by Sanger sequencing in hESC SKO-B2M (top chromatogram) and hESC DKO (bottom chromatogram).
(E) Bar graph representing allele frequency in specific chromosomal positions from off-target analysis of whole-genome sequencing data.
See also Figure S1, Tables S3, and S4.

Table 1. Somatic SNVs Identified Using MuTect2 with Allele Frequency $\geq$ 0.25 and Read Depth $\geq$ 10

Chromosome	Position	Reference	Altered	AD	AF	Gene	Type	SNVs	Change
**Sample 1: hESC SKO-B2M**									
chr3	194077829	G	A	16; 14	0.467	LRRC15	3′ UTR	SNP	
chr11	64820793	A	G	15; 8	0.348	NAALADL1	Silent	SNP	
chr13	45969047	CTT	C	10; 18	0.63	SLC25A30	3′ UTR	DEL	
chr15	22945136	C	T	18; 9	0.346	CYFIP1	Nonsense_Mutation	SNP	ENSP00000324549:p.Gln403*
chr15	45003753	C	CT	0; 24	1	B2M	Frame_Shift_Ins	INS	ENSP00000452780:p.Ser4fs
**Sample 2: hESC DKO**									
chr16	10989283	CA	C	0; 12	1	CIITA	Frame_Shift_Del	DEL	ENSP00000316328:p.Ser66fs

Total non-redundant exonic regions considered in the analysis: 229,235 (26,201 unique genes).
AD, allelic depths for the reference and altered alleles in the order listed; AF, allele fraction of the event in the hESC WT and hESC SKO-B2M (sample 1); or hESC SKO-B2M and hESC DKO (sample 2).
See also Figure S1, Tables S3 and S4.

not show any reproducible significant effect among the three donors, although a trend in reduced cytotoxicity could be appreciated in donor 2, in addition to a general maintenance or decrease in cytotoxicity in the edited lines compared with their respective WT hESC-RPE co-cultures (Figure 3D). Further evaluation of several NK cell ligands by flow cytometry showed that HLA-I (including HLA-C) was expressed in hESCs and WT cells, and this expression increased after 2 or 5 days of IFN-γ stimulation. Other ligands tested included MIC-A/B (recognized by the activating NK cell receptor NKG2D) that showed a slightly higher expression in hESCs than in hESC-RPEs; proliferating cell nuclear antigen (recognized by the activating NK cell receptor NKp30) that was not upregulated in any of the cell types; CD112 (a ligand for the activating receptor DNAM-1 and the inhibitory receptor TIGIT) that was highly expressed in all cell types tested; and CD155 (another ligand for DNAM-1, TIGIT, and CD96, which has been ascribed with both activating and inhibitory functions) that was expressed at lower levels in both SKO-B2M and DKO compared with WT cells and IFN-γ stimulatory conditions (Figure S3E). In addition, WT cells exhibit significant expression levels of CD47, CD55, and CD59, which have been suggested previously to function as inhibitors of NK cell activity (Deuse et al., 2019; Finberg et al., 1992; Marcenaro et al., 2003) (Figure S4A). Furthermore, analysis of NKG2A expression and several killer-cell immunoglobulin-like receptors (KIR)—KIR2DL1, KIR3DL1 KIR2DL5, KIR3DL2, and KIR2DL2/L3/S2 (clone [CH-L]) that recognize different HLA molecules on the donor NK cells showed a donor distinct pattern (Figure S4B), which could contribute to the different susceptibility of activation and killing potential of the donor NK cells. As expected, expression of NKG2A (binding to the HLA-E molecule) was consistently elevated in the three donors compared with the remaining KIRs that were analyzed. Because the hESCs we used express ligands for KIR2DL1 (HLA-C*04), KIR2DL2 (HLA-C*07), KIR3DL1 (HLA-A*32, HLA-B*38), KIR3DL2 (HLA-A*68), and NKG2A (HLA-E), we studied the degranulation response of NK cells expressing the specific KIRs by the hESC-RPEs. With our panel of antibodies, we were able to categorize NK cells expressing KIR2DL1, KIR2DL2 (detected by KIR2DL2/L3/S2), KIR3DL1, and NKG2A. As in the analysis of total NK cells, KIR+ NK cells showed variation in CD107a expression between the donors (Figures S4B and S4C). However, a reduction in response toward WT cells stimulated with IFN-γ could be observed in all KIR+ NK cells from all donors (Figure S4C). This inhibition was less potent in SKO-B2M and DKO cells, probably due to a failure to upregulate HLA-I in response to IFN-γ stimulation.

Immunogenicity of WT hESC-RPEs upon Subretinal Injection in a Xenograft Model

We have previously shown that WT cells can integrate in the subretinal space of albino rabbits under intravitreal immunosuppression with triamcinolone (TCA) (Plaza Reyes et al., 2016). However, we also noted that integration efficiency varied considerably, and pigmented areas of integrated cells were frequently lost over time, suggestive of immunorejection. We analyzed this in more detail by observing evidence of cell infiltration in the transplanted area on consecutive spectral-domain optical coherence tomography (SD-OCT) scans. As expected, subretinally transplanted WT cells under TCA immunosuppression displayed homogeneous monolayer integration as demonstrated by a distinct pigmented area on multicolor-confocal

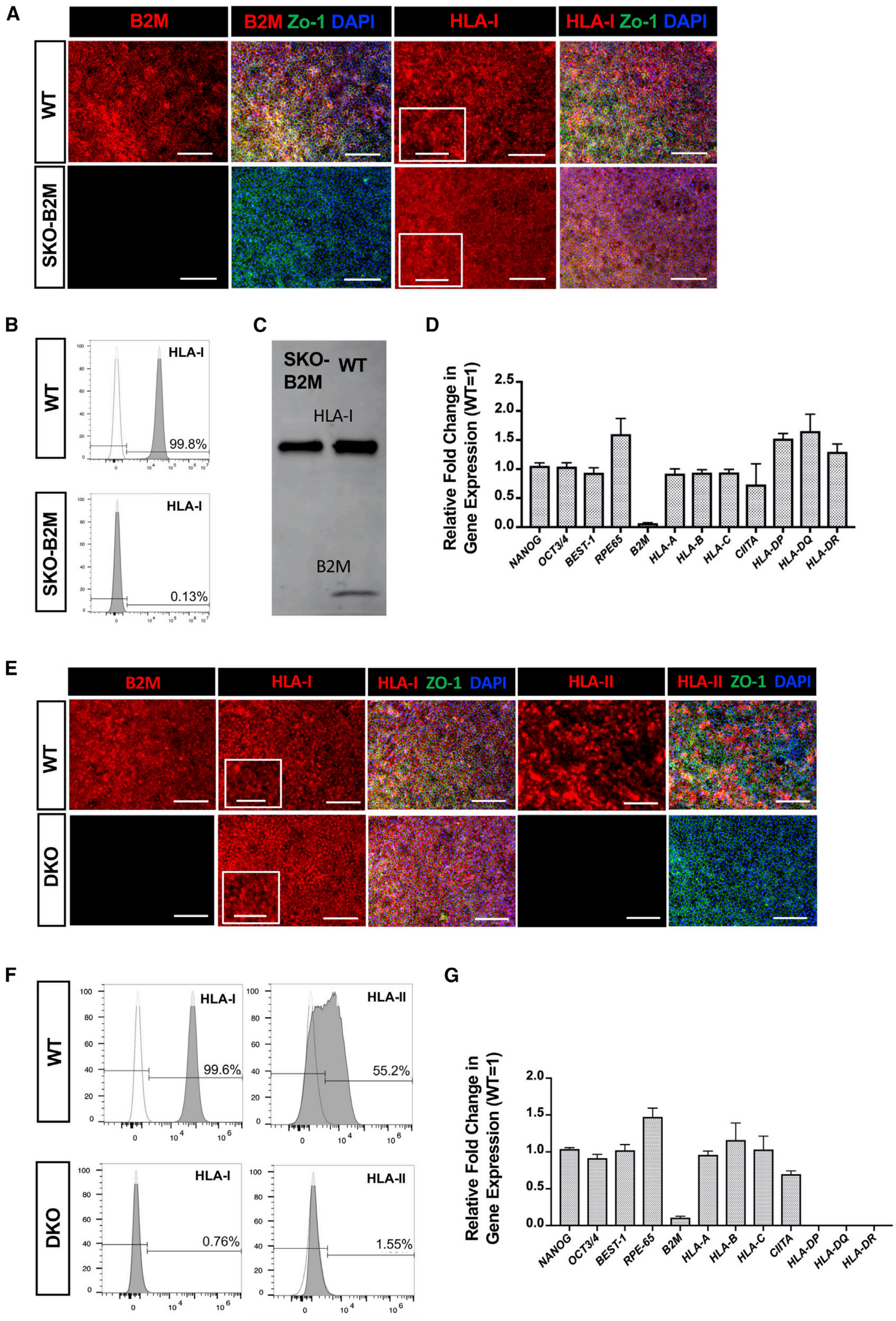

(legend on next page)

scanning laser ophthalmoscopy and a hyperreflective subretinal layer on SD-OCT (Figure 4A). However, at later time points, a pronounced mass was progressively formed in the subretinal space that displaced the pigmented donor cell layer and was accompanied by a thickening of the choroid, suggestive of immunorejection (Figure 4A; Video S1). Histological analysis confirmed that infiltrates consisted of densely packed, mainly mononuclear, inflammatory cells (Figure 4B). As the reaction progressed, the pigmented donor cells were gradually lost, and the subretinal infiltrate and choroidal thickening regressed, eventually leaving an atrophic retina and choroid (Figures 4B and 4C). Immune cells from both the innate (NK cells and macrophages) and the adaptive (CD3+ T-cells) immune system were present at different rejection stages compared with non-rejected eyes, whereas B cells (CD79a^{+}) emerged in early and late stages but not at the onset of rejection (Figure 4B). Human HLA-I was expressed in the transplanted WT cells even in later stages when cell shape and integrity had deteriorated, whereas HLA-II only appeared positive in cases of more prominent rejection (early and late) (Figure 4B). We next compared the rejection rates over time of WT transplanted animals using SD-OCT. In non-immunosuppressed animals, rejection was detected in all eyes within 7 days after transplantation, whereas in TCA-treated animals, subretinal infiltration was rarely seen after 1 week, and even after 30 days only half of the transplanted eyes showed signs of rejection (Figures 4C and 4D). By 90 days, rejection was observed in most of the transplanted eyes, also with TCA immunosuppression.

Immunogenicity of SKO-B2M, SKO-CIITA, and DKO hESC-RPEs upon Subretinal Injection in a Xenograft Model

We next assessed the immune response after subretinal transplantation of SKO-B2M and DKO without TCA immunosuppression in the xenogeneic model. Both types of gene-edited cells showed significantly lower rates of early (1 week) rejection compared with WT cells (Figures 5A and 5B). Interestingly, DKO cells did not survive better than SKO-B2M in the *in vivo* setting (Figure 5B). To examine the role specifically of HLA-II we also established single-knockout *CIITA* hESC-RPEs (SKO-CIITA) that lacked HLA-II expression while still being able to present HLA-I (Figures S5A–S5D). SKO-CIITA cells also showed a significantly reduced rejection, similar to the cells lacking HLA-I or HLA-I and -II (Figures 5A and 5B). Immune cells from both the innate (NK cells and macrophages) and the adaptive (CD3+ T-cells and CD79a+ B cells) immune system were present 7 days after subretinal transplantation of WT cells, unlike DKO where CD79a+ cells were not detected (Figures S5E and S5G). Regardless, all immune cell types were present 30 days after transplantation of either SKO-B2M or DKO cells (Figures S5F and S5H). Furthermore, serum from transplanted rabbits was collected at different time points and evaluated by flow cytometry for presence of anti-human (graft) antibodies (Figure 5C). Without immunosuppressive treatment, anti-human antibodies were detected 7 days after WT transplantation, but were only detectable after 14 days (in 4-fold significantly lower levels compared with WT) in SKO-B2M-, SKO-CIITA-, and DKO-transplanted animals. After 30 and 90 days, anti-human antibodies were widely detected in all transplantation conditions, albeit at lower amounts in the gene-edited compared with WT cells (Figure 5C). Thus, both early and late xenorejection of either SKO-B2M, SKO-CIITA, or DKO were delayed compared with WT cells.

DISCUSSION

We have shown that targeting *B2M* and *CIITA* genes in hESCs using CRISPR/Cas9 technology eliminates cell surface presentation of HLA-I and HLA-II proteins, respectively. In addition, these cells can be further differentiated

Figure 2. Characterization of the SKO-B2M and DKO hESC-RPEs
(A) Immunofluorescence images of WT and SKO-B2M showing B2M, HLA-I, and ZO-1 expression. Magnified box for HLA-I shows dotted extracellular pattern in WT cells.
(B) Representative flow cytometry histogram showing the percentage of WT and SKO-B2M expressing extracellular HLA-I. Dotted line histogram shows HLA-I FMO (negative control used for gating).
(C) Western blot showing the HLA-I and B2M protein expression of WT and SKO-B2M cells.
(D) Gene expression analysis of HLA- and RPE-related genes in the targeted hESC-RPEs. Values are normalized to *GAPDH* and displayed as relative to WT cells.
(E) Immunofluorescence images of WT and DKO cells showing B2M, HLA-I, and ZO-1 expression. Magnified box for HLA-I shows dotted extracellular pattern in WT cells.
(F) Representative flow cytometry histogram showing the percentage of WT and DKO cells expressing extracellular HLA-I. Dotted line histogram shows HLA-I FMO (negative control used for gating).
(G) Gene expression analysis of pluripotent and HLA-related genes in the targeted hESC-RPEs. Values are normalized to *GAPDH* and displayed as relative to WT.
Bars represent mean ± SEM from three independent experiments.
Scale bars, 100 μm (A and E) and 50 μm zoom-in (A and E). Molecular weight of HLA-I = 43 kDa; B2M = 12 kDa. See also Figure S2.

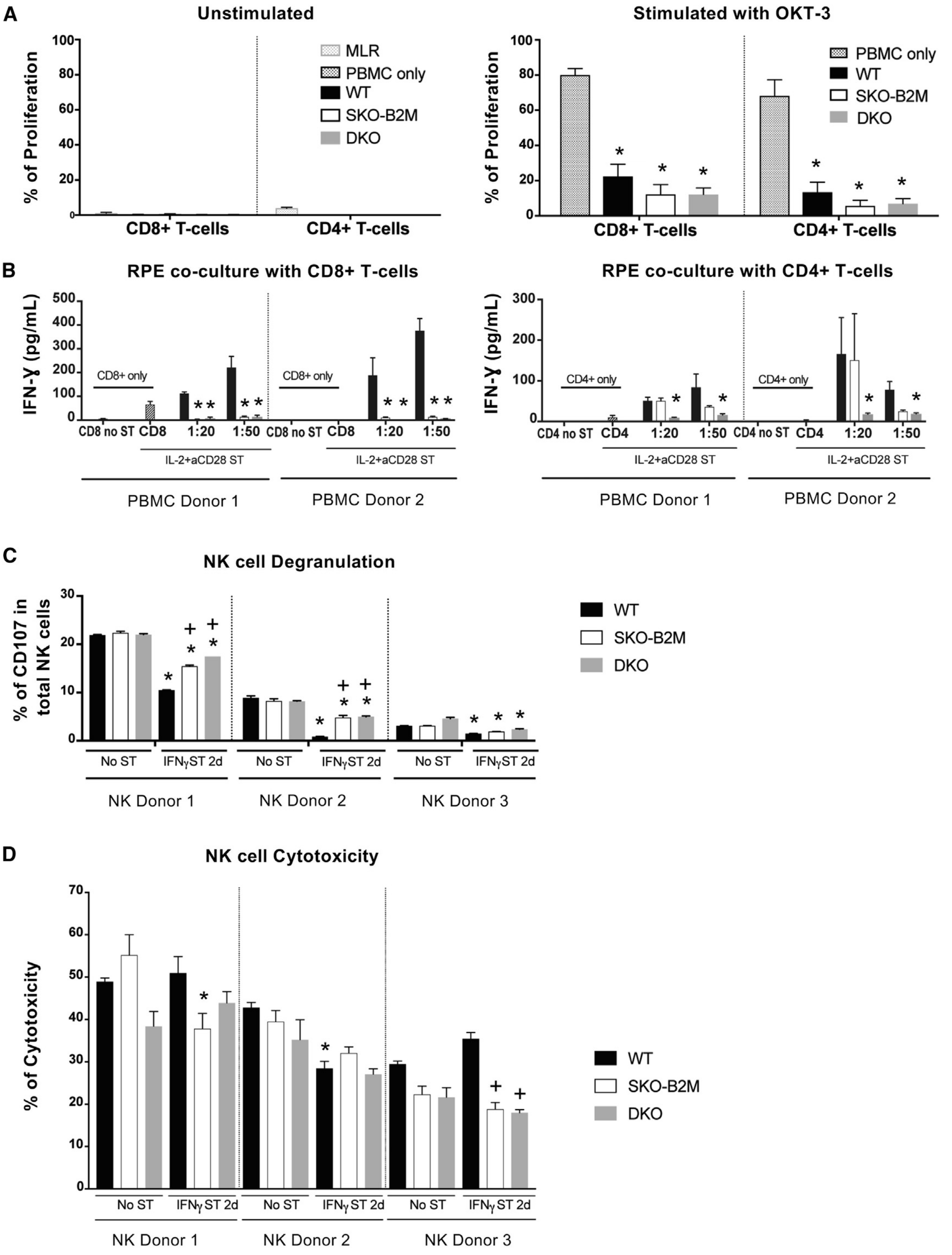

Figure 3. *In Vitro* Immunogenicity Assessment of WT, SKO-B2M, and DKO hESC-RPEs

(A) Graphs representing the percentage of proliferative CD8+ or CD4+ cells upon 5 days co-culture of PBMCs from three different donors with 2 days IFN-γ 100 ng/mL pre-stimulated WT, SKO-B2M, and DKO cells at 1:1 hESC-RPE:PBMC ratio with (right panel) or without (left panel) OKT-3 stimulation. Unstimulated PBMCs only and mixed allogenic PBMC donors (mixed lymphocyte reactions [MLR]) were used as negative and positive controls, respectively, to assess T-cell induction upon hESC-RPE co-culture; and OKT-3 stimulated PBMCs were used as positive control to evaluate suppression of T-cell proliferation upon hESC-RPE co-culture.

(legend continued on next page)

into hESC-RPEs that also lack HLA-I, HLA-II, or both HLA-I and -II presentation but retain native RPE cell properties. We also show that SKO-B2M cells reduce CD8+ but not CD4+ T-cell activation, that DKO reduce both CD8+ and CD4+ T-cell activation, and that both lines increase NK cell activation but not cytotoxic activity compared with WT cells under inflammatory conditions. When transplanted into the rabbit xenograft model without immunosuppression, all gene-edited lines reduce early rejection rates and delay anti-human antibody production associated with late rejection.

Engineering cells introduces the risk of off-target changes. Nonetheless, it is encouraging that we did not detect any signs of deleterious mutations or deletions in the predicted off-target sites after *B2M* or *CIITA* targeting neither defective properties in morphology nor characteristic marker expression in either SKO-B2M or DKO cells, suggesting that the mutational load is modest during our genome editing procedure. Absence of predicted off-target changes may be linked to the fact that we have performed clonal selection, which may eliminate less frequent off-target editing that may be detected if the initial bulk culture would have been expanded without clonal selection. However, changes in the genome can also occur during clonal selection and subsequent culture. Indeed, we did detect four heterozygote changes in known genes that may be spontaneous or CRISPR/Cas9 induced. Irrespectively, it is important to note that neither of these heterozygous changes have been linked to disease or tumorigenicity.

Despite the immunosuppressive phenotype of RPE cells, our study also shows that they can still induce a T-cell response under certain conditions *in vitro*: (1) that a specific RPE:immune cell ratio is achieved and (2) that inflammatory/stimulatory molecules, such as IFN-γ or IL-2 are present. In our setting, we show that HLA-I and HLA-II mismatched cells can trigger cytotoxic CD8+ and helper CD4+ T-cells, which would eventually lead to both acute and chronic graft rejection. However, if neither HLA molecule is present, T-cell response is clearly avoided. Interestingly, we found a population of approximately 5% among the IFN-γ-stimulated hESC-RPEs that showed upregulation of the co-stimulatory ligand CD80, which would confer the ability to induce an immune response without APC.

Our data suggest that the "missing self" phenotype (lack of HLA-I or both HLA-I and -II) of the engineered hESC-RPEs does increase the innate NK cell activation of the mismatched HLA-I and/or HLA-II WT cells. Such phenotype was observed only after inflammatory stimulation of the hESC-RPEs, but further studies are needed to explore if this is specific for hESC-RPEs despite their immunosuppressive properties. This finding suggests that the incorporation of an NK cell inhibitory ligand, such as HLA-E or the recently suggested cell surface molecules CD47, CD55, and CD59, may be beneficial to avoid rejection of grafted cells by the innate immune system (Deuse et al., 2019; Finberg et al., 1992; Gornalusse et al., 2017; Marcenaro et al., 2003; Sugita et al., 2018). Interestingly, our derived hESC-RPEs endogenously express high levels of CD47, CD55, and CD59, which could in part explain the lack of difference in NK cell cytotoxicity regardless of the HLA edits. Our degranulation assay showed a distinct difference in responsiveness dependent on donor, which correlated with the outcome of the cytotoxic assay. The level of activation might be subjected to NK cell education, i.e., expression of specific KIRs that recognize the presence of certain HLA molecules, such as HLA-C2, for the 2DL1 KIR present in the donor NK cells (Pegram et al., 2011). However, the degree and differences seen in degranulation and cytotoxicity when HLA-I was absent (SKO-B2M or DKO) or increased (IFN-γ stimulation), could be explained by the changes in expression of certain ligands (e.g., CD155), and may be specific to hESC-RPEs. Overall, NK cell activation toward RPE cells was limited to a few donors, and did not result in measurable release of cytokines (IFN-γ) (data not shown). In addition, these NK studies show no difference in NK cell cytotoxicity after removal of HLA-I or both HLA-I and -II in normal or inflammatory conditions, thus suggesting that HLA-II has no effect on NK cells and reinforcing the possibility to use these double

(B) Bar graphs showing the secretion of IFN-γ produced by either CD8+ or CD4+ isolated T-cells from two different donors after 5 days co-culture with WT, SKO-B2M, or DKO cells at 1:20 and 1:50 ratios (hESC-RPE:PBMC) with the presence of IL-2+aCD28 stimulation (and 2 days IFN-γ 100 ng/mL hESC-RPE pre-stimulation). CD8+ or CD4+ only were used as negative controls unstimulated (No ST) or IL2+aCD28 stimulated (ST).

(C) Bar graphs showing the percentage of NK degranulation by CD107-positive expression in the total NK cells when co-cultured with WT, SKO-B2M, and DKO cells unstimulated or 2 days IFN-γ 100 ng/mL pre-stimulation from three different donors.

(D) Bar graph showing the percentage of cytotoxicity of the unstimulated or 2 days IFN-γ 100 ng/mL pre-stimulated WT, SKO-B2M, or DKO cells (target) measured by chromium release of the killed cells by the freshly isolated and overnight IL-2-stimulated NK cells (effector) from three different donors at 10:1 effector:target ratio. NK cells were freshly isolated from human blood PBMCs, further separated (CD56 MACS isolation kit) and activated with IL-2 overnight before co-culture with hESC-RPEs.

Bars represent mean ± SEM from three independent experiments. (A) $^{*}p < 0.0001$ compared with PBMC only with OKT-3; (B) $^{*}p < 0.0001$ (CD8+), $^{+}p < 0.05$ (CD4+) compared with respective WT; (C) $^{*}p < 0.0001$ compared with respective No ST cell line per donor, $^{+}p < 0.0001$ compared with respective WT per donor; and (D) $^{*}p < 0.01$ compared with respective No ST cell line per donor, $^{+}p < 0.001$ compared with respective WT per donor. See also Figures S3 and S4.

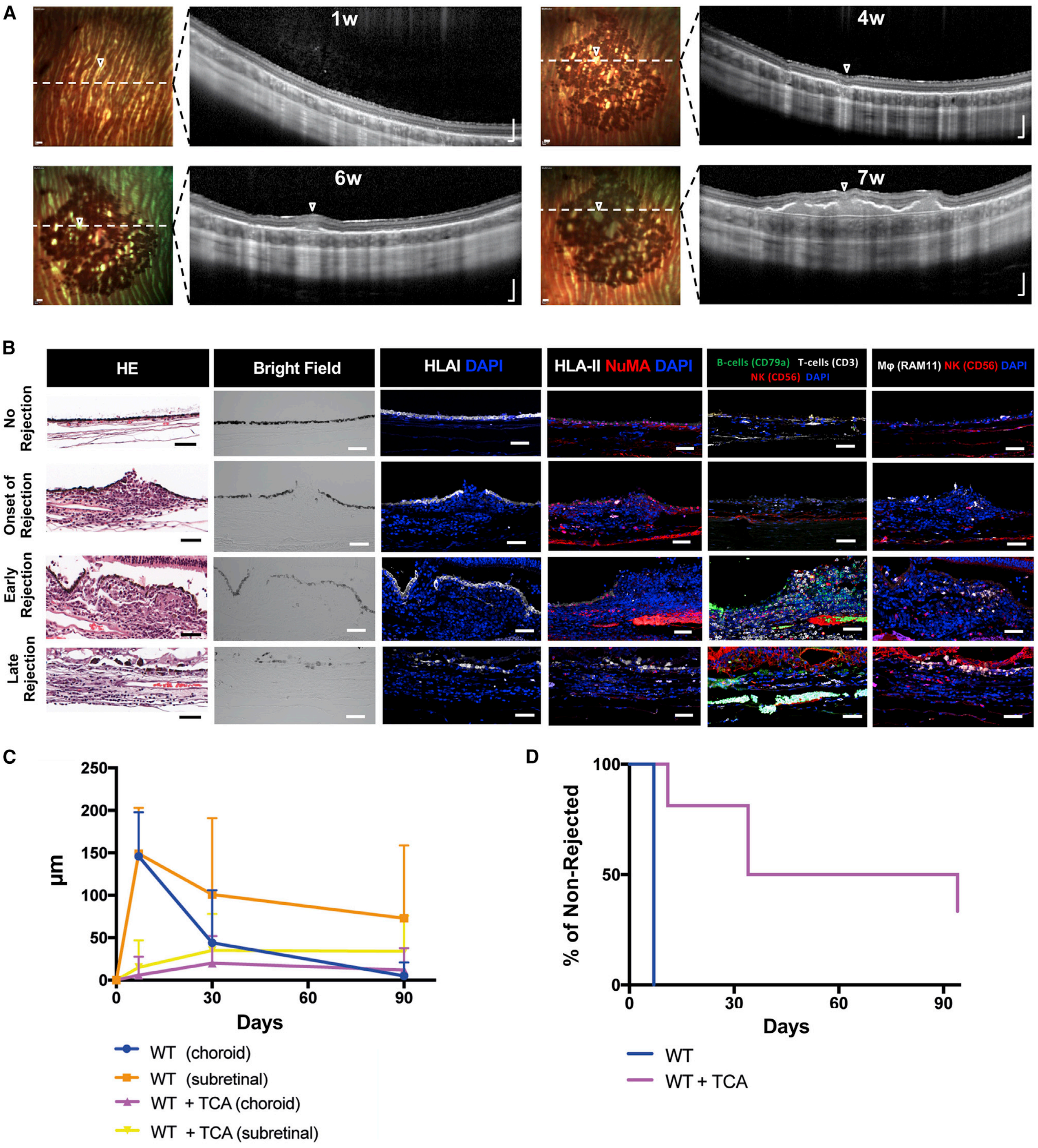

Figure 4. Transplantation of WT hESC-RPEs in the Xenograft Model

(A) Time course multicolor-confocal scanning laser ophthalmoscopy and SD-OCT images of the injected areas with WT cells in the subretinal space under TCA treatment. Dashed white lines indicate SD-OCT scan plane. Open arrowheads indicate focal areas of rejection.

(B) Bright-field, H&E, and immunofluorescence images representing different rejection patterns after subretinal injection of WT cells. Images show the expression of human HLA-I, HLA-II, and NuMA, in addition to rabbit immune cells: CD3 for T-cells, CD56 for NK cells, CD79a for B cells, and RAM11 for macrophages.

(legend continued on next page)

gene-edited cells to maximize the escape from both T and NK cells. Nonetheless, another possible strategy to counteract HLA-I- and -II-independent NK cell effect could reside on the selection of a donor hESC-RPE line from a cell bank based on reactivity with the recipient NK cells, aiming to avoid activation of NK cells that could elicit strong responses when the specific HLA epitopes are missing in the hESC-RPEs.

Without immunosuppression, donor cells triggered rapid rejection in our xenogeneic transplantation model, whereas TCA-mediated immunosuppression delayed and sometimes prohibited rejection. The degree and time of rejection varied greatly, implying that local factors were involved. For instance, mechanical damage to Bruch's membrane during transplantation could compromise the immune-privileged subretinal space, thus allowing invasion by immune cells as suggested previously (Petrus-Reurer et al., 2017). The cells infiltrating into the graft belonged to both the innate and adaptive immune systems in all stages of rejection. Evaluation of the T-cell subtypes (CD8+ or CD4+) in the infiltrates could not be performed due to lack of reliable immunoreaction with anti-rabbit antibodies but deserves further exploration since a reduction in CD8+ cells after SKO-B2M transplantation, and a reduction of both CD8+ and CD4+ T-cells after DKO transplantation, would be expected, which could explain the milder rejection phenotype compared with WT cells. Contrary to what we expected from the *in vitro* results, transplantation of DKO cells was not superior to either SKO-B2M or SKO-CIITA, possibly suggesting that either CD4+ T-cells are pre-activated (i.e., rendering HLA-II loss ineffective) or that, despite delayed T-cell-mediated rejection, NK cells may eventually counter react it. The fact that all hESC-RPE lines showed rejection over time may be due to antibodies produced against human cells via indirect allorecognition (macrophages, dendritic cells, or B lymphocytes recognizing grafted cells) that activate CD4+ T-cells (de Rham and Villard, 2011; Fox et al., 2001; McGill et al., 2018; Sohn et al., 2015; Sugita et al., 2017).

Our *in vitro* and *in vivo* observations show that DKO cells have the potential to partially evade the adaptive immune system, making these cells attractive candidates as universal donors. Nevertheless, the innate immune system, including NK cells, may still be activated as discussed above. It is important to note that the immune system is an essential first-line defense against tumor formation and infection, and generation of immune evasive cells could also escape this defense mechanism. Therefore, a fail-safe/suicidal cassette system enabling the clearance of engineered cells may have to be integrated before clinical consideration (Liang et al., 2018). Also, minor antigens and sugars that do not require HLA molecule presentation or fragments of dead grafted cells could be also be a source of APC recognition and immune activation.

In this study, donor cells were transplanted in a cross-species model, whereas in a comparable clinical setting donor cells would be allogenic where rejection is expected to be less pronounced (Fox et al., 2001). It is well known from other models that xenogeneic rejection involves not only allogeneic pathways but also innate neutrophil and macrophage responses toward non-self oligosaccharides that precede T-cell activation. The stronger activation of the innate immune system may in turn explain why conventional immunosuppressants are less effective in preventing xenogeneic rejection (Hawthorne et al., 2016). The rabbit immune system is in this respect poorly studied but it is reasonable to infer the involvement of xeno-specific rejection pathways, as suggested by our data. Therefore, our finding that removal of HLA-I, HLA-II, or both HLA-I and -II delayed rejection is encouraging since a reduced rejection, as observed with either gene-edited hESC-RPEs, is also expected to translate into an allogeneic human setting. However, and ideally, the immune reactions demonstrated in this study should also be confirmed in a preclinical allogeneic model, in line with the previously demonstrated observations in non-human primates transplanted with allogeneic WT iPSC-RPEs (Sugita et al., 2017).

In conclusion, we show that WT hESC-RPEs can induce both adaptive and innate immunoresponses *in vitro* and *in vivo*, but the loss of surface HLA-I and HLA-II molecules leads to a decreased immunogenicity, thus comprising a first step toward universal-donor cells for cell replacement therapy in AMD and related conditions.

EXPERIMENTAL PROCEDURES

Cell Culture and Differentiation

hESC line HS980 was established and cultured under xeno-free and defined conditions on rhLN-521, and passaged as described previously (Rodin et al., 2014a, 2014b). For differentiation, cells were

(C) Graph showing the thickness of either the choroidal or the subretinal space of eyes transplanted with WT cells with or without TCA immunosuppression through time (days d0, d30, d60, and d90). The rejection thickness was obtained by subtracting the values of a non-rejected area as described in the Experimental Procedures section.
(D) Kaplan-Meyer graph representing the number of non-rejected eyes up to 90 days after transplantation of WT cells or WT cells + TCA. Bars represent mean ± SEM from WT = 18; WT + TCA = 15 eyes for all time points in (C) (the value of four eyes in the d90 time point was carried forward from the last observation due to their planned enucleation at d30); and WT = 18; WT + TCA = 15 eyes in (D). Scale bars, 200 μm (A) and 50 μm (B).

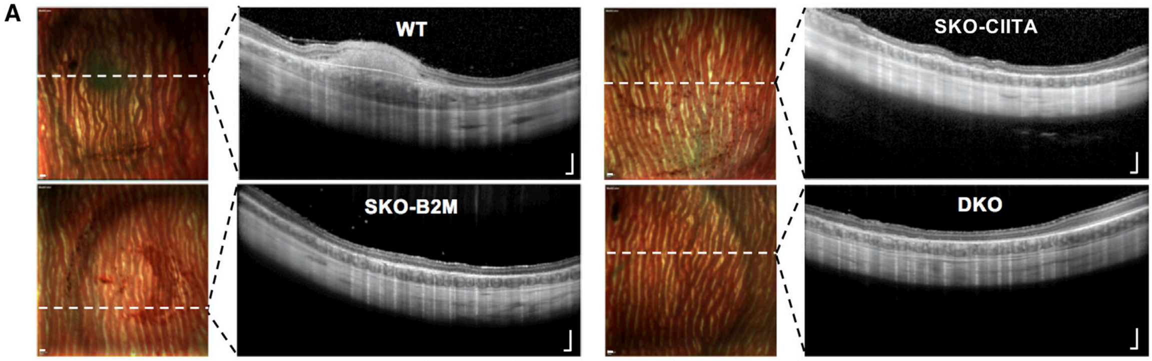

B

Rejection d7	WT	SKO-B2M*	SKO-CIITA*	DKO*
+	28	11	14	15
-	0	8	7	7

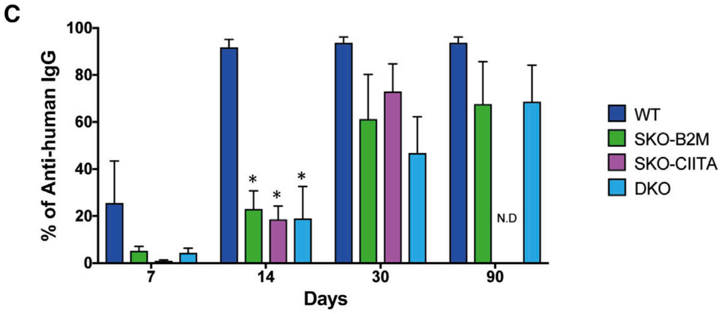

Figure 5. Transplantation of SKO-B2M, SKO-CIITA, and DKO hESC-RPEs in the Xenograft Model

(A) Day 7 multicolor-confocal scanning laser ophthalmoscopy and SD-OCT images of representative rabbits that received WT, SKO-B2M, SKO-CIITA, or DKO cells without TCA treatment. Dashed white lines indicate the SD-OCT scan plane.

(B) Table summarizing the number of rejected or non-rejected eyes at day 7 upon transplantation of WT, SKO-B2M, SKO-CIITA, or DKO cells.

(C) Bar graph showing the percentage of anti-human immunoglobulin G antibodies measured by flow cytometry present in rabbit serums after transplantation of WT, SKO-B2M, SKO-CIITA, or DKO without TCA that bound to WT cells at 7, 14, 30, and 90 days.

Bars represent mean ± SEM from five rabbits per condition. In (B), *$p < 0.001$ compared with WT; and (C), *$p < 0.001$ compared with respective WT per time point. Scale bars, 200 μm (A). N.D., no data available. See also Figure S5.

plated at a density of 2.4×10^4 cells/cm^2 on 20 μg/mL hrLN-111-coated dishes using NutriStem hESC XF medium and Rho-kinase inhibitor during the first 24 h. NutriStem hESC XF without basic fibroblast growth factor and transforming growth factor β was then replaced and from day 6 after plating, 100 ng/mL of activin A was added to the medium for a total of 5 weeks.

Genome Editing of hESCs

hESC line HS980 was modified at *B2M* and *CIITA* loci using CRISPR/Cas9 plasmid constructs. First, high cutting efficiency sgRNAs were identified through screening in HEK293T. Second, gene editing in hESCs was first done at the B2M locus, followed by puromycin selection and clonal expansion. An SKO-B2M clone was then edited at the CIITA locus, followed by puromycin selection and clonal expansion. For both of the generated cell lines, on-target edits were determined by Sanger sequencing, and the characterization was based on detection of HLA-I and -II proteins under inflammatory conditions (IFN-γ, 100 ng/mL). Finally, potential off-target edits were analyzed by whole-genome sequencing.

T-Cell and NK Cell Co-cultures with hESC-RPE Lines

PBMCs were isolated from buffy coats by density gradient centrifugation. PBMCs were either stained using the CellTrace CFSE Cell Proliferation Kit; or CD4+, CD8+, and CD56+ negatively isolated with MACS beads and co-cultured with day 30 unstimulated or 2 day IFN-γ (100 ng/mL) pre-stimulated irradiated or chromium-labeled hESC-RPEs plated at a cell density range of 1×10^3 (1:500) to 5.5×10^5 (1:1) cells/cm^2 depending on the assay. Overnight IL-2-activated hNK cells were used for ^{51}Cr release and degranulation assays and assay-dependent stimulatory molecules were added. Co-cultures were maintained for 4 h (degranulation, cytotoxicity) or 5 days (T-cell proliferation) at 37°C.

Subretinal Transplantation and *In Vivo* Imaging

Dissociated hESC-RPEs were injected (50 μL; 50,000 cells) subretinally using a transvitreal pars plana technique. SD-OCT and confocal scanning laser ophthalmoscopy was performed to obtain horizontal cross-sectional B-scans and en face fundus *in vivo* images, respectively.

SUPPLEMENTAL INFORMATION

Supplemental Information can be found online at https://doi.org/10.1016/j.stemcr.2020.02.006.

AUTHOR CONTRIBUTIONS

S.P.-R., N.W., L.G., M.C., A.K.W., E.L., A.P.R., H.B., and A.K. performed the experiments. S.P.-R. performed *in vitro* and *in vivo* experiments. N.W. derived the gene-edited lines with CRISPR/Cas9. P.K. performed off-target analysis. H.B., M.A., and H.A. contributed to the animal work. H.K., H.A., and E.A. planned the experiments and contributed to discussions. S.P.R., N.W., A.K., and F.L. planned the experiments, analyzed the data, and wrote the article.

ACKNOWLEDGMENTS

This work was supported by grants from the Swedish Research Council, Ragnar Söderberg Foundation, Ming Wai Lau Center for Reparative Medicine, Center for Innovative Medicine, Wallenberg Academy Fellow, Stockholm County Council (ALF project), Karolinska Institute, Crown Princess Margareta's Foundation for the Visually Impaired, Strategic Research Area (SRA) Stem Cells and Regenerative Medicine, Ragnar Söderberg Foundation, ARMEC Lindeberg Foundation, the Ulla och Ingemar Dahlberg Foundation, the King Gustav V and Queen Victoria Foundation, and the Cronqvist Foundation. This study was performed at the Live Cell Imaging unit/Nikon Center of Excellence, BioNut, KI, supported by Knut and Alice Wallenberg Foundation, Swedish Research Council, Centre for Innovative Medicine and the Jonasson donation. Flow cytometry analysis and cell sorting were performed at the MedH Flow Cytometry core facility, supported by KI/SLL. Sequencing was performed at National Genomics Infrastructure in Stockholm at Science for Life Laboratory (funded by the Knut and Alice Wallenberg Foundation and the Swedish Research Council) with assistance from SNIC/Uppsala Multidisciplinary Center for Advanced Computational Science with massively parallel sequencing and access to the UPPMAX computational infrastructure. Authors report no financial interest related to this work.

Received: May 28, 2019
Revised: February 18, 2020
Accepted: February 19, 2020
Published: March 19, 2020

REFERENCES

Bae, S., Park, J., and Kim, J.S. (2014). Cas-OFFinder: a fast and versatile algorithm that searches for potential off-target sites of Cas9 RNA-guided endonucleases. Bioinformatics *30*, 1473–1475.

Bradley, J.A., Bolton, E.M., and Pedersen, R.A. (2002). Stem cell medicine encounters the immune system. Nat. Rev. Immunol. *2*, 859–871.

Choudhary, P., Booth, H., Gutteridge, A., Surmacz, B., Louca, I., Steer, J., Kerby, J., and Whiting, P.J. (2017). Directing differentiation of pluripotent stem cells toward retinal pigment epithelium lineage. Stem Cells Transl. Med. *6*, 490–501.

da Cruz, L., Fynes, K., Georgiadis, O., Kerby, J., Luo, Y.H., Ahmado, A., Vernon, A., Daniels, J.T., Nommiste, B., Hasan, S.M., et al. (2018). Phase 1 clinical study of an embryonic stem cell-derived retinal pigment epithelium patch in age-related macular degeneration. Nat. Biotechnol. *36*, 328–337.

de Rham, C., and Villard, J. (2011). How to cross immunogenetic hurdles to human embryonic stem cell transplantation. Semin. Immunopathol. *33*, 525–534.

Deuse, T., Hu, X., Gravina, A., Wang, D., Tediashvili, G., De, C., Thayer, W.O., Wahl, A., Garcia, J.V., Reichenspurner, H., et al. (2019). Hypoimmunogenic derivatives of induced pluripotent stem cells evade immune rejection in fully immunocompetent allogeneic recipients. Nat. Biotechnol. *37*, 252–258.

Diniz, B., Thomas, P., Thomas, B., Ribeiro, R., Hu, Y., Brant, R., Ahuja, A., Zhu, D., Liu, L., Koss, M., et al. (2013). Subretinal implantation of retinal pigment epithelial cells derived from human embryonic stem cells: improved survival when implanted as a monolayer. Invest. Ophthalmol. Vis. Sci. *54*, 5087–5096.

Finberg, R.W., White, W., and Nicholson-Weller, A. (1992). Decay-accelerating factor expression on either effector or target cells inhibits cytotoxicity by human natural killer cells. J. Immunol. *149*, 2055–2060.

Fox, A., Mountford, J., Braakhuis, A., and Harrison, L.C. (2001). Innate and adaptive immune responses to nonvascular xenografts: evidence that macrophages are direct effectors of xenograft rejection. J. Immunol. *166*, 2133–2140.

Gehrs, K.M., Anderson, D.H., Johnson, L.V., and Hageman, G.S. (2006). Age-related macular degeneration—emerging pathogenetic and therapeutic concepts. Ann. Med. *38*, 450–471.

Gornalusse, G.G., Hirata, R.K., Funk, S.E., Riolobos, L., Lopes, V.S., Manske, G., Prunkard, D., Colunga, A.G., Hanafi, L.A., Clegg, D.O., et al. (2017). HLA-E-expressing pluripotent stem cells escape allogeneic responses and lysis by NK cells. Nat. Biotechnol. *35*, 765–772.

Hawthorne, W.J., Lew, A.M., and Thomas, H.E. (2016). Genetic strategies to bring islet xenotransplantation to the clinic. Curr. Opin. Organ Transpl. *21*, 476–483.

Heigwer, F., Kerr, G., and Boutros, M. (2014). E-CRISP: fast CRISPR target site identification. Nat. Methods *11*, 122–123.

Hongisto, H., Ilmarinen, T., Vattulainen, M., Mikhailova, A., and Skottman, H. (2017). Xeno- and feeder-free differentiation of human pluripotent stem cells to two distinct ocular epithelial cell types using simple modifications of one method. Stem Cell Res. Ther. *8*, 291.

Idelson, M., Alper, R., Obolensky, A., Ben-Shushan, E., Hemo, I., Yachimovich-Cohen, N., Khaner, H., Smith, Y., Wiser, O., Gropp, M., et al. (2009). Directed differentiation of human embryonic stem cells into functional retinal pigment epithelium cells. Cell Stem Cell *5*, 396–408.

Ilmarinen, T., Hiidenmaa, H., Koobi, P., Nymark, S., Sorkio, A., Wang, J.H., Stanzel, B.V., Thieltges, F., Alajuuma, P., Oksala, O., et al. (2015). Ultrathin polyimide membrane as cell carrier for subretinal transplantation of human embryonic stem cell derived retinal pigment epithelium. PLoS One *10*, e0143669.

Ilmarinen, T., Thieltges, F., Hongisto, H., Juuti-Uusitalo, K., Koistinen, A., Kaarniranta, K., Brinken, R., Braun, N., Holz, F.G., Skottman, H., et al. (2018). Survival and functionality of xeno-free

human embryonic stem cell-derived retinal pigment epithelial cells on polyester substrate after transplantation in rabbits. Acta Ophthalmol. *97*, e688–e699.

Jonas, J.B., Cheung, C.M.G., and Panda-Jonas, S. (2017). Updates on the epidemiology of age-related macular degeneration. Asia Pac. J. Ophthalmol. (Phila) *6*, 493–497.

Kamao, H., Mandai, M., Okamoto, S., Sakai, N., Suga, A., Sugita, S., Kiryu, J., and Takahashi, M. (2014). Characterization of human induced pluripotent stem cell-derived retinal pigment epithelium cell sheets aiming for clinical application. Stem Cell Rep. *2*, 205–218.

Karre, K., Ljunggren, H.G., Piontek, G., and Kiessling, R. (1986). Selective rejection of H-2-deficient lymphoma variants suggests alternative immune defence strategy. Nature *319*, 675–678.

Kashani, A.H., Lebkowski, J.S., Rahhal, F.M., Avery, R.L., Salehi-Had, H., Dang, W., Lin, C.M., Mitra, D., Zhu, D., Thomas, B.B., et al. (2018). A bioengineered retinal pigment epithelial monolayer for advanced, dry age-related macular degeneration. Sci. Transl. Med. *10*. https://doi.org/10.1126/scitranslmed.aao4097.

Klimanskaya, I., Hipp, J., Rezai, K.A., West, M., Atala, A., and Lanza, R. (2004). Derivation and comparative assessment of retinal pigment epithelium from human embryonic stem cells using transcriptomics. Cloning Stem Cells *6*, 217–245.

Lane, A., Philip, L.R., Ruban, L., Fynes, K., Smart, M., Carr, A., Mason, C., and Coffey, P. (2014). Engineering efficient retinal pigment epithelium differentiation from human pluripotent stem cells. Stem Cells Transl. Med. *3*, 1295–1304.

Liang, Q., Monetti, C., Shutova, M.V., Neely, E.J., Hacibekiroglu, S., Yang, H., Kim, C., Zhang, P., Li, C., Nagy, K., et al. (2018). Linking a cell-division gene and a suicide gene to define and improve cell therapy safety. Nature *563*, 701–704.

Ljunggren, H.G., and Karre, K. (1990). In search of the 'missing self': MHC molecules and NK cell recognition. Immunol. Today *11*, 237–244.

Lu, B., Malcuit, C., Wang, S., Girman, S., Francis, P., Lemieux, L., Lanza, R., and Lund, R. (2009). Long-term safety and function of RPE from human embryonic stem cells in preclinical models of macular degeneration. Stem Cells *27*, 2126–2135.

Lund, R.D., Wang, S., Klimanskaya, I., Holmes, T., Ramos-Kelsey, R., Lu, B., Girman, S., Bischoff, N., Sauve, Y., and Lanza, R. (2006). Human embryonic stem cell-derived cells rescue visual function in dystrophic RCS rats. Cloning Stem Cells *8*, 189–199.

Mandai, M., Watanabe, A., Kurimoto, Y., Hirami, Y., Morinaga, C., Daimon, T., Fujihara, M., Akimaru, H., Sakai, N., Shibata, Y., et al. (2017). Autologous induced stem-cell-derived retinal cells for macular degeneration. N. Engl. J. Med. *376*, 1038–1046.

Marcenaro, E., Augugliaro, R., Falco, M., Castriconi, R., Parolini, S., Sivori, S., Romeo, E., Millo, R., Moretta, L., Bottino, C., et al. (2003). CD59 is physically and functionally associated with natural cytotoxicity receptors and activates human NK cell-mediated cytotoxicity. Eur. J. Immunol. *33*, 3367–3376.

Maruotti, J., Sripathi, S.R., Bharti, K., Fuller, J., Wahlin, K.J., Ranganathan, V., Sluch, V.M., Berlinicke, C.A., Davis, J., Kim, C., et al. (2015). Small-molecule-directed, efficient generation of retinal pigment epithelium from human pluripotent stem cells. Proc. Natl. Acad. Sci. U S A *112*, 10950–10955.

Masternak, K., Muhlethaler-Mottet, A., Villard, J., Zufferey, M., Steimle, V., and Reith, W. (2000). CIITA is a transcriptional coactivator that is recruited to MHC class II promoters by multiple synergistic interactions with an enhanceosome complex. Genes Dev. *14*, 1156–1166.

McGill, T.J., Stoddard, J., Renner, L.M., Messaoudi, I., Bharti, K., Mitalipov, S., Lauer, A., Wilson, D.J., and Neuringer, M. (2018). Allogeneic iPSC-derived RPE cell graft failure following transplantation into the subretinal space in nonhuman primates. Invest. Ophthalmol. Vis. Sci. *59*, 1374–1383.

Nathenson, S.G., Uehara, H., Ewenstein, B.M., Kindt, T.J., and Coligan, J.E. (1981). Primary structural: analysis of the transplantation antigens of the murine H-2 major histocompatibility complex. Annu. Rev. Biochem. *50*, 1025–1052.

Osakada, F., Jin, Z.B., Hirami, Y., Ikeda, H., Danjyo, T., Watanabe, K., Sasai, Y., and Takahashi, M. (2009). In vitro differentiation of retinal cells from human pluripotent stem cells by small-molecule induction. J. Cell Sci. *122*, 3169–3179.

Pegram, H.J., Andrews, D.M., Smyth, M.J., Darcy, P.K., and Kershaw, M.H. (2011). Activating and inhibitory receptors of natural killer cells. Immunol. Cell Biol. *89*, 216–224.

Pennington, B.O., Clegg, D.O., Melkoumian, Z.K., and Hikita, S.T. (2015). Defined culture of human embryonic stem cells and xeno-free derivation of retinal pigmented epithelial cells on a novel, synthetic substrate. Stem Cells Transl. Med. *4*, 165–177.

Petrus-Reurer, S., Bartuma, H., Aronsson, M., Westman, S., Lanner, F., Andre, H., and Kvanta, A. (2017). Integration of subretinal suspension transplants of human embryonic stem cell-derived retinal pigment epithelial cells in a large-eyed model of geographic atrophy. Invest. Ophthalmol. Vis. Sci. *58*, 1314–1322.

Plaza Reyes, A., Petrus-Reurer, S., Antonsson, L., Stenfelt, S., Bartuma, H., Panula, S., Mader, T., Douagi, I., Andre, H., Hovatta, O., et al. (2016). Xeno-free and defined human embryonic stem cell-derived retinal pigment epithelial cells functionally integrate in a large-eyed preclinical model. Stem Cell Rep. *6*, 9–17.

Rodin, S., Antonsson, L., Hovatta, O., and Tryggvason, K. (2014a). Monolayer culturing and cloning of human pluripotent stem cells on laminin-521-based matrices under xeno-free and chemically defined conditions. Nat. Protoc. *9*, 2354–2368.

Rodin, S., Antonsson, L., Niaudet, C., Simonson, O.E., Salmela, E., Hansson, E.M., Domogatskaya, A., Xiao, Z., Damdimopoulou, P., Sheikhi, M., et al. (2014b). Clonal culturing of human embryonic stem cells on laminin-521/E-cadherin matrix in defined and xeno-free environment. Nat. Commun. *5*, 3195.

Schwartz, S.D., Tan, G., Hosseini, H., and Nagiel, A. (2016). Subretinal transplantation of embryonic stem cell-derived retinal pigment epithelium for the treatment of macular degeneration: an assessment at 4 years. Invest. Ophthalmol. Vis. Sci. *57*, ORSFc1-9.

Sharma, R., Khristov, V., Rising, A., Jha, B.S., Dejene, R., Hotaling, N., Li, Y., Stoddard, J., Stankewicz, C., Wan, Q., et al. (2019). Clinical-grade stem cell-derived retinal pigment epithelium patch

rescues retinal degeneration in rodents and pigs. Sci. Transl. Med. *11*. https://doi.org/10.1126/scitranslmed.aat5580.

Sohn, E.H., Jiao, C., Kaalberg, E., Cranston, C., Mullins, R.F., Stone, E.M., and Tucker, B.A. (2015). Allogenic iPSC-derived RPE cell transplants induce immune response in pigs: a pilot study. Sci. Rep. *5*, 11791.

Song, W.K., Park, K.M., Kim, H.J., Lee, J.H., Choi, J., Chong, S.Y., Shim, S.H., Del Priore, L.V., and Lanza, R. (2015). Treatment of macular degeneration using embryonic stem cell-derived retinal pigment epithelium: preliminary results in Asian patients. Stem Cell Rep. *4*, 860–872.

Stanzel, B.V., Liu, Z., Somboonthanakij, S., Wongsawad, W., Brinken, R., Eter, N., Corneo, B., Holz, F.G., Temple, S., Stern, J.H., et al. (2014). Human RPE stem cells grown into polarized RPE monolayers on a polyester matrix are maintained after grafting into rabbit subretinal space. Stem Cell Rep. *2*, 64–77.

Sugita, S., Iwasaki, Y., Makabe, K., Kamao, H., Mandai, M., Shiina, T., Ogasawara, K., Hirami, Y., Kurimoto, Y., and Takahashi, M. (2016a). Successful transplantation of retinal pigment epithelial cells from MHC homozygote iPSCs in MHC-matched models. Stem Cell Rep. *7*, 635–648.

Sugita, S., Iwasaki, Y., Makabe, K., Kimura, T., Futagami, T., Suegami, S., and Takahashi, M. (2016b). Lack of T cell response to iPSC-derived retinal pigment epithelial cells from HLA homozygous donors. Stem Cell Rep. *7*, 619–634.

Sugita, S., Makabe, K., Fujii, S., Iwasaki, Y., Kamao, H., Shiina, T., Ogasawara, K., and Takahashi, M. (2017). Detection of retinal pigment epithelium-specific antibody in iPSC-derived retinal pigment epithelium transplantation models. Stem Cell Rep. *9*, 1501–1515.

Sugita, S., Makabe, K., Iwasaki, Y., Fujii, S., and Takahashi, M. (2018). Natural killer cell inhibition by HLA-E molecules on induced pluripotent stem cell-derived retinal pigment epithelial cells. Invest. Ophthalmol. Vis. Sci. *59*, 1719–1731.

Taylor, C.J., Peacock, S., Chaudhry, A.N., Bradley, J.A., and Bolton, E.M. (2012). Generating an iPSC bank for HLA-matched tissue transplantation based on known donor and recipient HLA types. Cell Stem Cell *11*, 147–152.

Vaajasaari, H., Ilmarinen, T., Juuti-Uusitalo, K., Rajala, K., Onnela, N., Narkilahti, S., Suuronen, R., Hyttinen, J., Uusitalo, H., and Skottman, H. (2011). Toward the defined and xeno-free differentiation of functional human pluripotent stem cell-derived retinal pigment epithelial cells. Mol. Vis. *17*, 558–575.

Vugler, A., Carr, A.J., Lawrence, J., Chen, L.L., Burrell, K., Wright, A., Lundh, P., Semo, M., Ahmado, A., Gias, C., et al. (2008). Elucidating the phenomenon of HESC-derived RPE: anatomy of cell genesis, expansion and retinal transplantation. Exp. Neurol. *214*, 347–361.

Xu, H., Wang, B., Ono, M., Kagita, A., Fujii, K., Sasakawa, N., Ueda, T., Gee, P., Nishikawa, M., Nomura, M., et al. (2019). Targeted disruption of HLA genes via CRISPR-Cas9 generates iPSCs with enhanced immune compatibility. Cell Stem Cell *24*, 566–578.e7.

Zhang, X., and Bok, D. (1998). Transplantation of retinal pigment epithelial cells and immune response in the subretinal space. Invest. Ophthalmol. Vis. Sci. *39*, 1021–1027.

Stem Cell Reports, Volume *14*

Supplemental Information

Generation of Retinal Pigment Epithelial Cells Derived from Human Embryonic Stem Cells Lacking Human Leukocyte Antigen Class I and II

Sandra Petrus-Reurer, Nerges Winblad, Pankaj Kumar, Laia Gorchs, Michael Chrobok, Arnika Kathleen Wagner, Hammurabi Bartuma, Emma Lardner, Monica Aronsson, Álvaro Plaza Reyes, Helder André, Evren Alici, Helen Kaipe, Anders Kvanta, and Fredrik Lanner

SUPPLEMENTAL ITEMS

Generation of retinal pigment epithelial cells derived from human embryonic stem cells lacking human leukocyte antigen class I and II

Petrus-Reurer et al.

Supplemental Figures 1 – 5

Supplemental Video 1

Supplemental Tables 1 – 4

Experimental Procedures

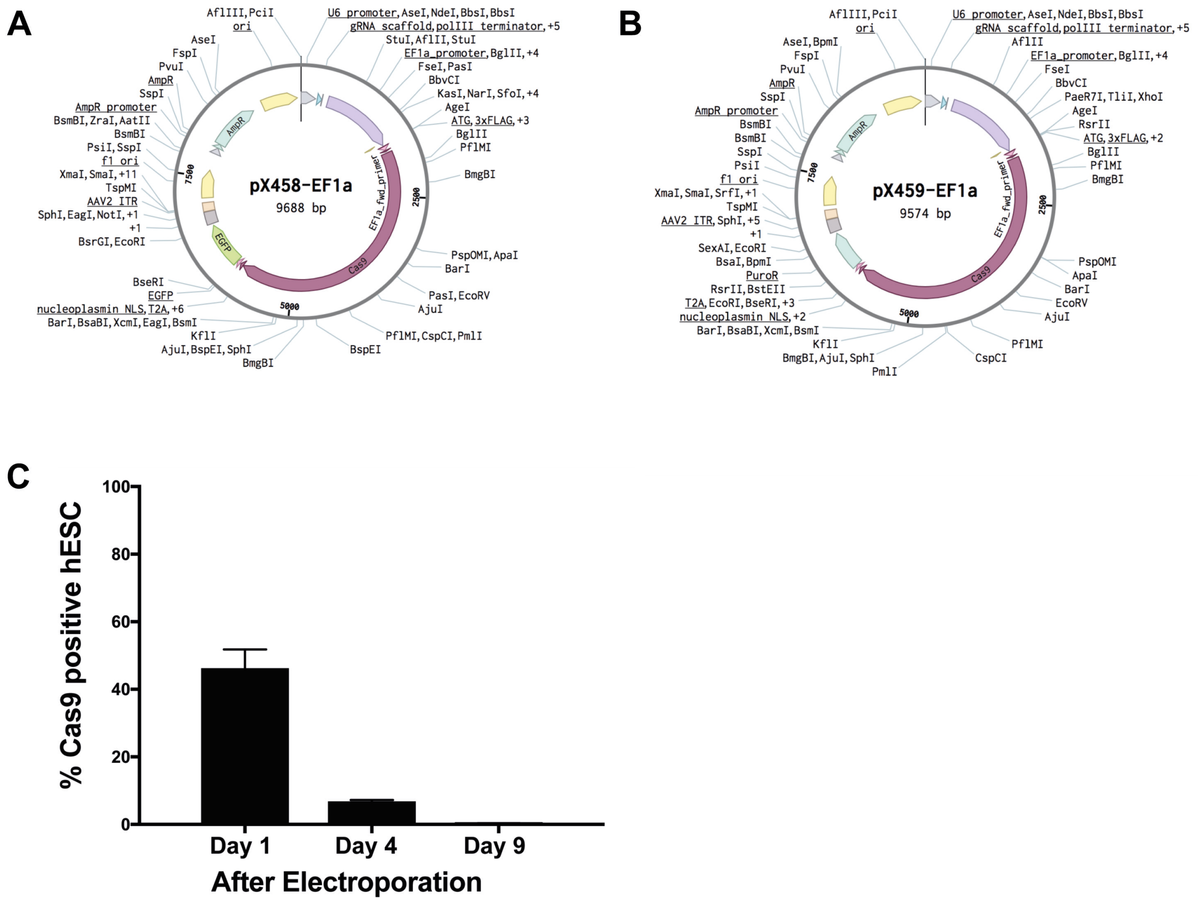

FIGURE S1. Modified CRISPR/Cas9 plasmids and evaluation of persistence of Cas9 in cells after electroporation. Related to Figure 1.

(A) Plasmid map of the final construct of pX458 containing the EF1α promoter. This plasmid was used during sgRNA screening. (B) Plasmid map of the final construct of pX459 containing the EF1α promoter. This plasmid was used in hESCs. Both plasmid maps (A, B) were generated using Benchling [Biology Software] (2016) and retrieved from https://benchling.com. (C) Bar graph showing percentage of cells still expressing Cas9 after one, four or nine days after electroporation shown by flow cytometry.

Bars represent mean±SEM from 3 independent experiments.

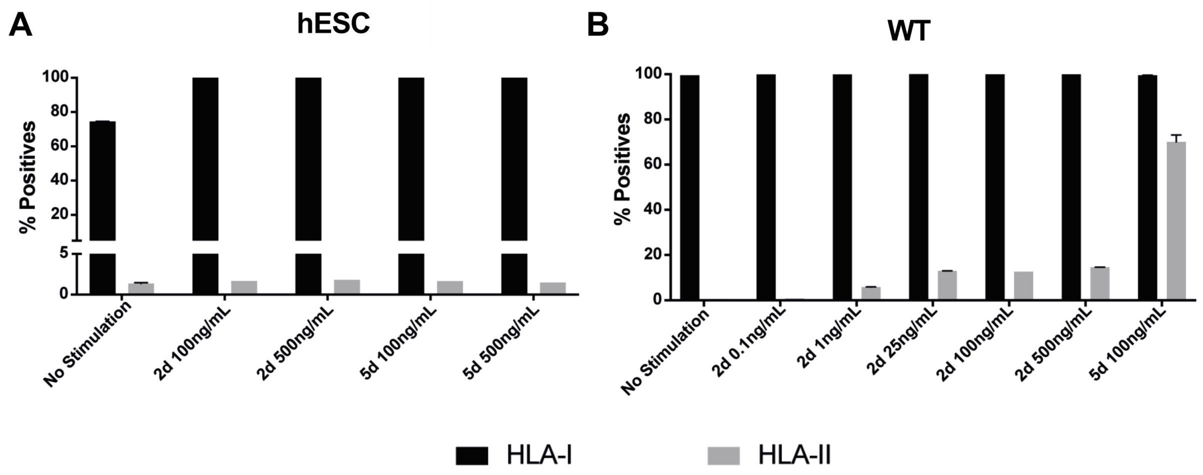

FIGURE S2. IFN-γ stimulation titration in hESC and hESC-RPE. Related to Figure 2.

(A) Bar graphs showing the percentage of HLA-I and HLA-II positive hESC cells measured by flow cytometry in different stimulatory conditions. (B) Bar graphs showing the percentage of HLA-I and HLA-II positive hESC-RPE cells measured by flow cytometry in different IFN-γ stimulatory conditions.

Bars represent mean±SEM from 3 independent experiments.

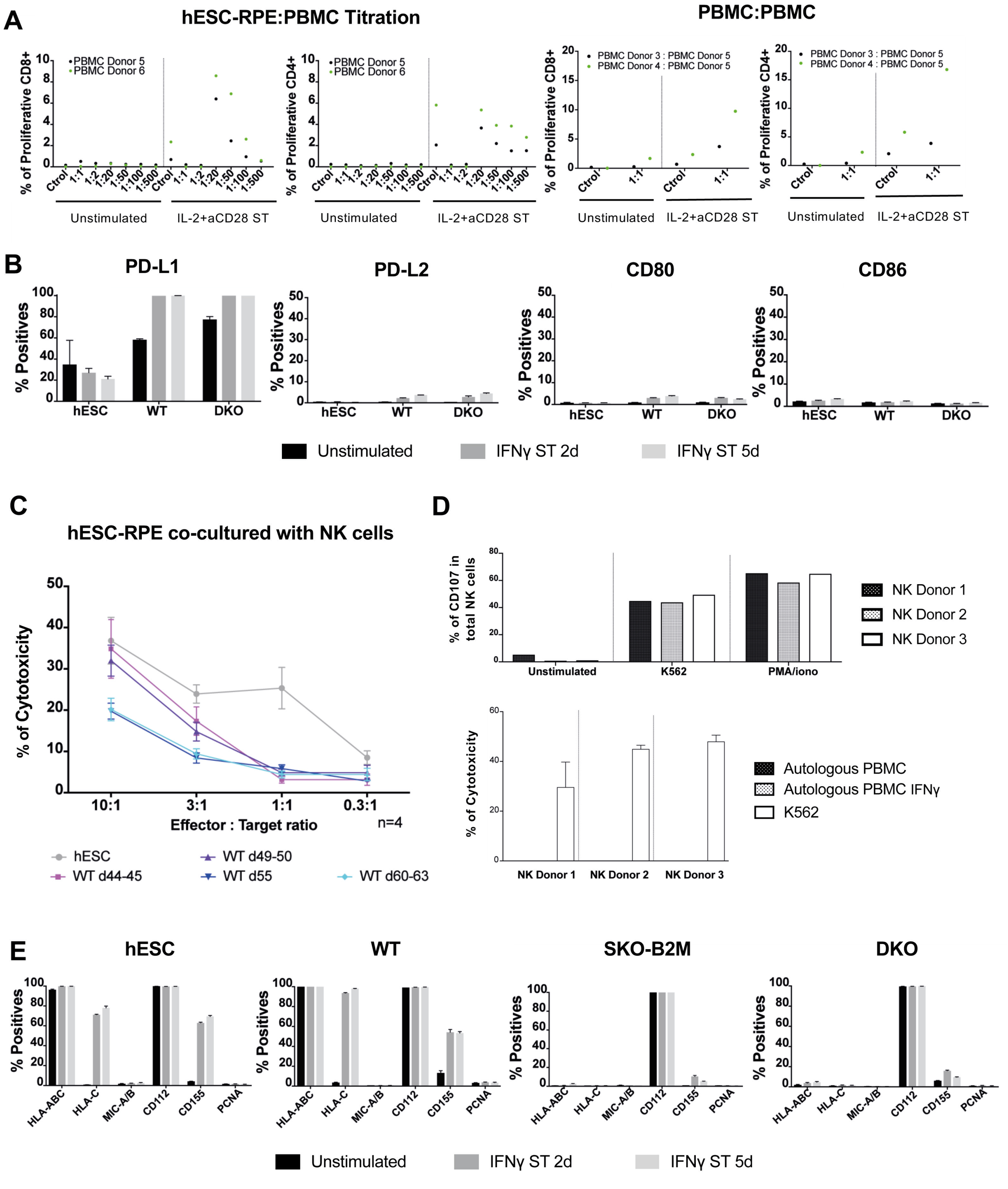
A
hESC-RPE:PBMC Titration
PBMC:PBMC
% of Proliferative CD8+
% of Proliferative CD4+
PBMC Donor 5
PBMC Donor 6
PBMC Donor 3 : PBMC Donor 5
PBMC Donor 4 : PBMC Donor 5
Ctrol
1:1
1:2
1:20
1:50
1:100
1:500
Unstimulated
IL-2+aCD28 ST
B
PD-L1
PD-L2
CD80
CD86
% Positives
hESC
WT
DKO
Unstimulated
IFNγ ST 2d
IFNγ ST 5d
C
hESC-RPE co-cultured with NK cells
% of Cytotoxicity
10:1
3:1
1:1
0.3:1
Effector : Target ratio
n=4
hESC
WT d44-45
WT d49-50
WT d55
WT d60-63
D
% of CD107 in total NK cells
Unstimulated
K562
PMA/iono
NK Donor 1
NK Donor 2
NK Donor 3
% of Cytotoxicity
Autologous PBMC
Autologous PBMC IFNγ
K562
E
hESC
WT
SKO-B2M
DKO
% Positives
HLA-ABC
HLA-C
MIC-A/B
CD112
CD155
PCNA
Unstimulated
IFNγ ST 2d
IFNγ ST 5d

FIGURE S3. *In vitro* immunogenicity assessment of WT, SKO-B2M and DKO hESC-RPE. Related to Figure 3.

(A) Bar graph showing the percentage of proliferative $CD8^+$ or $CD4^+$ cells upon 5 days co-culture of PBMCs from 2 different donors with WT cells at several hESC-RPE:PBMC ratios (1:1; 1:2; 1:20; 1:50; 1:100; 1:500) without (Unstimulated) or with the presence of IL-2+aCD28 stimulation (and 2 days IFN-γ 100 ng/mL hESC-RPE pre-stimulation) (hESC-RPE:PBMC Titration, left panel). PBMC:PBMC (mixed lymphocyte reaction, as T-cell proliferation control, right panel) shows the percentage of proliferative $CD8^+$ or $CD4^+$ cells upon 5 days co-culture of PBMCs from different donors at 1:1 ratio without (Unstimulated) or with the presence of IL-2+aCD28 stimulation. PBMCs only were used as negative controls (Ctrol). (B) Bar graphs showing the percentage of positive cells expressing co-inhibitory T-cell ligands PD-L1 and PD-L2, and co-stimulatory molecules CD80 and CD86 measured by flow cytometry in hESC, WT and SKO-B2M cells unstimulated or stimulated 2 or 5 days with IFN-γ 100 ng/mL. (C) Graph showing the percentage of cytotoxicity of hESC and WT cells at different maturity stages/days (target) measured by chromium release of the killed cells by the freshly isolated and overnight IL-2 stimulated NK cells (effector) of 4 different donors. Several target:effector cell ratios were tested (10:1; 3:1; 1:1; 0.3:1). (D) Bar graphs showing the percentage of NK degranulation shown by CD107 positive expression in total NK cells when cultured without target cells (unstimulated), with K562 cells or with phorbol-myristate-acetate/Ionomycin (PMA/Iono) from 3 different donors (upper panel); and the percentage of cytotoxicity of autologous PBMCs, 100 ng/mL IFN-γ over-night stimulated autologous PBMCs or K562 cells measured by chromium release of the killed cells by the freshly isolated and overnight IL-2 stimulated NK cells of 3 different donors (lower panel). (E) Bar graphs showing the percentage of positive cells expressing of NK cell ligands HLA-ABC, HLA-C, MIC-A/B, CD112, CD155 and PCNA measured by flow cytometry in hESC, WT, SKO-B2M and DKO cells unstimulated or stimulated 2 or 5 days with IFN-γ 100ng/mL.

Bars represent mean±SEM from 3 (B, D, E) or 4 (C) independent experiments.

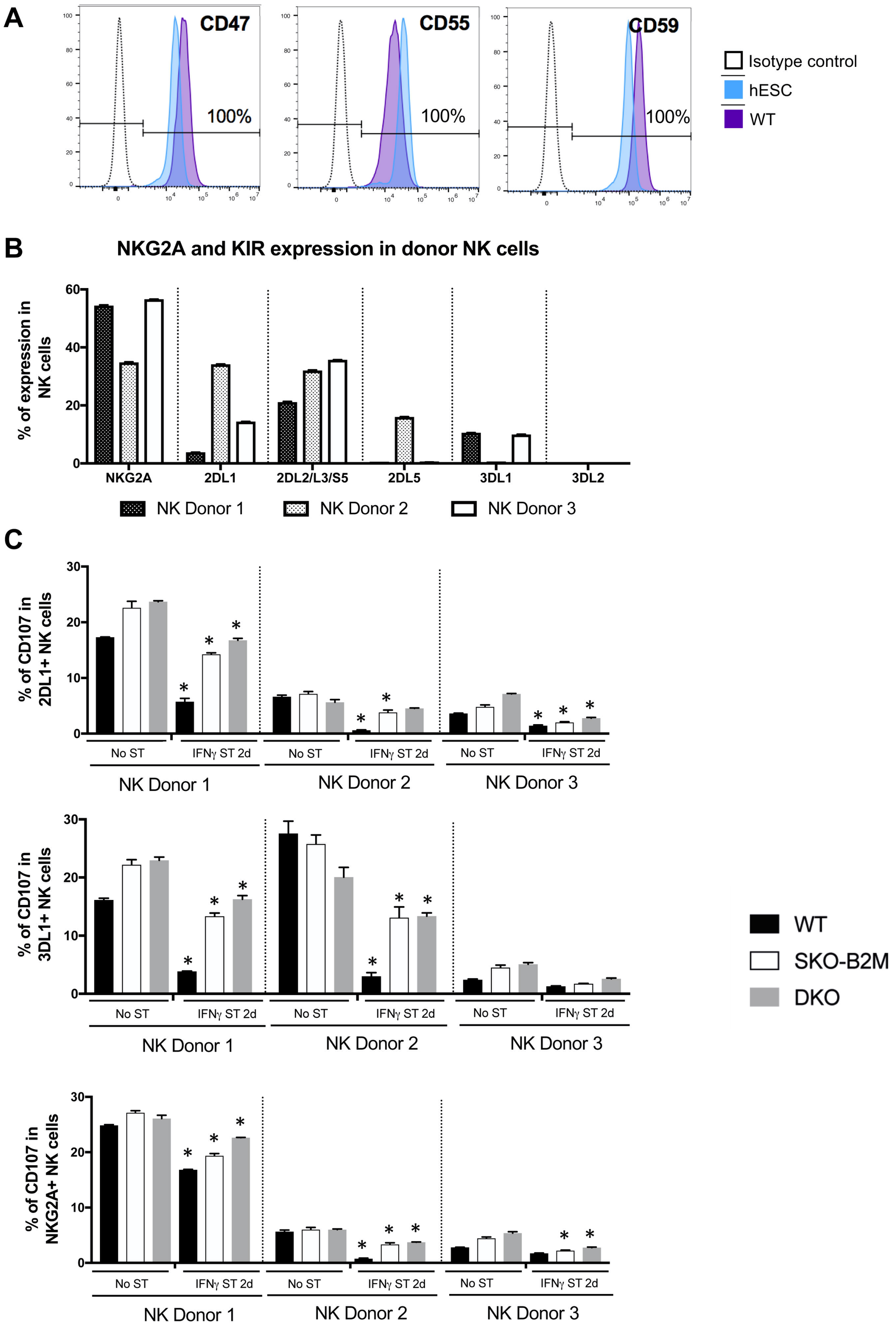
A
CD47
CD55
CD59
100%
100%
100%
Isotype control
hESC
WT
B
NKG2A and KIR expression in donor NK cells
% of expression in NK cells
NKG2A
2DL1
2DL2/L3/S5
2DL5
3DL1
3DL2
NK Donor 1
NK Donor 2
NK Donor 3
C
% of CD107 in 2DL1+ NK cells
% of CD107 in 3DL1+ NK cells
% of CD107 in NKG2A+ NK cells
No ST
IFNγ ST 2d
NK Donor 1
NK Donor 2
NK Donor 3
WT
SKO-B2M
DKO

FIGURE S4. ***In vitro*** **immunogenicity assessment of WT, SKO-B2M and DKO hESC-RPE. Related to Figure 3.**

(A) Representative flow cytometry histogram showing the percentage of hESC and WT cells expressing extracellular CD47, CD55 and CD59. Dotted line histogram shows respective mIgG isotype control (negative control used for gating). (B) Bar graphs showing the percentage of expression of NKG2A and several different KIR receptors (2DL1, 2DL2/L3/S5, 2DL5, 3DL1 and 3DL2) from 3 different donors. hESC-RPE haplotype: HLA-A*32/68, HLA-B*35/38, HLA-C*04/07, HLA-DQA1*03/04, HLA-DQB1*03/03, HLA-DRB1*04/12 corresponding to KIRs 2DL1 (HLA-C*04 included in C1 epitope), 2DL2 (HLA-C*07 included in C1 epitope), 3DL1 (HLA-A*32, HLA-B*38 included in Bw4 epitope) and 3DL2 (HLA-A*68 included in HLA-A3 epitope) (HLA ligand for 2DL5 remains undefined). (C) Bar graphs showing the percentage of NK degranulation shown by CD107 positive expression in 2DL1+, 3DL1+ or NKG2A+ NK-cells when co-cultured with WT, SKO-B2M and DKO cells unstimulated or 2 days IFN-γ 100 ng/mL pre-stimulation from 3 different donors.

Bars represent mean±SEM from 3 independent experiments.

*P-values: C (compared to respective No ST cell line per donor) <0.0001.

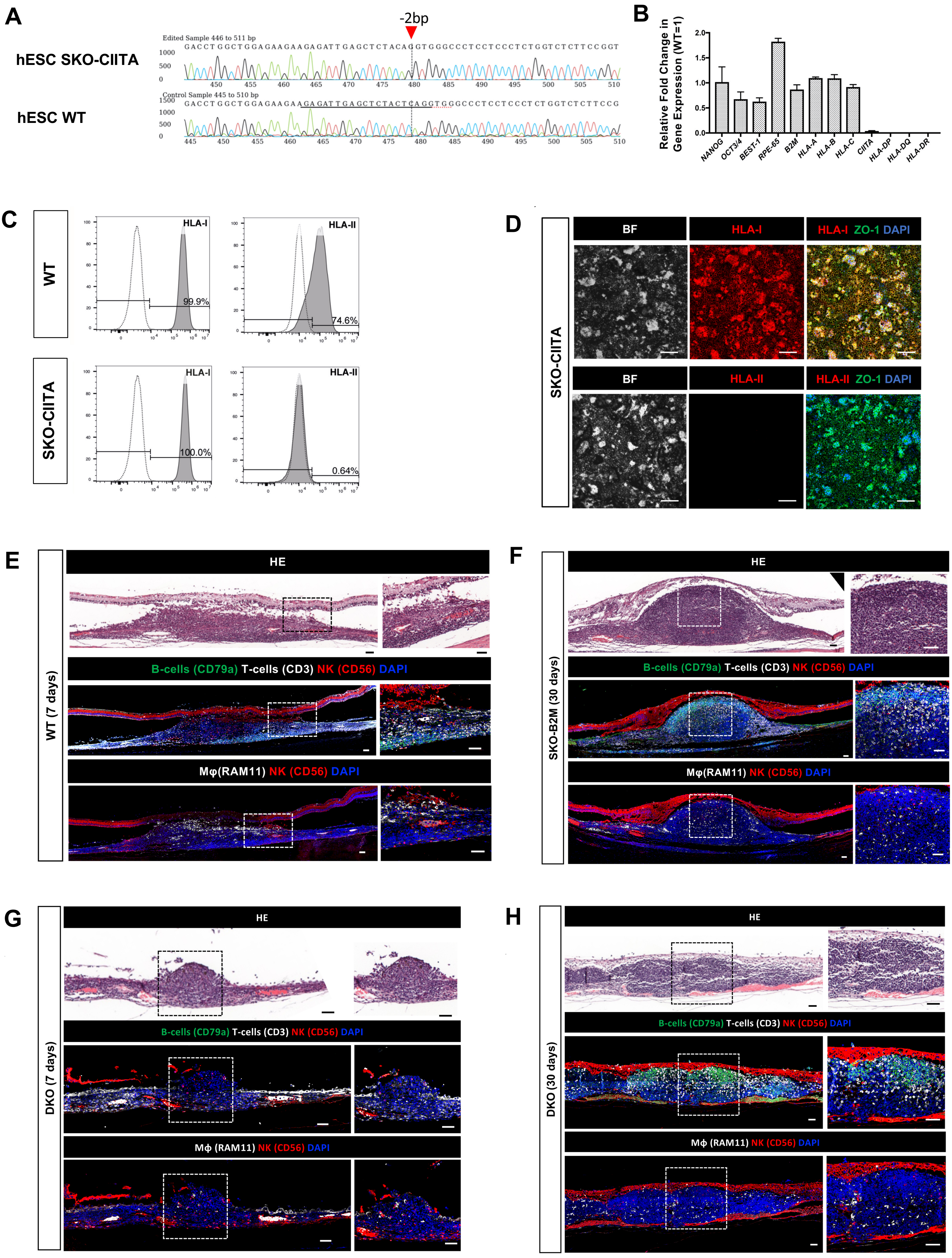
A
B
hESC SKO-CIITA
hESC WT
-2bp
Edited Sample 446 to 511 bp
Control Sample 445 to 510 bp
Relative Fold Change in Gene Expression (WT=1)
NANOG
OCT3/4
BEST-1
RPE-65
B2M
HLA-A
HLA-B
HLA-C
CIITA
HLA-DP
HLA-DQ
HLA-DR
C
WT
SKO-CIITA
HLA-I
HLA-II
99.9%
74.6%
100.0%
0.64%
D
SKO-CIITA
BF
HLA-I
HLA-I ZO-1 DAPI
HLA-II
HLA-II ZO-1 DAPI
E
WT (7 days)
HE
B-cells (CD79a) T-cells (CD3) NK (CD56) DAPI
Mφ(RAM11) NK (CD56) DAPI
F
SKO-B2M (30 days)
G
DKO (7 days)
H
DKO (30 days)

FIGURE S5. Characterization of SKO-CIITA hESC-RPE cells and assessment of WT, SKO-B2M and DKO hESC-RPE upon subretinal injections in the xenograft model. Related to Figure 5.

(A) Indel analysis obtained by Sanger sequencing of hESC SKO-CIITA. (B) Representative flow cytometry histogram showing the percentage of WT and SKO-CIITA cells expressing extracellular HLA-I and HLA-II. Dotted line histogram shows HLA-I FMO (negative control used for gating). (C) Immunofluorescence images of WT and SKO-CIITA cells showing HLA-I, HLA-II and ZO-1 expression. (D) Gene expression analysis of pluripotent, RPE and HLA related genes in the targeted hESC-RPE. Values are normalized to *GAPDH* and displayed as relative to WT. (E) HE and immunofluorescence images 7 days after subretinal injection of WT cells showing the expression of rabbit $CD3^+$ T-cells, $CD56^+$ NK cells, $CD79a^+$ B-cells and $RAM11^+$ macrophages. Dashed square indicates the zoom-in shown on the right side. (F) HE and immunofluorescence images 30 days after subretinal injection of SKO-B2M cells showing the expression of rabbit $CD3^+$ T-cells, $CD56^+$ NK cells, $CD79a^+$ B-cells and $RAM11^+$ macrophages. Dashed square indicates the zoom-in shown on the right side. (G) HE and immunofluorescence images 7 days after subretinal injection of DKO cells showing the expression of rabbit $CD3^+$ T-cells, $CD56^+$ NK cells, $CD79a^+$ B-cells and $RAM11^+$ macrophages. Dashed square indicates the zoom-in shown on the right side. (H) HE and immunofluorescence images 30 days after subretinal injection of DKO cells showing the expression of rabbit $CD3^+$ T-cells, $CD56^+$ NK cells, $CD79a^+$ B-cells and $RAM11^+$ macrophages. Dashed squares indicate the zoom-ins images shown on the right side.

Bars represent mean±SEM from 3 independent experiments.

Scale bars: (D) = 100 μm; (E-H) = 50 μm.

VIDEO S1. Related to Figure 4.

Movie showing 72 consecutive SD-OCT scans 7 weeks after injection of WT hESC-RPE cells in the subretinal space of the rabbit eye. Green arrow indicates the SD-OCT scan plane.

TABLE S1. sgRNA sequences for gene targets B2M and CIITA. Related to Experimental Procedures.

B2M-1	5’-GGCCGAGATGTCTCGCTCCG-3’
B2M-6	5’-GAGTAGCGCGAGCACAGCTA-3’
B2M-7	5’-CAGTAAGTCAACTTCAATGT-3’
CIITA-4	5’-AGGTGATGAAGAGACCAGGG-3’
CIITA-5	5’-GAGATTGAGCTCTACTCAGG-3’
CIITA-9	5’-CATCGCTGTTAAGAAGCTCC-3’

TABLE S2. Primers utilized for amplification of gDNA through PCR and for SURVEYOR Nuclease Assay. Related to Experimental Procedures.

B2M Forward primer 1	5’-GCACGCGTTTAATATAAGTGGAGGCG-3’
B2M Forward primer 2	5’-CTCCAGCCTGAAGTCCTAGAATGAGC-3’
B2M Forward primer 3	5’-GTGGCTTGTTGGGAAGGTGGAAG-3’
B2M Reverse primer 1	5’-TTTGGGACGAGCCTACCCGT-3’
B2M Reverse primer 3	5’-GCTGTGCATCAGTATCTCAGCAGGT-3’
CIITA Forward primer 2	5’-AATGTGCCAGCTCATGTGCCTG-3´
CIITA Forward primer 3	5’- CTCTCTGCAGATGGGGATGATCTCC-3’
CIITA Reverse primer 2	5’- TTGATGGTGTCTGTGTCGGGTTCT-3´
CIITA Reverse primer 4	5’- GCAGAAATGCGTATAGAAATGGAGGTGG-3’

TABLE S3. Sequence read number, mean coverage (sequencing depth) and Germline SNVs for hESC WT (HS980), hESC SKO-B2M and hESC DKO samples. Related to Figure 1.

Sample Name	Reads (paired-end)	Aligned reds (%) (both in pairs)	Coverage (mean)	Germline SNVs (SNPs & Indels)
hESC (HS980)	839393930	832313573 (99.16%)	39.27	4604767 (3794906 & 813807)
hESC SKO-B2M	687569526	672844498 (97.86%)	30.36	4754497 (3925443 & 833210)
hESC DKO	737021865	701549978 (95.19%)	31.04	4748188 (3887630 & 864627)

TABLE S4. Total homozygous deletions identified for hESC WT vs hESC SKO-B2M, and hESC SKO-B2M vs hESC DKO samples. Related to Figure 1.

See attached Table S4.xlsx file.

EXPERIMENTAL PROCEDURES, Related to Experimental Procedures.

Cell Culture

Human embryonic stem cell line HS980 was derived from supernumerary *in vitro* fertilized human embryos and cultured under xeno-free and defined conditions according to the previously described method (Rodin et al., 2014a; Rodin et al., 2014b) and under the approval of DNR 2011/745-31/3. The cells were maintained by clonal propagation on hrLN-521 10 µg/mL (Biolamina) in NutriStem hESC XF medium (Biological Industries, 05-100-1A), in a 5% CO_2/5% O_2 incubator and passaged enzymatically at a 1:10 ratio every 5-6 days.

For passaging, confluent cultures were washed twice with PBS without Ca^{2+} and Mg^{2+} (DPBS, ThermoFisher Scientific, 14190-094) and incubated for 5 min at 37°C, 5% CO_2/5% O_2 with TrypLE Select (ThermoFisher Scientific, 12563-011). The enzyme was then carefully removed and the cells were collected in fresh pre-warmed NutriStem hESC XF medium (Biological Industries, 05-100-1A) by gentle pipetting to obtain a single cell suspension. The cells were centrifuged at 300g for 4 min, the pellet resuspended in fresh prewarmed NutriStem hESC XF medium (Biological Industries, 05-100-1A) and cells plated on a freshly hrLN-521 (10 µg/mL, Biolamina) coated dish. Two days after passage the medium was replaced with fresh prewarmed NutriStem hESC XF medium (Biological Industries, 05-100-1A) and changed daily.

hESC-RPE 2D *In Vitro* Differentiation

Pluripotent stem cells were plated at a cell density of $2.4x10^4$ cells/cm^2 on 20 µg/mL hrLN-111 coated dishes (Biolamina) using NutriStem hESC XF medium (Biological Industries, 05-100-1A). Rho-kinase inhibitor (Millipore, Y-27632) at a concentration of 10 µM was added during the first 24h, while cells were kept at 37°C, 5% CO_2/5% O_2. After 24h, hESC medium was replaced with differentiation medium NutriStem hESC XF without bFGF and TGFβ (Biologicial Industries, 06-5100-01-1A) and cells were placed at 37°C, 5% CO_2. From day 6 after plating, 100 ng/mL of Activin A (R&D Systems, 338-AC-050) was added to the media. Cells were fed three times a week and kept for 5 weeks. Monolayers were then trypsinized using TrypLE Select (ThermoFisher Scientific, 12563-011) for 10 min at 37°C, 5% CO_2. The enzyme was carefully removed and the cells were collected in fresh pre-warmed NutriStem hESC XF medium without bFGF and TGFβ (Biologicial Industries, 06-5100-01-1A) by gentle pipetting to obtain a single cell suspension. The cells were centrifuged at 300g for 4 min, the pellet was resuspended, passed through a cell strainer (ø 40 µm, VWR, 732-2757) and cells were seeded on LN-coated dishes (hrLN-521 20 µg/mL, Biolamina) at a cell density of $7x10^4$ cells/cm^2. Re-plated cells were fed three times a week during the subsequent four weeks with the same differentiation medium referred above.

Promoter Exchange of pX459

The EF-1α promoter has been shown to be more stable in embryonic stem cells, which would ensure optimal expression of Cas9 and GFP or puromycin-resistance, respectively, from the plasmid (Liu et al., 2009) Firstly, the CMV promoter of pX458 (addgene no. 48138) and pX459 (addgene #62988) was exchanged for EF-1α (Fig S1A and S1B) by amplifying the latter off of pEF-GFP (addgene #11154) using forward and reverse primers 5'-AATTCTGCAGACAAATGGCTCTAGAGGTACGGTACCCGTGAGGCTCCGGTGCCC-3' and 5'-CGTGGTCCTTATAGTCCATGGTGGCACCGGACCGGTTCACGACACCTGAAATGGAAGAAAA-3' (Thermo Fisher Sicentific). The amplified fragment was ligated into BshTI (Thermo Fisher Sicentific, FD1464) and KpnI (Thermo Fisher Sicentific, FD0524) digested and FastAP (Thermo Fisher Sicentific, EF0652) treated pX459 using Gibson Assembly Master Mix (NEB, E2611S). The new plasmid constructs, henceforth referred to as pX459-EF-1α, were transformed into TOP10 bacteria (Thermo Fisher Sicentific, C404003) and plated onto ampicillin-containing LB agar plates (ThermoFisher Scientific, Q60120). Single colonies were picked and expanded in LB broth (ThermoFisher Scientific, 10855021) supplemented with 100 µg/mL ampicillin (ThermoFisher Scientific, 11593027) overnight, and plasmid DNA was extracted using QIAprep Spin Miniprep Kit (QIAGEN, 27104) according to the manufacturer's protocol.

gRNA Cloning into pX459-EF-1α

Digesting pX459-EF-1α using BpiI (ThermoFisher Scientific, FD1014) allowed for directional insertion of each gRNA. Prior to ligation, gRNA oligonucleotides (Table S1) were annealed in 1X Rapid ligation buffer (ThermoFisher Scientific, K1423) using a thermocycler set at 95°C for 5 min followed by 5°C/min ramp down to 25°C. The oligonucleotides were PNK (ThermoFisher Scientific, IVGN2304) treated according to the manufacturer's protocol. Annealed and PNK treated gRNA oligonucleotides were ligated into pX459- EF-1α using T4 DNA ligase (ThermoFisher Scientific, K1423) and the mixture was transformed into STBL3 bacteria (ThermoFisher Scientific, C737303) and plated on agar plates containing 100 µg/mL ampicillin. Single colonies were picked and expanded in LB broth with ampicillin (100 µg/mL) overnight and plasmid DNA was extracted using QIAprep Spin Miniprep Kit (QIAGEN, 27104) according to the manufacturer's protocol.

Guide Electroporation, Selection and Clonal Expansion

HEK293T cells (ThermoFisher Scientific, 293T Cells) were cultured in DMEM (ThermoFisher Scientific, 41965-039) supplemented with 10% FBS (ThermoFisher Scientific, 10082147), 0.1

mM NEAA (ThermoFisher Scientific; 11140-035), 6 mM GlutaMAXX (ThermoFisher Scientific, 35050-038) and 1 mM Sodium Pyruvate (ThermoFisher Scientific; 11360039) and dissociated using TrypLE Select (Gibco, 12563011). One hundred thousand cells were electroporated with 1 µg pX458_EF-1α-Cas9_U6-sgRNA using the NEON™ Transfection System (Thermo Fisher Sicentific, MPK5000) according to the manufacturer's protocol. Transfection success was estimated through GFP intensity from the pX458_EF-1α-Cas9_U6-sgRNA. The transfected cells were plated on 6-well plates and were allowed to recover until confluent before harvesting the cells for gDNA extraction and mutation detection.

Human embryonic stem cells (HS980) were cultured in Nutristem hESC XF medium (Biological Industries, 05-100-1A) as described previously until they reached 70–80% confluency, at which time they were dissociated using TrypLE Select (Gibco, 12563011) and prepared for NEON™ electroporation (ThermoFisher Scientific, MPK5000) according to the manufacturer's protocol. One hundred thousand cells were electroporated with pX459-EF-1α-Cas9-U6–B2M and then plated into a well on a six-well plate previously pre-coated overnight with 10 µg/mL hrLN-521 (Biolamina) in Dulbecco's Phosphate Buffered Saline containing calcium and magnesium (Gibco, 14040091). Twenty-four hours post-electroporation cells were selected using 0.5 µg/mL Puromycin (ThermoFisher Scientific, A1113803) for 24 hours after which fresh media was changed and the cells were left to recover. Bulk targeting efficiencies were determined using ICE analysis provided by Synthego (https://ice.synthego.com/#/) and were 80% and 84% for B2M and CIITA gRNAs, respectively. Once the cells were 70–80% confluent they were dissociated, diluted and plated at a concentration of two cells per well in a 96-well plate previously coated with 15 µg/mL hrLN-521 (Biolamina, LN521) and 1.7 µg/mL E-cadherin (R&D Systems, 8505-EC-050) overnight. Rho-kinase inhibitor (Millipore, Y-27632) at a concentration of 10µM was added during the first 24 hours. Single-cell clones were cultured in this format until confluent and were then dissociated and plated into two 48-wells per clones for further expansion. Cells in one out of the two wells were then used for genomic DNA extraction and Sanger sequencing of the region surrounding the edit using *B2M* primers F2 and R1 (Table S2) for PCR amplification. For hESC SKO-B2M, 20 single-cell clones were sequenced at the target site and all contained various indels. This procedure was repeated using the hESC SKO-B2M line starting from the editing step by electroporating one hundred thousand hESC SKO-B2M α-Cas9-U6–CIITA to generate hESC DKO. Amplification of the region surrounding the sgRNA-CIITA-5 edit site was done using *CIITA* primers F2 and R2 (Table S2). For hESC

DKO⁻, 42 single-cell clones were sequenced at the *CIITA* target site out of which 37 contained indels and five remained unedited.

Mutation Detection using SURVEYOR Nuclease Assay

Genomic DNA extraction was performed using QuickExtractTM DNA extraction solution (Lucigen, QE09050) and 1 µL of the extracted DNA was utilized for PCR amplification using Herculase II Fusion Enzyme (Agilent, 600677) and primers F1 and R1 for sgRNAs 1 and 2 located in exon 1 and primers F3 and R3 for sgRNA-3 located in exon 2 in the B2M locus, (Table S2). Primers F2 and R2 amplified a region around sgRNAs 5 and 9 and primers F3 and R4 amplified a region surrounding sgRNA 4 in the CIITA locus (Table S2). Mutation detection was performed using SURVEYOR Nuclease Assay (IDT, 706020) according to the manufacturer's protocol. The mismatch analysis was carried out according to the published protocol by Ran et al. (2013). Indel percentage was calculated according to $100*1-\sqrt{((1-((b+c))/((a+b+c))))}$, where a symbolizes the integrated intensity of the unedited DNA fragment, and b and c represent the intensities for each cleaved fragment (Ran et al., 2013).

Detection of Cas9 in Cells using Fluorescence Activated Cell Sorting (FACS)

HS980 hESCs were cultured and electroporated at nine, four and one days before analysis through FACS. Cells were selected for 24h with 0.5µg/ml puromycin (ThermoFisher Scientific, A1113803), except for day 1 samples that were not selected but collected for analysis. Cells were dissociated using TrypLE Select (Gibco, 12563011), centrifuged at 300g for 4 min and resuspended in DPBS (ThermoFisher Scientific, 14190-094). Samples were incubated with LIVE/DEAD fixable Violet Dead Cell Stain (Invitrogen, L34964) according to the manufacturer's protocol. Following LIVE/DEAD stain, fixation and permeabilization was carried out according to Cytofix/Cytoperm™ Fixation/Permeabilization Kit (BD Biosciences, 554714) for intracellular staining. Briefly, LIVE/DEAD stained samples were incubated with Fixation/Permeabilization solution for 20 minutes at 4°C and washed twice with 1 x BD Perm/Wash buffer. Samples were stained for intracellular Cas9 with an Alexa Fluor 647 conjugated Cas9 antibody (1:50, Cell Signaling Technologies, 48796) for 30 minutes at 4°C.Pellets were washed twice with 1 x BD Perm/Wash buffer and finally resuspended in 2% FBS (ThermoFisher Scientific, 10082147) and 1mM EDTA (Sigma, E7889) diluted in DPBS (ThermoFisher Scientific, 14190-094). Non-electroporated cells per time-point were used to set the respective negative gates. Stained cells were analyzed using a Cytoflex flow cytometer (Beckman Coulter). Analysis of the data was carried out using FlowJo v.10 software (Tree Star). Biological triplicates were performed for every condition. Results are presented as mean±SEM (standard error of the mean).

Pre-processing of Whole-Genome Sequencing Reads

Whole genome DNA sequencing reads (paired-end; 150bp) of human embryonic stem cell line HS980, hESC SKO-B2M and hESC DKO cells were aligned to the human reference genome (NCBI reference genome GRCh37 based “human_g1k_v37”) using the Burrows-Wheeler Aligner (BWA-0.7.12) (Li and Durbin, 2010). Sequencing data is available upon request. Duplicate reads in reference genome aligned BAM files were marked using Picard 2.9.0 “MarkDuplicates” utility. Next, we followed the “GATK Best Practice” guidelines (DePristo et al., 2011; Van der Auwera et al., 2013) using GATK 3.7 toolkit and performed local realignments and base quality recalibration with default parameters. GATK resource bundle “b37” that include data sets from HapMap, Omni, Mills Indels, 1000 Genome Indels and dbSNP v138 databases were used in this analysis. Further, germline SNVs and indels were identified using HaplotypeCaller with default parameters.

Identification of Somatic SNVs and Copy Number Deletions

We considered non-redundant whole exonic intervals (GRCh37 gene annotation) and predicted off-target sites for SNV analysis. Separate list of potential off-targets sites for hESC SKO-B2M and hESC DKO experiments were created using Cas-OFFinder (Bae et al., 2014) (up to nine mismatches allowed and up to 2bp bulge formation of either RNA or DNA) and E-CRISP (Heigwer et al., 2014) (not allowed for any 5’ mismatches to be ignored and tolerated edit distance to the target sequence set to three) online prediction tools. We used ‘NGG’ as PAM motif sequence and 5’-GGCCGAGATGTCTCGCTCCG-3’ and 5’-GAGATTGAGCTCTACTCAGG-3’, as B2M and CIITA sgRNA sequences, respectively, for off-target sites prediction. All exonic intervals and predicted off-target sites (75 bases flanking both ends) were searched for SNVs using GATK 3.7 MuTect2 utility (Cibulskis et al., 2013) with default parameters. SNVs with allele frequency (AF) ≥ 0.25 and read depth (DP) > 10 were considered for further analysis. Somatic copy number deletions were then considered since in principle a copy number deletion refers to loss of large genomic segment and a predicted off-target site could be completely missed if it is within copy number deleted regions. To ensure that such deletions have not eliminated a potential off-target site we performed copy number deletion and loss of heterozygosity (LOH) analysis using VarScan 2.3.9 (Koboldt et al., 2012) with default parameters. Briefly, we performed pair-wise somatic SNVs, copy number deletion and LOH analysis between hESC WT (HS980) vs hESC SKO-B2M, and hESC SKO-B2M vs hESC DKO samples. Heterozygous copy number deletions (fold change ≥ 1.5) overlapping with LOH regions were considered for further downstream analysis. Overlapping study of predicted off-targets with copy number deletions was performed using UCSC Galaxy bed-tools “intersect intervals” utility.

Quantitative Real-time PCR

Total RNA was isolated using the RNeasy Plus Mini Kit (Qiagen, 74106) and treated with RNase-free DNase (Qiagen, 79254) and RNaseH (ThermoFisher Scientific,18021071) according to manufacturer's protocol. cDNA was synthesized using 1 µg of total RNA in a 20 µL reaction mixture containing random hexamers (ThermoFisher Scientific, N8080127) and Superscript III reverse transcriptase (ThermoFisher Scientific, 18080085), according to the manufacturer's instructions.

Taq-polymerase (ThermoFisher Scientific, 4304437) together with Taqman probes (ThermoFisher Scientific) for *GAPDH* (4333764F), *NANOG* (Hs02387400_g1), *POU5F1/OCT4* (Hs03005111_g1), *BEST1* (Hs00188249_m1), *RPE65* (Hs01071462_m1), *HLA-A* (HS01058806_g1), *HLA-B* (HS00818803_g1), *HLA-C* (HS00740298_g1), *HLA-E* (HS03045171_m1), *HLA-DP* (HS00410276_m1), *HLA-DQ* (HS03007426_mH), *HLA-DR* (HS00219575_m1), *B2M* (HS00187842_m1) and *CIITA* (HS00172106_m1) were used. Samples were subjected to real-time PCR amplification protocol on StepOneTM real-time PCR System (Applied Biosystems). Biological triplicates were performed for every condition and technical triplicates were carried out for each sample. Results are presented as mean±SEM (standard error of the mean).

Western Blot

Total protein was extracted from cells by dissolving the collected cell pellet in cold RIPA buffer (Sigma, R0278) and incubating on ice for 5 min before centrifugation at 2000g for 5 min. The supernatant was transferred to a clean tube and protein concentration was measured using the Pierce™ BCA protein assay (Thermo Fisher Sicentific, 23227) according to the manufacturer's protocol. A total of 223 µg protein was loaded on a 4-20% TGX gel (Bio-Rad, 4568094) after being incubated in 1X Laemmli sample buffer (Bio-Rad, 161-0737), supplemented with 2-mercaptoethanol (ThermoFisher Scientific, 31350-010), at 95°C for 5 min. Following electrophoresis (120V for 70-80 min at room temperature), blotting (100V for 60 min at room temperature) was carried out using an 0.2 µM PVDF membrane (Thermo Fisher Sicentific; LC2002). Primary antibodies for B2M (1:200, Santa Cruz Biotech, B2M-02), HLA-I (1:500, Abcam, ab70328) and GAPDH (1:10000, Abcam, ab8245) were diluted in 3% BSA (Sigma, A9418) in 0.05% Tween-20 (Sigma, T8787) in DPBS (ThermoFisher Scientific, 14190-094). Primary antibody incubation was done on a rotator at 4°C overnight. The membrane was washed 3 x 10 min using 0.05% Tween-20 (Sigma, T8787) in DPBS (ThermoFisher Scientific, 14190-094), which was followed by secondary antibody incubation at room temperature for 1 hour using Alexa-680 (1:10000, ThermoFisher Scientific, A10038). The membrane was then washed 3 x 10 min with 0.05% Tween-20 (Sigma, T8787) in DPBS (ThermoFisher Scientific, 14190-094) before the membrane was imaged using LI-COR

Odyssey infrared imager (LI-COR).

Flow Cytometry

After one month in culture, hESC-RPE were dissociated into single cells using TrypLE Select (ThermoFisher Scientific, 12563-011) as described above. Samples were stained using the following conjugated antibodies: mouse anti-human HLA-ABC-FITC (1:20, BD Biosciences, 555552), mouse anti-human HLA-DR-V500 (1:66, BD Biosciences, 561224), mouse anti-human PD-L1-PE (1:30, Biolegend 329706, clone [29E.2A3]), mouse anti-human PD-L2-APC (1:20, BD Biosciences, 557925, clone [MIH18]), mouse anti-human CD80-PE (1:20, BD Biosciences, 557227, clone [L307.4]), mouse anti-human CD86-FITC (1:20, BD Biosciences, 555657, clone [2331, FUN-1]), mouse anti-human HLA-ABC-BV421 (1:100, BD Biosciences, 565332, clone [G46-2.6]), mouse anti-human HLA-A2-BV510 (1:100, BioLegend 343319, clone [BB7.2]), mouse anti-human HLA-C-PE (1:20, BD Biosciences, 566372, clone [DT-9]), mouse anti-human MICAB-AF488 (1:100, BioLegend 320912, clone [6D4]), mouse anti-human CD112-PeCy7 (1:100, BioLegend 337413, clone [TX31]), mouse anti-human CD155-BV605 (1:100, BD Biosciences, 745215, clone [TX24]), mouse anti-human PCNA-AF647 (1:100, BioLegend 307912, clone [PC10]), mouse anti-human CD158b/2DL2/L3/S5-BV711 (1:50, BD Biosciences, 743454, clone [CH-L]), mouse anti-human CD158F/2DL5-APC (1:100, MACS, 130-098-567), mouse anti-human CD158E1/3DL1-APC-Fire750 (1:50, Biolegend, 312722, clone [DX9]), mouse anti-human 3DL2-PE (1:100, R&D, FAB2878P), mouse anti-human CD158a,h/2DL1-PC7 (Beckman Coulter), human anti-human CD159a/NKG2A-FITC (1:400, MACS, 130-105-646), mouse anti-human CD107a-BV421 (1:100, Biolegend, 328626, clone [H4A3]), mouse anti-human CD47 (Human Cell Surface Marker Screening Panel, BD Biosciences, 560747, clone [B6H12]), mouse anti-human CD55 (Human Cell Surface Marker Screening Panel, BD Biosciences, 560747, clone [IA10]), mouse anti-human CD59 (Human Cell Surface Marker Screening Panel, BD Biosciences, 560747, clone [p282 (H19)]) diluted in 2% FBS (ThermoFisher Scientific, 10082147 and 1mM EDTA (Sigma, E7889) DPBS (ThermoFisher Scientific, 14190-094) or in Brilliant Buffer (BD Biosciences, 563794). Cells were incubated with the conjugated antibodies at 4°C for 30 min and washed twice with 2% FBS (ThermoFisher Scientific, 10082147) and 1mM EDTA (Sigma, E7889) diluted in DPBS (ThermoFisher Scientific, 14190-094). For CD47, CD55 and CD59 antibodies, an extra 30 min incubation step with secondary antibody Alexa Fluor 647 donkey anti-mouse IgG (Human Cell Surface Marker Screening Panel, BD Biosciences, 560747) was performed. Fluorescence minus one (FMO) or isotype mIgG controls were included for each condition to gate negative and positive cells. 7-AAD-PeCy5 (1:200, BD Biosciences, 51-68981E) or Zombie-APC-H7 NIR (1:500, Biolegend, 423105) was added to the cells for Live/Dead stain according manufacturer instructions. Respective Geometric

Mean Fluorescence Intensity (GMFI) values of the FMO controls were subtracted to obtain the final GMFI of each fluorophore. Stained cells were analyzed using a Cytoflex flow cytometer (Beckman Coulter). Analysis of the data was carried out using FlowJo v.10 software (Tree Star). Biological triplicates were performed for every condition. Results are presented as mean±SEM (standard error of the mean).

Immunocytofluorescence

Protein expression of mature hESC-RPE monolayers was assessed with immunofluorescence. Cells were fixed with 4% methanol-free formaldehyde (VWR, FFCHFF22023000) at room temperature for 10 min, followed by permeabilization with 0.3% Triton X-100 (Sigma, T9284) in DPBS (ThermoFisher Scientific, 14190094) for 10 min and blocking with 4% fetal bovine serum (FBS, ThermoFisher Scientific, 10082147) and 0.1% Tween-20 (Sigma, P9416) in DPBS for 1 hour both at room temperature. Primary antibodies were diluted to the specified concentrations in blocking solution: mouse anti-human B2M (1:100, Santa Cruz, sc-41410 [B2M-02]), mouse anti-human HLA-ABC (1:100, Abcam, ab70328 [EMR8-5]), mouse anti-human HLA-DP,DQ,DR (1:100, DAKO, M0775 [CR3/43]), rabbit anti-human Nanog (1:100, Reprocell, RCAB0003P) and rabbit anti-human ZO-1 (1:100, ThermoFisher Scientific, 40-2200). The primary antibodies were incubated overnight at 4°C followed by 2 hours incubation at room temperature with secondary antibodies: Alexa Fluor 555 donkey anti-mouse IgG (1:1000, ThermoFisher Scientific, A31570) and Alexa Fluor 488 donkey anti-rabbit IgG (1:1000, ThermoFisher Scientific, A21206) diluted 1:1000 in blocking solution. Nuclei were stained with Hoechst 33342 (1:1000, ThermoFisher Scientific, Invitrogen H3570). Images were acquired with Olympus IX81 fluorescence microscope (Carl Zeiss Meditec, 10x objective). Post-acquisition analysis of the pictures was performed using ImageJ/Fiji software.

Histology and Tissue Immunostaining

Immediately after sacrifice by intravenous injection of 100 mg/kg pentobarbital (Allfatal vet. 100 mg/ml, Omnidea), the eyes were enucleated and the bleb injection area marked with green Tissue Marking Dye (TMD; Histolab Products AB, 02199). An intravitreal injection of 100 µL fixing solution (FS) consisting of 4% buffered-formaldehyde (Histolab Products AB, 02175) was performed. FS remained for 24-48 hours followed by embedding in paraffin. 4 µm serial sections were made through the TMD-labeled area and every 4 sections were stained with hematoxylin-eosin (VWR, 1051740500).

For immunostaining, a Bond III system from Leica Biosystems/Leica Triolab AB was used. Following the manufacturer’s instructions, slides were deparaffinized in xylene for 5 min, washed with Bond Wash Solution (Leica Biosystems, AR9590), pretreated with EDTA-buffer

pH9 (Leica Biosystems, AR9640) at 100°C for 20 min, washed with Bond Wash Solution (Leica Biosystems, AR9590) and blocked with perxideblock 3% (Leica Biosystems, DS9263) for 15 min at room temperature. Slides were washed with Bond Wash Solution (Leica Biosystems, AR9590) and primary antibodies diluted in Bond Antibody Diluent Solution (Leica Biosystems, AR9352) were incubated for 60 min at room temperature: rabbit anti-human nuclear mitotic apparatus protein (NuMA) (1:200, Abcam ab84680), mouse anti-human HLA-ABC (1:100, Abcam, ab70328 [EMR8-5]), mouse anti-human HLA-DP,DQ,DR (1:100, Dako, M0775 [CR3/43]), rat anti-rabbit CD3 (1:100, Abcam, ab11089, [CD3-12]), rabbit anti-human CD56 (1:200, Cellmarque, 156R-95) mouse anti-rabbit RAM11 (1:50, DAKO, M0633), mouse anti-rabbit CD79a (1:100, ThermoFisher Scientific, MA5-13212 [HM47/A9]). After a washing step with Bond Wash Solution (Leica Biosystems, AR9590), secondary antibodies (1:200, Alexa Fluor 546 goat anti-rabbit IgG (A11035), Alexa Fluor 647 donkey anti-mouse IgG (A31571), Alexa Fluor 546 goat anti-rat IgG (A11081), Alexa Fluor 647 goat anti-rabbit IgG (A21245); all from ThermoFisher Scientific) diluted 1:2000 in Bond Antibody Diluent Solution (Leica Biosystems, AR9352) were incubated for 15 min at room temperature, washed with Bond Wash Solution (Leica Biosystems, AR9590) and incubated with Hoechst 33342 solution in PBS (5g/L, Sigma, 33258) for 5 min at room temperature. After washing steps with Bond Wash Solution (Leica Biosystems, AR9590) and deionized water, sections were mounted with DAKO Fluorescence Mounting Media (DAKO, S3023) on a 24x50 mm coverslip. Images were taken with Zeiss LSM710 point scanning confocal microscope (Carl Zeiss Meditec, 20x objective). Post-acquisition analysis of the pictures was performed using ImageJ/Fiji software.

hESC-RPE and T-cell Co-culture

Firstly, day 30 (after replating) unstimulated or 2 days IFN-γ pre-stimulated (100 ng/mL, Peprotech, 300-02) hESC-RPE cells were trypsinized as described above, irradiated (30 Gy) and plated at a cell density range of $1x10^3$ (1:500) - $5.5x10^5$ (1:1) cells/cm^2 (depending on the respective experiment) on hrLN-521 coated dishes (20 μg/mL, Biolamina) using complete RPMI medium (Hyclone, SH3025501) with 10% AB serum (Sigma, H3667) and 100U/mL penicillin and 100 μg/mL streptomycin (Hyclone, SV30010). hESC-RPE cells were left for at least 3 hours to let attach to the plate. Secondly, human PBMCs were isolated from buffy coats of healthy donors by Lymphoprep (Axis-Shield PoC AS, 1114547) density gradient centrifugation. After washing with PBS, cell numbers were assessed by counting with Sysmex (Sysmex Sverige, KX-21N). PBMCs were then either stained with CellTrace CFSE Cell Proliferation Kit (2.5 μg/mL, ThermoFisher Scientific, C34554) or divided into two tubes for CD4+ and CD8+ isolation with commercially available CD4 and CD8-negative selection beads from MACS, Miltenyi Biotec (130-096-533 and 130-096-495, respectively) in

accordance with the instructions of the manufacturer. Purities were of above 90% for either CD4+ or CD8+ selected cells in the two donors used for the experiments (data not shown). Finally, 1 million of the labelled or unlabelled PBMCs, or isolated CD4+ or CD8+ T-cells were plated per well in a 24-well plate on top of the attached unstimulated or pre-stimulated hESC-RPE. IL-2 (1 ng or 100U, BD Biosciences, 554603), CD28 (1.25 μg/mL, Biolegend, 302902 [CD28.2]) or OKT-3 (25 ng/mL, Biolegend, 317315) molecules were added the wells if required. For MLR, PBMCs were isolated from different donors as described above. Irradiated (30Gy) and non-irradiated CFSE-labelled PBMCs were subsequently co-cultured with the same numbers (1 million each) and the corresponding stimulatory molecules were added if required. Co-cultures were maintained for 5 days at 37°C for further analysis.

Enzyme-Linked Immunosorbent Assay (ELISA)

Supernatants from hESC-RPE and T-cell co-cultures (either with CD8+ or CD4+ positive isolated human PBMCs) co-cultures were collected 5 days after the cells were plated. IFN-γ secretion levels were measured in triplicates for each condition with commercially available human IFN-γ ELISA Kit (Mabtech, 3420-1HP-2) in accordance with the instructions of the manufacturers. The optical density readings were measured using SpectraMax i3x Reader (MolecularDevices). Results are presented as mean±SEM (standard error of the mean).

Isolation of Human NK Cells from Healthy Donors

Human PBMCs were isolated as described above and consecutively NK cells were negatively separated with a CD56 isolation kit (Miltenyi Biotec, 130-050-401) using autoMACs Pro Separator (Miltenyi Biotec) with the “depletes” program. Purities were of above 90% for CD56+ CD3- selected cells in the three donors used for the experiments (data not shown). Final cell numbers were assessed by Türks solution (Histolab Products AB, HL22200.0100) and cells were seeded out at a concentration of $1x10^6$ cells/mL in stem cell growth medium (CellGro, CellGenix, 20802-0500) with 20% heat inactivated FBS (ThermoFisher Scientific, 10500064) and activated over night with 500 U/mL IL-2 (R&D Systems, 202-GMP-01M).

Cytotoxicity Assay

NK mediated cytotoxicity was measured in a ^{51}Cr-release assay with overnight IL-2 activated hNK cells (effector cells) against unstimulated or 2 days IFN-γ pre-stimulated (100 ng/mL, Peprotech, 300-02) hESC-RPE. hESC-RPE (target cells) were labeled with 70 μCi ^{51}Cr (PerkinElmer, Waltham, MA) for 1 hour at 37°C, and NK cells were then mixed with the labeled target cells at different effector:target ratios (10:1; 3:1; 1:1; 0.3:1) in a 96-well plate

and incubated for 4 hours at 37°C. After, supernatants (25 µL) were transferred into LumaPlate-96 Deep-Well and analyzed using a MicroBeta^2 LumiJET Microplate counter (PerkinElmer, Waltham, MA). *Percentage of specific lysis per sample type = [(experimental - spontaneous release) / (maximum load - spontaneous release) x 100].* Results are presented as mean±SEM (standard error of the mean).

Degranulation Assay

NK in vitro response was measured in a 4h degranulation assay with overnight IL-2 activated hNK cells (effector cells) against unstimulated or 2 days IFN-γ pre-stimulated (100 ng/mL, Peprotech, 300-02) hESC-RPE (target cells). NK cells were mixed with target cells at an effector:target ratio of 3:1 in a 96-well plate in the presence of anti-CD107 mAb and incubated at 37°C. After 1 hour, monensin (GolgiStop, BD Biosciences, 554724) was added to block protein transport, and cells were incubated for additional 3h at 37°C. Cells were stained for the indicated surface molecules and subsequently for intracellular cytokines (IFN-γ) using BD Cytofix/Cytoperm kit (BD Biosciences, 554714), according to manufacturer recommendations. Flow Cytometry was performed as described above and cells were acquired on a BD Symphony (Becton Dickinson). Analysis of the data was carried out using FlowJo v.10 software (Tree Star). Biological triplicates were performed for every condition. Results are presented as mean±SEM (standard error of the mean).

Rabbit Serum Collection

5-10 mL of blood was extracted from the rabbits prior and after subretinal injections at different time points (d7, d14, d30 and d90) in serum collection tubes (Vacuette Z Serum Sep Clot Activator, Greiner Bio-One, 455010). The tubes were rested in a standing position for about 15-20 min until blood was clotted, and then centrifuged at 20°C, 1500g for 10 min. Serum (supernatant) was quickly removed and stored at -80°C in 1mL aliquots for further analysis.

Antibody-mediated Assay

100,000 cultured WT hESC-RPE cells were pelleted and mixed with 100 µL of serum for 30 min at room temperature. After two washes with 2% FBS (ThermoFisher Scientific, 10082147) and 1mM EDTA (Sigma, E7889) in DPBS (ThermoFisher Scientific, 14190-094) at 300g for 5 min, secondary antibody donkey anti-rabbit A488 (ThermoFisher Scientific, A21206/R37118) diluted 1:1000 was added for 20 min at 4°C and washed two extra times (300g, 5 min). Finally, 7-AAD-PeCy5 (1:200, BD Biosciences, 51-68981E) Live/Dead stain diluted in 2% FBS (ThermoFisher Scientific, 10082147) and 1mM EDTA (Sigma, E7889) in DPBS (ThermoFisher Scientific, 14190-094) was added to the cells. Stained cells were

analyzed using a Cytoflex flow cytometer (Beckman Coulter). Analysis of the data was carried out using FlowJo v.10 software (Tree Star). Results are presented as mean±SEM (standard error of the mean).

Animals

After approval by the Northern Stockholm Animal Experimental Ethics Committee (DNR N25/14), female New Zealand white albino rabbits (provided by Lidköpings rabbit farm, Lidköping, Sweden) aged 5 months and weighing 3.5 to 4.0 kg were used in this study. All experiments were conducted in accordance with the Statement for the Use of Animals in Ophthalmic and Vision Research.

Subretinal Transplantation

hESC-RPE monolayers were washed with DPBS (ThermoFisher Scientific, 14190-094), incubated with TrypLE (ThermoFisher Scientific, 12563-011) and dissociated to single cell suspension as described above. Cells were counted in a Neubauer hemocytometer (VWR, 631-0925) chamber using 0.4% trypan blue (ThermoFisher Scientific, 15250061), centrifuged at 300g for 4 min, and the cell pellet was resuspended in freshly filter-sterilized DPBS (ThermoFisher Scientific, 14190-094) to a final concentration of 1000 cells/µL. The cell suspension was then aseptically aliquoted into 600 µL units and kept on ice until surgery.

Animals were put under general anesthesia by intramuscular administration of 35 mg/kg ketamine (Ketaminol 100 mg/mL, Intervet, 511519) and 5 mg/kg xylazine (Rompun vet. 20 mg/mL, Bayer Animal Health, 22545), and the pupils were dilated with a mix of 0.75% cyclopentolate / 2.5% phenylephrine (APL, 321968). Microsurgeries were performed on both eyes using a 2-port 25G transvitreal pars plana technique (Alcon Nordic A/S, 8065751448). 25G trocars were inserted 1 mm from the limbus and an infusion cannula was connected to the lower temporal trocar. The cell suspension was drawn into a 1 mL syringe connected to an extension tube and a 38G polytip cannula (MedOne Surgical Inc, 3219 and 3223). Without infusion or prior vitrectomy the cannula was inserted through the upper temporal trocar. After proper tip positioning, ascertained by a focal whitening of the retina, 50 µL of cell suspension (equivalent to 50,000 cells) was injected slowly subretinally approximately 6 mm below the inferior margin of the optic nerve head, forming a uniform bleb that was clearly visible under the operating microscope. To minimize reflux, the tip was maintained within the bleb during the injection. After instrument removal light pressure was applied to the self-sealing suture-less sclerotomies. 2 mg (100 µL) of intravitreal triamcinolone (Triescence 40 mg/mL, Alcon Nordic A/S, 412915) was administered a day prior to the surgery, and no post-

surgical antibiotics were given. For the TCA cohort, intravitreal triamcinolone was re-administered every 3 months, if required.

Multimodal Real-time Imaging and Choroidal and Subretinal Thickness Measurements

Multimodal real-time imaging was performed as described before (Bartuma et al., 2015). Briefly, anesthetized animals were placed in an adjustable mount (Spectralis HRA + OCT device, Heidelberg Engineering with the Heidelberg Eye Explorer software) to obtain horizontal cross-sectional b-scans of treated animals. *En-face* fundus images were obtained by multicolor cSLO. For thickness measurements, SD-OCT scans were randomly acquired through the upper, middle and lower segments of the subretinal transplantation area, and the scan with the largest subretinal infiltrate and simultaneous choroidal thickening was chosen for analysis. The height of the subretinal infiltrate (from the Bruch's membrane to the outer border of the neurosensory retina) was measured at the thickest point using ImageJ/Fiji. To obtain the value for choroidal thickening, the total thickness of the choroid was measured at the same position and subtracted from the choroidal thickness outside the transplantation area (naive choroid). Similarly, for evaluation of rejection rates, SD-OCT scans were randomly acquired as for choroidal measurement, and if any subretinal infiltrate and choroidal thickening was observed in any of the scans the transplant was scored as rejected.

Statistical Analysis

For statistical analyses, Chi-squared test was used to compare differences in rejection rates in the three subretinally transplanted groups with WT, SKO-B2M, SKO-CIITA and DKO cells. ANOVA (one-way or two-way) and posthoc multiple comparisons using Tukey test were performed to assess the *in vitro* differences of the different knock out lines upon co-culture with immune cells, and to assess anti-human antibody presence upon subretinal injection in the xenograft model.

REFERENCES

Bae, S., Park, J., and Kim, J.S. (2014). Cas-OFFinder: a fast and versatile algorithm that searches for potential off-target sites of Cas9 RNA-guided endonucleases. Bioinformatics *30*, 1473-1475.

Bartuma, H., Petrus-Reurer, S., Aronsson, M., Westman, S., Andre, H., and Kvanta, A. (2015). In Vivo Imaging of Subretinal Bleb-Induced Outer Retinal Degeneration in the Rabbit. Invest Ophthalmol Vis Sci *56*, 2423-2430.

Cibulskis, K., Lawrence, M.S., Carter, S.L., Sivachenko, A., Jaffe, D., Sougnez, C., Gabriel, S., Meyerson, M., Lander, E.S., and Getz, G. (2013). Sensitive detection of somatic point mutations in impure and heterogeneous cancer samples. Nat Biotechnol *31*, 213-219.

DePristo, M.A., Banks, E., Poplin, R., Garimella, K.V., Maguire, J.R., Hartl, C., Philippakis, A.A., del Angel, G., Rivas, M.A., Hanna, M.*, et al.* (2011). A framework for variation discovery and genotyping using next-generation DNA sequencing data. Nat Genet *43*, 491-498.

Heigwer, F., Kerr, G., and Boutros, M. (2014). E-CRISP: fast CRISPR target site identification. Nat Methods *11*, 122-123.

Koboldt, D.C., Zhang, Q., Larson, D.E., Shen, D., McLellan, M.D., Lin, L., Miller, C.A., Mardis, E.R., Ding, L., and Wilson, R.K. (2012). VarScan 2: somatic mutation and copy number alteration discovery in cancer by exome sequencing. Genome Res *22*, 568-576.

Li, H., and Durbin, R. (2010). Fast and accurate long-read alignment with Burrows-Wheeler transform. Bioinformatics *26*, 589-595.

Liu, J., Jones, K.L., Sumer, H., and Verma, P.J. (2009). Stable transgene expression in human embryonic stem cells after simple chemical transfection. Mol Reprod Dev *76*, 580-586.

Ran, F.A., Hsu, P.D., Wright, J., Agarwala, V., Scott, D.A., and Zhang, F. (2013). Genome engineering using the CRISPR-Cas9 system. Nat Protoc *8*, 2281-2308.

Rodin, S., Antonsson, L., Hovatta, O., and Tryggvason, K. (2014a). Monolayer culturing and cloning of human pluripotent stem cells on laminin-521-based matrices under xeno-free and chemically defined conditions. Nat Protoc *9*, 2354-2368.

Rodin, S., Antonsson, L., Niaudet, C., Simonson, O.E., Salmela, E., Hansson, E.M., Domogatskaya, A., Xiao, Z., Damdimopoulou, P., Sheikhi, M.*, et al.* (2014b). Clonal culturing of human embryonic stem cells on laminin-521/E-cadherin matrix in defined and xeno-free environment. Nat Commun *5*, 3195.

Van der Auwera, G.A., Carneiro, M.O., Hartl, C., Poplin, R., Del Angel, G., Levy-Moonshine, A., Jordan, T., Shakir, K., Roazen, D., Thibault, J., *et al.* (2013). From FastQ data to high confidence variant calls: the Genome Analysis Toolkit best practices pipeline. Curr Protoc Bioinformatics *43*, 11 10 11-33.
